# A taxonomic revision of the Archipini of the Caribbean (Lepidoptera, Tortricidae, Tortricinae)

**DOI:** 10.3897/zookeys.982.52363

**Published:** 2020-11-02

**Authors:** Kyhl A. Austin, Jason J. Dombroskie

**Affiliations:** 1 Department of Entomology, Comstock Hall, Cornell University, Ithaca, NY, 14853, USA Cornell University Ithaca United States of America; 2 Department of Plant and Environmental Protection Sciences, Gilmore Hall, University of Hawai'i at Mānoa, Honolulu, HI, 96822, USA University of Hawaiʻi at Mānoa Honolulu United States of America

**Keywords:** *
Argyrotaenia
*, biogeography, *
Claduncaria
*, *
Clepsis
*, *
Mictocommosis
*, *
Mictopsichia
*, new species, *
Rubropsichia
*

## Abstract

The Archipini fauna of the Caribbean is revised to include 33 species. Most previously described species occurring in the region are redescribed and figured, with 13 new species: *Argyrotaenia browni***sp. nov.**, *A. cryptica***sp. nov.** (including *A. c. cryptica***ssp. nov.** and *A. c. praeteritana***ssp. nov.**), *A. paradisei***sp. nov.**, *A. razowskiana***sp. nov.**, *Claduncaria rawlinsana***sp. nov.**, *Cla. praedictana***sp. nov.**, *Cla. taino***sp. nov.**, *Clepsis davisi***sp. nov.**, *Cle. deroni***sp. nov.**, *Cle. jamesstewarti***sp. nov.**, *Cle. peroniae***sp. nov.**, *Mictocommosis lesleyae***sp. nov.**, and *Mictopsichia nyhllinda***sp. nov.** Three new combinations are proposed: *Claduncaria mesosignaria* (Razowski, 1999), **comb. nov.** (including *Argyrotaenia thamaluncus* Razowski, 1999, **syn. nov.**), *Claduncaria minisignaria* (Razowski, 1999), **comb. nov.**, and *Claduncaria chalarostium* (Razowski & Becker, 2000b), **comb. nov.**, **stat. nov.***Argyrotaenia granpiedrae* Razowski & Becker, 2010 is reduced to subspecies rank under *Argyrotaenia ceramica* Razowski, 1999, resulting in *Argyrotaenia ceramica granpiedrae* Razowski & Becker, 2010, **stat. nov.** Four new synonymies are proposed: *Clepsis labisclera* Razowski & Becker, 2010, **syn. nov.** as junior synonym of *Claduncaria maestrana* Razowski & Becker, 2010; *Clepsis pinaria* Razowski & Becker, 2010, **syn. nov.** as junior synonym of *Clepsis peritana* (Clemens, 1860); and *Argyrotaenia neibana* Razowski, 1999, **syn. nov.** and *A. ochrochroa* Razowski, 1999 **syn. nov.** as junior synonyms of *Argyrotaenia amatana* (Dyar, 1901). Males of *Argyrotaenia felisana* Razowski, 1999, *A. nuezana* Razowski, 1999, and *Claduncaria minisignaria* (Razowski, 1999), **comb. nov.** are described for the first time; females of *Argyrotaenia jamaicana* (Razowski & Becker, 2000a) and *Claduncaria ochrochlaena* (Razowski, 1999) are described for the first time. The concept of *Claduncaria* is expanded and its diagnosis is modified to more clearly define its generic boundaries. A unique external sexual coupling mechanism in *Claduncaria* is discussed. Keys to the genera and species of Caribbean Archipini, distribution maps, a regional checklist, and Neighbor-joining and Maximum Likelihood trees based on COI barcode data are provided. Phylogenetic relationships among Caribbean Archipini are briefly discussed.

## Introduction

Archipini is the most diverse tribe in the family Tortricidae; Brown (2005) recorded 2003 species in 230 genera worldwide. Subsequent papers have only further added to these numbers. The tribe is most diverse in the Australasian region, least diverse in the Neotropical region, and contains some of the most economically important tortricid pest species on the planet (e.g., *Epiphyas postvittana*, the light brown apple moth; *Choristoneura* spp., spruce budworms; *Archips argyrospila*, the fruit-tree leafroller) (Dombroskie and Sperling 2013). The Archipini fauna of the Caribbean is poorly known, with taxonomic treatments restricted to single islands or archipelagos (Razowski 1999; Austin et al. 2019), or as species included as elements of broader systematic revisions (Austin and Dombroskie 2020). The purpose of this revision is to synthesize the information available on Caribbean Archipini by describing new species, proposing new synonymies, redescribing and illustrating previously described species, describing the opposite sex of several species, and noting new distributional records.

A major obstacle in the study of Archipini has been the lack of taxonomically useful characters in the genitalia. For example, males of many *Clepsis* species and females of many *Argyrotaenia* species are virtually indistinguishable from their congeners. Compounding this is the presence of marked sexual dimorphism in some genera, making reliable association between sexes difficult, if not impossible based on morphology alone. Historically, much emphasis has been placed on forewing pattern and geographic distribution in diagnoses. However, we have found that though often subtle, there are features in the genitalia of both sexes that are useful for reliably identifying species. In females, the shape of the papillae anales is often discounted as being too variable to be useful; however, the opposite may be true. In fact, in some species of *Argyrotaenia*, the shape of the papillae anales is one of the most useful features in identification. In addition, the capitulum and signum are also very informative and usually consistent within a species. For males, shape of the valvae, phallus, and uncus are usually consistent in shape within species. In addition to these structures, we have also found the shape of the terminal plate of the gnathos and width of the presaccular gap (defined below) to be particularly informative.

A putative synapomorphy for Archipini is the presence of a well-developed uncus with apicoventral setae (“uncus brush” *sensu*Horak 1984) in the males, although this appears to be present in at least two other lineages as well (Epitymbiini, Ceracini) (Horak 1984). Most, but not all, females possess a prominent blade- or sickle-shaped signum. The tribe, as it is currently defined, is polyphyletic, composed of several derived and plesiomorphic lineages and will require careful work to render into monophyletic entities (Horak 1984, 1999). The circumscription of Archipini is an important one to consider, for both phylogenetic and economic reasons, but resolution of this problem is beyond the scope of the present paper, so we refer the reader to Horak (1984, 1999) for further information.

It is the presence of such a blade- or sickle-shaped signum in the *Mictopsichia* group (*Chamaepsichia*, *Compsocommosis*, *Mictocommosis*, *Mictopsichia*, *Nexosa*, *Rubropsichia*) that has resulted in their assignment to Archipini (Razowski 2009; Heppner and Bae 2015a, b). Prior to this, they were included in Glyphipterigidae (Meyrick 1912, 1920, 1921, 1932), Hilarographini (Tortricidae, Chlidanotinae) (Diakonoff 1977), or treated as a new tribe (Brown 2005). We find the placement of the *Mictopsichia* group of genera in Archipini to be questionable, as not all these genera possess the typical archipine signum. Although superficially similar in wing pattern, the development, shape, and presence/absence of important male genitalic structures vary wildly among these genera, leading us to believe that this group is an artificial assemblage of several unrelated diurnal lineages with convergent wing patterns.

Nevertheless, in the present work, we include this group for continuity, recognizing that they likely belong elsewhere and may represent several different unrelated taxa. Before correct tribal assignments for members of the *Mictopsichia* group can be determined, the precise composition of these genera will require resolution. Hence, we treat species of this group herein according to current generic concepts, as describing new genera for mostly non-Caribbean species is beyond the scope of this paper.

With few exceptions, Archipini in the Caribbean are restricted to mid- to high elevations (excluding the *Mictopsichia* group). This habitat preference, combined with the topographic complexity of the Caribbean islands, has driven high levels of endemism and surprisingly high levels of species richness for such a small geographic area. On Hispaniola, for example, there are four disjunct mountain ranges, with some peaks around 2000 m in elevation. The intervening valleys provide extreme topographic relief; e.g., the Hoya de Enriquillo valley between the Sierra de Bahoruco and the Sierra de Neiba has several points below sea level. This serves to create several smaller “islands” on Hispaniola itself, with the intervening “seas” (i.e., the valleys) inhospitable to montane archipine species. The majority of Caribbean archipine species are restricted to a single mountain range, and in some cases, to a single peak or series of closely situated peaks, raising questions about their conservation prospects. Of the non-*Mictopsichia* group of archipines, only five have been recorded from coastal elevations, and five are known from more than one island or archipelago.

The islands of the Caribbean provide an excellent realm in which to study insect biogeography, as demonstrated by the attention it has received from entomologists (see Liebherr 1988). Unfortunately, only rarely have Caribbean microlepidoptera been examined (Davis 1975; Heppner 1985; St. Laurent & McCabe 2016).

There exist no comprehensive Caribbean-centric revisions for any tortricid groups. Recent papers have begun to shed light on Caribbean tortricid diversity, but these have all been part of broader, Neotropical generic revisions (Razowski and Becker 2000b; Adamski and Brown 2001; Brown and Brown 2004; Phillips-Rodriguez and Powell 2007; Brown 2008; Razowski and Brown 2008; Brown 2009; Razowski and Becker 2010; Brown et al. 2018), isolated taxonomic treatments (Matthews et al. 2012, 2019; Brown et al. 2018; Gilligan et al. 2018; Austin et al. 2019), or faunal inventories of the Lepidoptera in general (Núñez-Aguila & Barro-Cañamero 2012; Perez-Gelabert 2020). The present paper represents the first comprehensive taxonomic revision of a Caribbean tortricid tribe.

## Materials and methods

Dissection methods follow Landry (2007); however, for some dissections slide-mounting was delayed to allow lateral imaging of the male genitalia. Genitalia and abdomens, when not permanently slide mounted, are preserved in glycerol-filled microvials pinned beneath the specimen. Genitalia were stained with a combination of Eosin Y and chlorazol black. Forewing length (FWL) was measured in a straight line from the base of the costa to the apex including the fringe to the nearest half-millimeter.

Images of adults and genitalia were captured using a Macroscopic Solutions Macropod Pro and Canon EOS 6D DSLR camera body using the Macro Photo MP-E 65 mm f/2.8 1–5× manual focus lens for EOS or EF 70–200 mm zoom lens with 10× or 20× Mitutoyo objective lenses for genitalia. Images were stacked as needed using Zerene Stacking Software Version 1.04 (Zerene Systems, LLC 2014). Figures were manipulated with Adobe Photoshop CC (2018). Maps were created with SimpleMappr (Shorthouse 2010) and further manipulated with Adobe Photoshop CC (2018). Coordinates, when not included on data labels, were estimated based on locality information available to create maps. Specimens that were not examined by KAA but are still based on reliable identifications by JJD or others are excluded from material examined but included in maps and listed in Tab 1 of Suppl. material 1.

**Figure 1.**
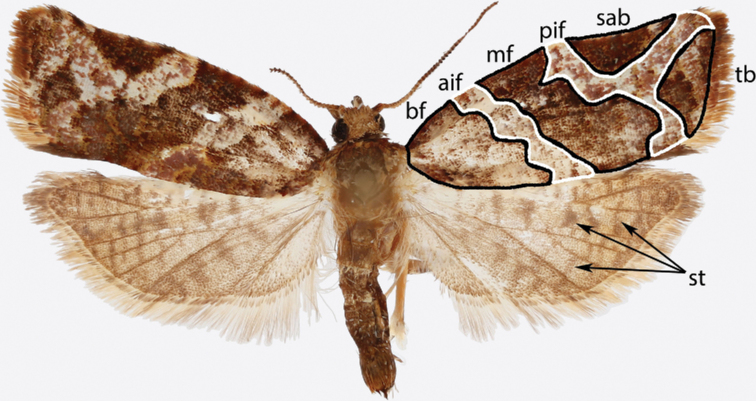
Typical Archipini wing pattern (*Argyrotaenia paradisei* sp. nov. holotype ♂). Abbreviations: *aif*, antemedian interfascia; *bf*, basal fascia; *mf*, median fascia; *pif*, postmedian interfascia; *sab*, subapical blotch; *st*, strigulae; *tb*, tornal blotch.

**Figure 2.**
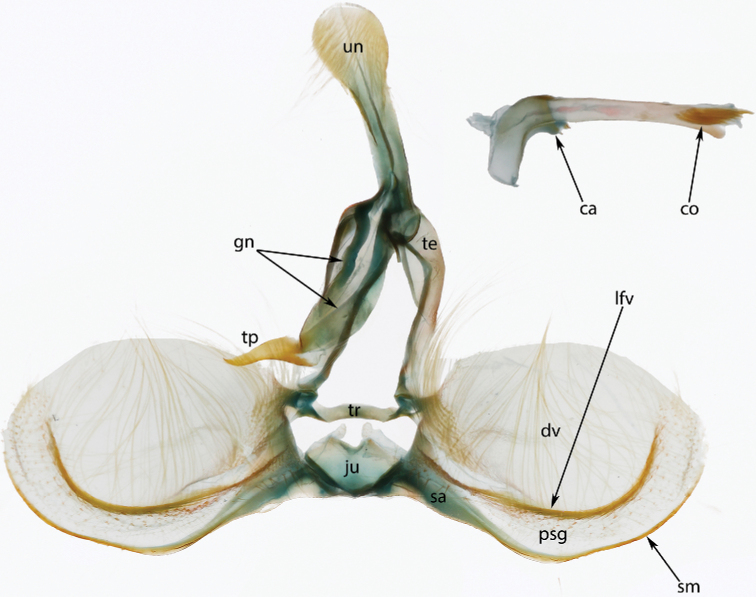
Typical Archipini male genitalia (*Argyrotaenia cryptica praeteritana* ssp. nov. paratype), phallus inset (not to scale). Abbreviations: *ca*, caulis; *co*, cornuti; *dv*, disc of valva; *gn*, gnathos; *ju*, juxta; *lfv*, longitudinal fold of valva; *psg*, presaccular gap; *sa*, sacculus; *sm*, saccular margin; *te*, tegumen; *tp*, terminal plate; *tr*, transtilla; *un*, uncus.

Morphological terms, including those for genitalia, follow Razowski (2008) with the exception of the “aedeagus”, for which we instead use “phallus” per Kristensen (2003); and “transtilla”, for which we instead use “labis” (plural “labides”) in *Clepsis* per Razowski (1979). Wing pattern terminology is illustrated in Fig. 1. In addition, we propose the term “presaccular gap”, defined as the region between the saccular margin and the longitudinal fold of the valva (“plications” *sensu*Horak 1984; Fig. 2), which is taxonomically useful in many species of *Argyrotaenia*. Some additional terms used in the treatment of *Mictocommosis*, *Mictopsichia*, and *Rubropsichia* come from Razowski (2009).

For the purposes of the present paper, we define the Caribbean to include the Lucayan Archipelago; the Greater Antilles, including the Cayman Islands; and the Lesser Antilles, excluding Trinidad & Tobago and the Leeward Antilles because these islands lie on the South American continental shelf.

In a few instances, data labels were discovered to be incorrect. In these cases, corrected province names or coordinates have been placed in brackets immediately following the verbatim label data.

In the taxonomic treatment and figures, genera and species are arranged by similarity to facilitate comparisons. The taxonomic and regional checklists are arranged alphabetically for ease of navigation.

Keys to all genera and species known from the Caribbean are provided and based primarily on genitalia. The adults and genitalia of all described Caribbean Archipini are figured with the exceptions of *Argyrotaenia flavoreticulana* Austin & Dombroskie, 2019; *Argyrotaenia kimballi* Obraztsov, 1961; *Mictopsichia jamaicana* Razowski, 2009; and genitalia of *Clepsis peritana* (Clemens, 1860). We were unable to locate the holotype of *Mictopsichia jamaicana* Razowski, 2009. Adults and genitalia of the remaining three species were figured in Austin et al. (2019).

DNA extraction, PCR amplification, and sequencing of the COI barcode region was performed at the Canadian Centre for DNA Barcoding (CCDB) and follow NGSFT protocols (Prosser et al. 2016). Despite the age of some specimens, complete COI barcodes (658 bps) were recovered for many species and partial barcodes (> 500 bps) were recovered for most others. These were used to associated sexes and help delimit ambiguous species complexes. COI-5P sequences and voucher specimen information, along with complete data records for all specimens examined are available in Tab 1 of Suppl. material 1.

Barcoded specimens for which a unique specimen identification number was not already present (i.e., an accession number) as a label or part of a label were affixed with an additional label with a unique identification number beginning with “KAA_DNA_” and ending in a four-digit number, as well as explicitly stating that a leg was removed for DNA barcoding. These “KAA_DNA_” numbers are synonymous with BOLD sample IDs. USNM specimens each have accession numbers listed in Suppl. material 1; only barcoded USNM specimens have their accession numbers listed in the material examined sections, as they are the same as the BOLD sample IDs in these instances.

**Figure 3.**
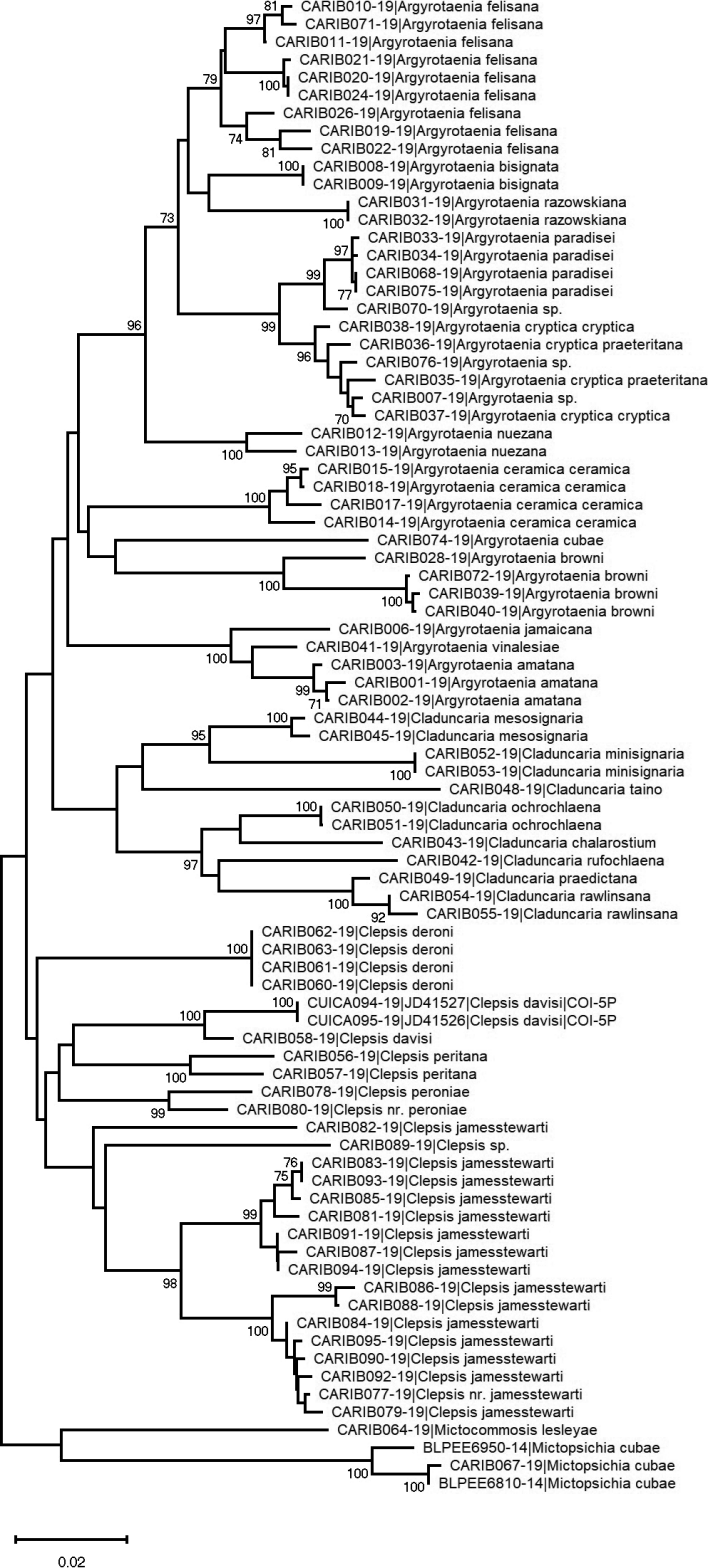
Neighbor-joining (NJ) tree inferred using the Neighbor-Joining method (Saitou and Nei 1987) from COI barcode sequence data for specimens for which > 500 base pairs recovered. The optimal tree with the sum of branch length = 1.2731 is shown. 1000 bootstrap replicates were conducted and their scores are shown next to branches. Only bootstrap scores greater than 70% are shown. Distances were computed using the Maximum Composite Likelihood (MCL) method (Tamura et al. 2004) and are in the units of the number of base substitutions per site. Analysis conducted in MEGA X (Kumar et al. 2018). BOLD process IDs and identifications are given at branch tips. Voucher specimen data and a pairwise distance matrix are given in Suppl. material 1.

**Figure 4.**
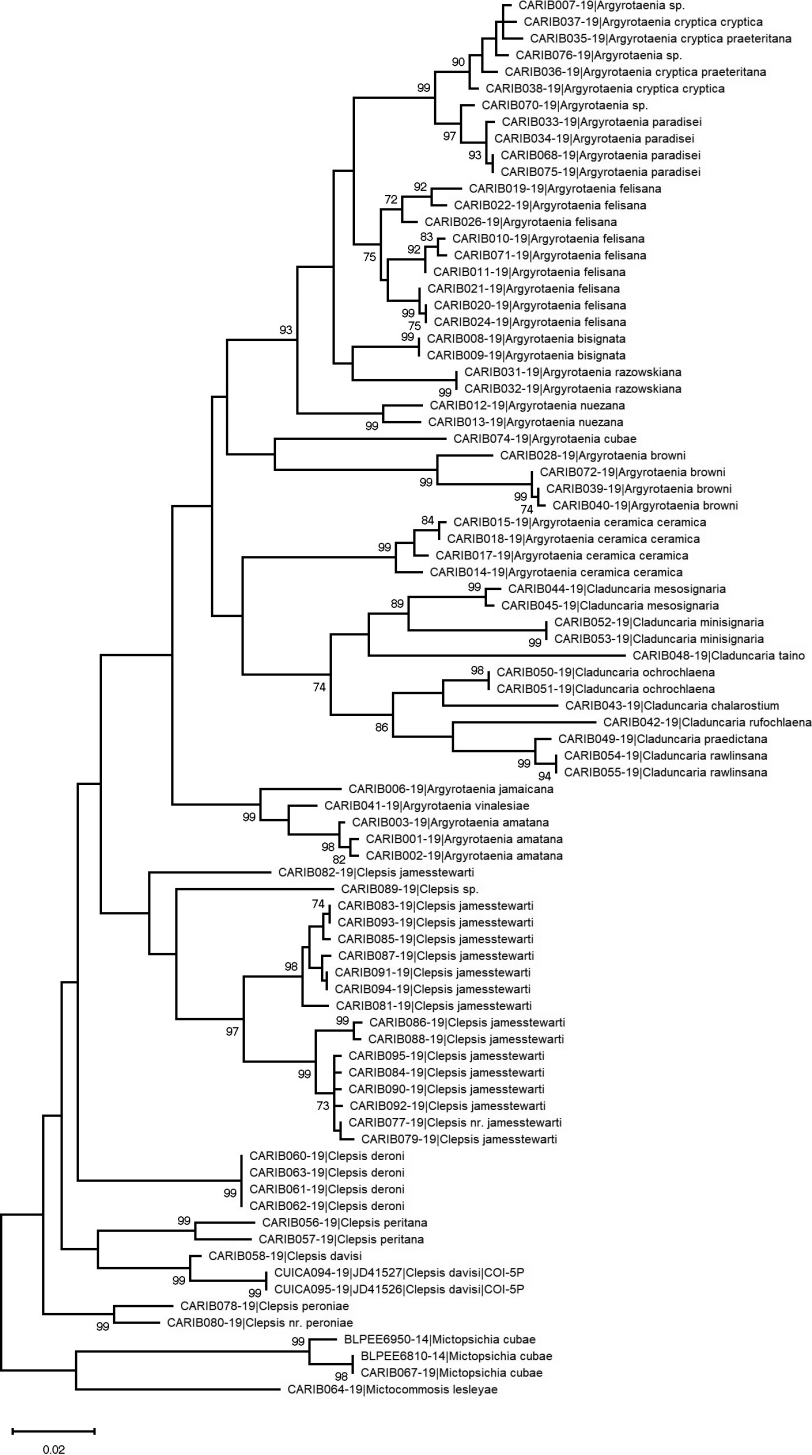
Maximum Likelihood (ML) tree inferred using the Kimura 2-parameter model (Kimura 1980) from COI barcode sequence data for specimens for which > 500 base pairs were recovered. The tree with the highest log likelihood (-5939.70) is shown. 1000 bootstrap replicates were conducted and their scores are shown next to branches. Only bootstrap scores greater than 70% are shown. This phylogenetic analysis was conducted in MEGA X (Kumar et al. 2018). BOLD process IDs and identifications are given at branch tips. Voucher specimen data and a pairwise distance matrix are given in Suppl. material 1.

Drawn-to-scale Neighbor-joining (NJ, Fig. 3) and Maximum Likelihood (ML, Fig. 4) trees of Caribbean Archipini were generated using MEGA X (Kumar et al. 2018). The ML tree was inferred using a Kimura 2-parameter model (Kimura 1980). Initial trees for the heuristic search were obtained automatically by applying Neighbor-Joining and BioNJ algorithms to a matrix of pairwise distances estimated using the Maximum Composite Likelihood (MCL) approach, and then selecting the topology with superior log likelihood value. The NJ tree was generated using the Neighbor-Joining method (Saitou and Nei 1987). Distances were computed using the Maximum Composite Likelihood (MCL) method (Tamura et al. 2004) and are in the units of the number of base substitutions per site. Both analyses were run with 1000 bootstrap replicates for sequences for which > 500 bp were recovered (n = 84, representing 27 different species). *Mictopsichia* and *Mictocommosis* were used as outgroups for both analyses as neither genus likely belongs to Archipini (see introduction). A pairwise distance matrix of all sequenced specimens is available in Tab 2 of Suppl. material 1 and was also computed using MEGA X (Kumar et al. 2018).

Because of the extremely limited taxon coverage, the trees were used to delineate species boundaries and examine terminal clades in the Caribbean, not as an attempt to accurately reconstruct a phylogeny. That said, these trees may help to understand basic relationships among Caribbean taxa, recognizing that future sampling is necessary.

Specimens from the following collections were examined:

**AMNH**American Museum of Natural History, New York, NY, USA;

**BMNH**British Museum of Natural History, London, UK;

**CMNH**Carnegie Museum of Natural History, Pittsburgh, Pennsylvania, USA;

**CUIC**Cornell University Insect Collection, Ithaca, New York, USA;

**FSCA**Florida State Collection of Arthropods, Gainesville, Florida, USA;

**ISEZ**Institute of Systematics and Evolution of Animals, Polish Academy of Sciences, Kraków, Poland;

**MGCL** McGuire Center for Lepidoptera & Biodiversity, Gainesville, Florida, USA;

**MEM** Mississippi Entomological Museum, Starkville, Mississippi, USA;

**TM** Research collection of Tim L. McCabe, Albany, New York, USA;

**USNM**National Museum of Natural History, Washington D.C., USA;

**VBC** Vitor Becker Collection, Camacan, Bahia, Brazil.

The following abbreviations are used:

**diss.** dissection;

**FW** forewing;

**FWL** forewing length;

**HW** hindwing.

## Results

### Key to genera of Caribbean Archipini^1^

**Table:** 

1	Wing pattern telechromatic (Fig. 13)	**2**
–	Wing pattern not telechromatic (Figs 5–12)	**4**
2	Antennae thickened, single row of scales per segment; male with uncus well developed (Fig. 20E); female with ductus bursae coiled (Fig. 21E)	*** Mictocommosis ***
–	Antennae narrow, two rows of scales per segment; male with uncus obsolete (Fig. 20D, F, G); female with ductus bursae not coiled (Fig. 21F, G)	**3**
3	Male genitalia with socii fused (Fig. 20D)	*** Rubropsichia ***
–	Male genitalia with socii free (Fig. 20F, G)	*** Mictopsichia ***
4	Male genitalia with transtilla incomplete, spinulate labides present instead (Fig. 20A–C); female genitalia with ductus bursa coiled (Fig. 21A, B, D), sometimes only loosely so (Fig. 21C)	*** Clepsis ***
–	Transtilla complete (Figs 14, 15, 18); female genitalia with ductus bursae not coiled (Figs 16, 17, 19)	**5**
5	Male genitalia with uncus divergently bifurcate (Fig. 18A–D) or dramatically expanded apically (Fig. 18E–G); terminal plate of gnathos vertically bifurcate (Fig. 18A–C) or apically rounded (Fig. 18D–G); female genitalia with capitulum absent, signum reduced or absent (Fig. 19)	*** Claduncaria ***
–	Male genitalia with uncus variable, but never divergently bifurcate; terminal plate of gnathos acute, without modification (Figs 14, 15); female genitalia with signum, capitulum present (Figs 16, 17)	*** Argyrotaenia ***

#### Argyrotaenia

Taxon classificationAnimaliaLepidopteraTortricidae

Stephens, 1852

E5785D60-C88E-5B92-A05E-20E37FB58DDB

##### Type species.

*Tortrix politana* Haworth, [1811]

*Argyrothaenia* Diakonoff, 1939 [misspelling of *Argyrotaenia*]: 190.

*Subargyrotaenia* Obraztsov, 1961: 38.

##### Remark.

The following description is specific to Caribbean *Argyrotaenia*. However, most characters mentioned also apply to extralimital species.

##### Redescription.

Labial palpus 1.5–2.0 × width of compound eye, second segment expanded apically; ocellus small, separated from compound eye by approximately width of ocellus; chaetosemata 0.25–1.00 × length of scales on frons; metathorax without dorsal scaling, patch of pale yellow setae present; costal fold absent; FWL 4.5–10.5 mm. Male genitalia with uncus variable, usually spatulate or subquadrate, occasionally narrow and acute; socii obsolete; tegumen moderate; arms of gnathos fused; transtilla without modifications; valva circular to subcircular, occasionally trigonal or trapezoidal, longitudinal fold of valva well-developed (except in *A. ceramica*). Female genitalia with papillae anales triangular or nearly so (occasionally narrowly rectangular), flattened and evenly roughened on ventral surface; colliculum present; signum present, usually long and J-shaped; capitulum present, with variable basal plate.

### Key to species of Caribbean *Argyrotaenia*

**Table:** 

1	FW elongate, distinctly acute at apex, red-orange (Fig. 5A–E); male genitalia with plications obsolete, phallus sickle-shaped; cornuti long, thin; caulis large (Fig. 14A); female genitalia with capitulum large, roughened; signum not curved (Fig. 16A)	**2**
–	FW variable, but not as above; male genitalia with plications present, phallus pistol-shaped, cornuti variable, caulis minute; female genitalia with capitulum smooth, signum curved	**3**
2	Hispaniola	***A. ceramica ceramica***
–	Cuba	***A. ceramica granpiedrae***
3	FW with banding obsolete, straw yellow, with fine network of reticulations (Austin et al. 2019: fig. 2a–c); male genitalia with uncus broad, valva rectangular (Austin et al. 2019: fig. 3c, d, h); The Bahamas	***A. flavoreticulana***
–	FW variable, but not as above; male genitalia with uncus variable, valva circular or semicircular	**4**
4	FW with a distinct, dark L-shaped mark present along the medial half of the inner margin of the median fascia (Fig. 8A, B), often bordered with a white patch in females (Fig. 8A); male genitalia with presaccular gap occupying 0.5 × area of disc of valva (Fig. 14D); Hispaniola	***A. nuezana***
–	FWL without such a mark; presaccular gap variable, but never occupying 0.5 × width of disc of valva	**5**
5	FW chocolate brown and male genitalia with uncus without bulb, setae projecting laterally from neck (Fig. 14B, C)	**6**
–	FW and uncus variable, but never with the preceding combination of characters	**7**
6	Male genitalia with presaccular gap wide, occupying 0.33 × width of disc of valva, valva forming right angle at apex (Fig. 14C); female genitalia with capitulum rounded (Fig. 16C); Cuba, Hispaniola	***A. cubae***
–	Male genitalia with presaccular gap narrow, occupying no more than 0.15 × width of disc of valva; valva circular (Fig. 14B); female genitalia with capitulum truncate (Fig. 16B); Hispaniola	***A. browni* sp. nov.**
7	FWL large (8.5–9.5 mm), broad, pale brown, banding faint to obsolete (Fig. 8G, H); Hispaniola	***A. razowskiana* sp. nov.**
–	FWL variable, but not as above	**8**
8	Dorsal surface of hindwing without strigulae (Figs 5F–H; 6)	**9**
–	Dorsal surface of hindwing with strigulae (Figs 7, 9)	**12**
9	FW costa with distinct concavity at distal third (Fig. 5G, H); Jamaica	***A. jamaicana***
–	FW costa without such a distinct concavity at distal third	**10**
10	FW with median fascia distinctly bicolored (Austin et al. 2019: fig. 2d); male genitalia with uncus narrow, without developed bulb, setae projecting laterally from neck (Austin et al. 2019: fig. 3b); The Bahamas	***A. kimballi***
–	FW variable, but not as above; male genitalia with developed bulb of uncus, never with setae laterally projecting from neck	**11**
11	FW small (4.5–5.0 mm), entirely red-orange, banding faint to obsolete (Fig. 5F); Cuba	***A. vinalesiae***
–	FW size variable, pattern hypervariable, but never entirely red-orange, banding usually distinct (Fig. 6); widespread in northern Caribbean	***A. amatana***
12	Male genitalia with neck of uncus extremely narrow, no more than 0.25 × width of bulb (Fig. 14E); Hispaniola	***A. felisana***
–	Male genitalia with neck of uncus moderate, 0.5–1 × width of bulb	**13**
13	FW variable, but males usually with a distinct dark dot at the end of the discal cell in the postmedian interfascia (Fig. 7A, B); Hispaniola	*A. bisignata*
–	FW without such a dot, usually strongly mottled throughout (Fig. 9)	**14**
14	FW quadrate, male FW with fasciae chocolate brown, interfasciae strongly contrasting silver-gray to white (Fig. 9E, F); Hispaniola	***A. paradisei* sp. nov.**
–	FW elongate with apex acute, fasciae brick red (Fig. 9A–D); Hispaniola	**15**
15	Cordillera Central	***A. cryptica cryptica* ssp. nov.**
–	Sierra de Bahoruco	***A. cryptica praeteritana* ssp. nov.**

#### Argyrotaenia ceramica

Taxon classificationAnimaliaLepidopteraTortricidae

Razowski, 1999

48851B8F-C753-57B5-9676-445475FA30FB

**Figure 5.**
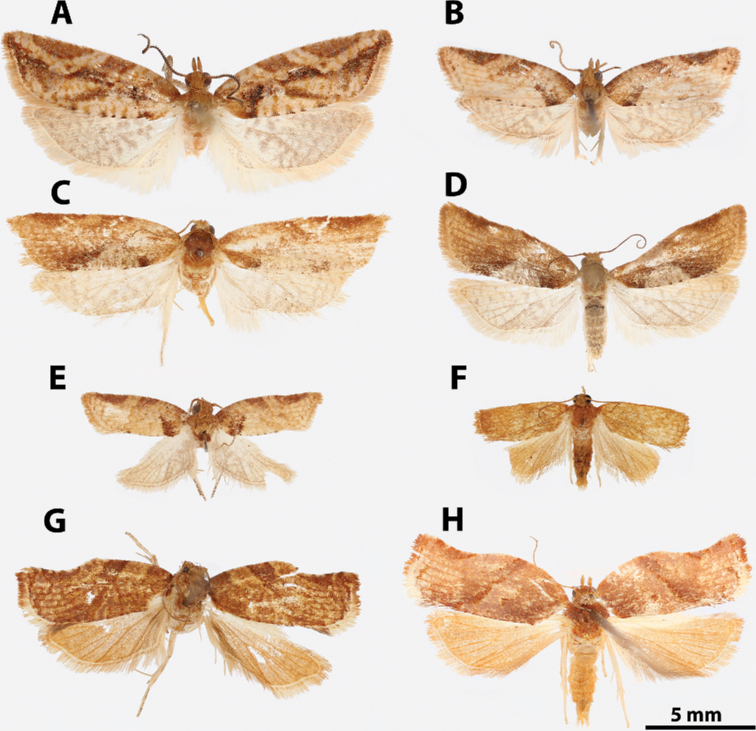
*Argyrotaenia* adults. **A***A. ceramica* holotype ♂, Dominican Republic (CMNH) **B***A. ceramica* paratype ♂, Dominican Republic (CMNH) **C***A. ceramica* paratype ♀, Haiti (CMNH) **D***A. ceramica* ♀, Dominican Republic (CUIC) **E***A. ceramica granpiedrae* stat. nov. Cuba (VBC) **F***A. vinalesiae* ♀, Cuba (VBC) **G***A. jamaicana* holotype ♂, Jamaica (CMNH) **H***A. jamaicana* ♀, Jamaica (CUIC).

Figs 5A–E , 14A , 16A , 24A

##### Diagnosis.

*Argyrotaenia ceramica* (Fig. 5A–E) closely resembles members of the *A. ponera* group (Brown and Cramer 1999) in having both an unusually elongate wing shape in comparison to its congeners and in male genitalia with a strongly curved phallus and a well-developed caulis. *Argyrotaenia ceramica* can be separated by its deeply notched juxta and relatively broader phallus (Fig. 14A). The female genitalia (Fig. 16A) are not likely to be confused with any known Caribbean *Argyrotaenia*. The signum is short (approximately 0.33 × width of corpus bursae) and straight with a roughened, irregular capitulum. Small males could be confused with males of *Clepsis jamesstewarti* (Fig. 12D), but the genitalia are distinct.

##### Remarks.

We found no morphological differences between the holotypes of *A. ceramica* Razowski, 1999 and *A. granpiedrae* Razowski & Becker, 2010. An incomplete COI barcode (408 bp) was recovered for a Cuban specimen. When complete Hispaniola sequences were aligned and cut to the same length, significant sequence divergence was observed (7.0–7.3%). We relegate *A. granpiedrae* to a subspecies of *A. ceramica* because of the lack of morphological differences, yet choose not to synonymize it based on observed differences in COI sequences (albeit incomplete), hoping that this will spur future work. Based on forewing pattern and male genitalia, *A. ceramica* appears to belong to the *ponera* group of species (Brown and Cramer 1999) from central Mexico and the southwestern United States.

#### Argyrotaenia ceramica ceramica

Taxon classificationAnimaliaLepidopteraTortricidae

Razowski, 1999

13AE7EB8-27E9-504E-BF95-5CD7635CD373

Figs 5A–D , 14A , 16A , 24A

Argyrotaenia ceramica Razowski, 1999: 309

##### Diagnosis.

*Argyrotaenia c. ceramica* (Fig. 5A–D; Hispaniola) is morphologically indistinguishable from *A. c. granpiedrae* (Fig. 5E; Cuba), but differs in COI barcode (see remarks under species account).

##### Type material.

***Holotype*** ♂: **Dominican Republic: Pedernales**: 8 km NE Los Arroyos, 1940 m, 18°16'N, 71°44'W, 14 vii 1990, J. Rawlins, C.W. Young, S.A. Thompson [examined]. Razowski genitalia slide #10705 [examined] (CMNH). ***Paratypes*** 4♂♂, 2♀♀): **Dominican Republic: Pedernales**: 1♂, La Abeja, 38 km NNW Cabo Rojo, 18°09'N, 71°38'W, 1160 m, 14 vii 1987, J.E. Rawlins, R.L. Davidson [examined]. Razowski genitalia slide #10706 [not examined] (CMNH). 1♂, 5 km NE Los Arroyos, 18°15'N, 71°45'W, 1680 m, 15–16 vii 1990, C. Young, J.E. Rawlins, S. Thompson [photo examined] (ISEZ). **Peravia [San José de Ocoa**]: 1♂, 3 km SW La Nuez, tributary to Rio Las Cuevas, 18°40'N, 70°36'W, 1870 m, 5–6 viii 1990. J. Rawlins, S. Thomson [examined]. Razowski genitalia slide #10707 [examined] (CMNH). **Haiti: Ouest**: 1♂, 2♀♀, Kenskoff [Kenscoff], 3 v 1937, Roys, 4300' [examined]. Razowski genitalia slide #10708 (♀) [examined], #10709 (♂/♀) [not examined], #10710 (♂/♀) [not examined] (CMNH).

##### Additional material examined.

(30♂♂, 24♀♀). **Dominican Republic: Independencia**: 1♀, Sierra de Bahoruco, north slope, 2116 m, broadleaf forest with pine, 18°41'31"N, 71°35'35"W [18°17'30"N, 71°43'08"W], 8 xi 2002, W.A. Zanol, C.W. Young, C. Staresinic, J. Rawlins (CMNH). 2♂♂, 5♀♀, 3 km ESE El Aguacate, north slope Sierra de Ba[h]oruco, 1980 m, pine woodland, 18°18'N, 71°42'W, 28–29 ix 1991, J. Rawlins, R. Davidson, C. Young, S. Thompson (1♂ CUIC, remainder CMNH). KAA diss. #0085(♀); #0087(♂), KAA_DNA_0009 (CMNH). 1♂, Sierra de Neiba, near crest, 5.5 km NNW Angel Feliz, 1750 m, dense cloud forest, 18°41'N, 71°47'W, 21–22 vii 1992, J. Rawlins, S. Thompson, C. Young, R. Davidson, KAA diss. #0088 (CMNH). 1♂, Sierra de Bahoruco, north slope, 13.3 km SE Puerto Escondido, 1812 m, 18°12'33"N, 71°30'47"W, 24–26 iii 2004, *Pinus*, *Rubus*, *Garrya*, open, R. Davidson, J. Rawlins, C. Young, C. Nunez, M. Rial (CMNH). 1♂, Sierra de Bahoruco, north slope, 13.5 km SE Puerto Escondido, 1807 m, broadleaf *Pinus* dense woodland, 18°12'24"N, 71°30'54"W, 24–26 iii 2004, R. Davidson, J. Rawlins, C. Young, C. Nunez, M. Rial (CMNH). 3♂♂, 2♀♀, Sierra de Bahoruco, north slope, 13.5 km SE Puerto Escondido, 1789 m, ecotonal *Pinus* grassland 18°12'18"N, 71°31'08"W, 24–25 xi 2004, J.E. Rawlins, C. Young, C. Nunez, V. Verdecia, W.A. Zanol, KAA diss. #0089 (CMNH). 1♂, 3♀♀, Sierra de Neiba, just south of crest, 5 km WNW Angel Feliz, 1780 m, cloud forest 18°41'N, 71°47'W, 13–15 x 1991, J. Rawlins, R. Davidson, C. Young, S. Thompson (CMNH), Razowski genitalia slide #10734 (♀) [examined], #10735 (♀) [examined], #10736(♀) [examined] (CMNH). 1♀, Sierra de Neiba, south slope near summit, 4.0 km N Angel Feliz, broadleaf cloud forest without pine, 1825 m 18°40'21"N, 71°46'05"W, 1–2 iv 2004, J. Rawlins, C. Young, R. Davidson, KAA_DNA_0013 (CMNH). **La Estrelleta [Elías Piña**]: 1♀, 4 km SE Rio Limpio, c. 760 m, 24–25 v 1973, Don Davis, Mignon Davis (USNM). 1♂, 1♀, Sierra de Neiba at crest, 5.5 km WNW N Angel Feliz, 1800 m, cloud forest, 18°41'N, 71°47'W, 15 x 1991, R. Davidson, C. Young, S. Thompson, J. Rawlins (♂ CMNH, ♀ CUIC). **La Vega**: 2♂♂, 2.5 km SW Pinar Bonito, 1430 m, riparian vegetation near stream in pine woodland 18°51'N, 70°43'W, 26 xi 1992, J. Rawlins, R. Davidson, M. Klingler, S. Thompson (CMNH). 2♂♂, 4.1 km SW El Convento, 1710 m, secondary broadleaf forest, 18°50'37"N, 70°42'48"W, 14 xi 2002, W.A. Zanol, C.W. Young, C. Staresinic, J. Rawlins, KAA diss. #0092 (CMNH). 1♀, Constanza, 2–6 vi 1969, Flint & Gomez, KAA diss. #0084 (USNM). 1♂, 5♀♀, Convento, 12 km S of Constanza, 6–13 vi, 1969, Flint & Gomez (USNM). **Pedernales**: 11♂♂, 1 km S Los Arroyos, 1125 m, second growth forest, 18°14'N, 71°45'W, 18 x 1991, R. Davidson, C. Young, S. Thompson, J. Rawlins, KAA diss. #0090 (CMNH). 1♀, 26 km N Cabo Rojo, 730 m, mesic deciduous forest with scattered pines, 18°06'N, 71°38'W, 16 vii 1992, C. Young, R. Davidson, S. Thompson, J. Rawlins, KAA diss. #0086 (CMNH). 1♂, 5 km NE Los Arroyos, 1680 m, cloud forest, 18°15'N, 71°45'W, 30 ix 1991, R. Davidson, C. Young, S. Thompson, J. Rawlins (CMNH). **Peravia [San José de Ocoa**]: 2♂♂, 3♀♀, 3 km SW La Nuez, upper Rio Las Cuevas, 1880 m, cloud forest on river, 18°39'N, 70°36'W, 5–6 x 1991, J. Rawlins, R. Davidson, C. Young, S. Thompson (1♂, 1♀ CUIC; remainder CMNH). KAA diss. #0083(♀), KAA_DNA_0012 (CMNH). **Haiti: Sud**: 1♂, Ville Formon, 31 km NW Les Cayes, S slope Morne Formon, Massif de La Hotte, 1405 m, disturbed forest and fields, 18°20'N, 74°01'W, 7–8 ix 1995, R. Davidson, G. Onore, J. Rawlins, KAA diss. #0091, KAA_DNA_0010 (CMNH).

##### Redescription.

**Male (n = 34). *Head*.** Typical of genus. Scales on vertex ochraceous-orange to maize yellow. Frons similarly colored. Labial palpus with scales on lateral surface of first and second segment bicolored, with basal half pale yellow and apical half ochraceous-orange, occasionally a few scales entirely black. Terminal segment similar in coloration, but with more prominent black scaling. Medial surface of palpus pale yellow. Scape concolorous with vertex; sensillae approximately 0.75 × width of flagellomere; dorsal scales of flagellum dark brown, occasionally pale yellow; second row of scales on each flagellomere expanded noticeably, giving appearance of thickened antennae. ***Thorax*.** Typical of genus. Dorsum of pro- and meso-thorax concolorous with vertex; tegulae also concolorous. Forelegs predominantly dark brown intermixed with pale yellow scales; femur with ochraceous-orange scales as well. Midlegs similar to forelegs but without ochraceous-orange scaling on femur, tarsi pale yellow to dark brown. Hindlegs pale yellow to white. Medial surface of legs pale yellow to white. Forewing pattern with two distinct forms, FWL 5.0–8.5 mm (mean = 6.3; n = 34); costa with basal quarter evenly curved, straight beyond. One form (Fig. 5B) more common, significantly smaller (mean = 6.2 mm; n = 31, including three paratypes), with dorsal surface of forewing with ground color pale yellow; basal fascia, median facia, and subapical blotch amber brown intermixed with dark brown scales; amber brown dots along inner margin; tornal blotch obsolete. Second form (Fig. 5A) less common, significantly larger (n = 3, including holotype; mean FWL = 8.0 mm), with a crimson red streak through wing from base to near apex; black scales are sometimes present in portions of streak. Fringe pale orange-yellow, brick red and dark gray scales present at apex in most specimens. Tornal blotch present. Dorsal surface of hindwing white to pale yellow, with light brown mottling throughout, becoming more densely mottled apically in some individuals. Fringe composed of long pale red-orange scales, becoming off-white along posterior third; shorter pale brown scales present along margin in some specimens (Fig. 5B) Ventral surface of forewing light brown basally, pale yellow near apex. Ventral surface of hindwing similar to dorsal surface. ***Abdomen*.** Vestiture warm brown, terminal segment pale yellow. Genitalia (Fig. 14A) with uncus uniform in width, unmodified, tapered at apex; arms of gnathos unmodified, evenly curved; tegumen unmodified; transtilla thin, complete, unmodified; valvae nearly triangular with long setae scattered at margins; presaccular gap and longitudinal fold obsolete; sacculus apparent at base to 0.5 × length of valvae, marginal beyond; plications obsolete; dense cluster of apically-widened, brush-like setae present at base of valvae; juxta deeply notched; phallus strongly curved, caulis prominent, well-developed; two to four cornuti present, approximately 0.8 × length of phallus, thin, nearly straight, deciduous. A cluster of five cornuti present observed in the corpus bursae of one dissected female (Fig. 16A).

**Female (n = 26). *Head*.** As in male except antennae with sensillae minute, approximately 0.25 × width of flagellomere, second row of scales on each flagellomere not expanded as in male. ***Thorax*.** As in male in coloring on legs and thorax. Forewing (Fig. 5C, D) with FWL 6.0–8.5 mm (mean = 7.3; n = 26). Dorsal surface of forewing ochraceous-orange to chocolate brown; markings as in male but with markings less well-defined and much less contrasting, except for a distinct patch of white scales halfway along inner margin. Frenulum with two or three bristles, asymmetrical in number in several specimens examined. ***Abdomen*.** Vestiture as in male. Genitalia (Fig. 16A) with papillae anales elongate, narrow, slightly curved laterally; apophyses posteriores approximately 0.5 × length of sternum VII; apophyses anteriores approximately 0.67 × length of sternum VII; sterigma broad, evenly curved; ductus bursa approximately 2 × length of sternum VII, broadening anteriorly; ductus seminalis arising at approximately 0.25 × length of ductus bursae; corpus bursa round; signum thin, straight, 0.25–0.50 × length of corpus bursae; capitulum of signum rounded to irregular, strongly roughened.

##### Distribution.

*Argyrotaenia ceramica ceramica* is widespread at mid- and high elevations (700–2200 m) on Hispaniola (Fig. 24A).

##### Ecology.

Nothing is known of the biology of *A. c. ceramica*. However, due to the highly variable size of males, we hypothesize it may be an internal feeder. Collection dates range from April to November.

##### Remarks.

There is a discrepancy in the label data of one female specimen from Independencia. The label reads “Sierra de Bahoruco” but the coordinates are for the Sierra de Neiba. After comparing coordinates from specimens collected the previous night and discussing the situation with John Rawlins (CMNH), we interpret the coordinates to be incorrect. Dr. Rawlins kindly supplied us with the correct coordinates. COI sequence divergence among barcoded specimens of *A. c. ceramica* was between 0.1% and 1.7% (n = 4).

#### Argyrotaenia ceramica granpiedrae

Taxon classificationAnimaliaLepidopteraTortricidae

Razowski & Becker, 2010 stat. nov.

A143DB8D-4E4F-50A4-877A-49ED1CF5DD5C

Fig. 5E , 24A; Razowski & Becker, 2010: figs 19, 20, 46, 71

Argyrotaenia granpiedrae Razowski & Becker, 2010: 17

##### Diagnosis.

See the diagnosis under *A. c. ceramica*.

##### Type material.

***Holotype*** ♂: **Cuba: S[an]t[ia]go [de Cuba**]: Gran Piedra, 20 vi[i] 1990, V.O. Becker; Col. Becker 72991 [photograph examined]. Genitalia slide #409 [figure examined] (VBC, see remarks below). ***Paratypes*** (3♂♂, 1♀): same data as holotype [female genitalia figure examined] (VBC, see remarks below).

##### Additional material examined.

(1♂, 2♀♀). **Cuba**: **S[an]t[ia]go [de Cuba**]: 1♂, Gran Piedra, same data as holotype (VBC). KAA diss. #0161, KAA_DNA_0011 (VBC). 2♀♀, Sierra Maestra, Pico Cuba, 31 vii 1990, V. O. Becker [photographs examined] (ISEZ).

##### Redescription.

**Male (n = 1). *Head***. Identical to *A. c. ceramica*. ***Thorax*.** Wing pattern identical to the more common form of *A. c. ceramica*. FWL 5.0 mm. Though smaller than the two specimens pictured in Fig. 5C, D, the specimen pictured in Fig. 5E is well within the size range observed in other *A. c. ceramica*. ***Abdomen*.** Identical to *A. c. ceramica*, including genitalia (see Razowski & Becker, 2010: figs 19, 20).

**Female (n = 0).** No specimens were examined, only photographs (see Razowski & Becker, 2010: fig. 46).

##### Distribution.

This subspecies is known from two high-elevation localities in southern Cuba (Fig. 24A).

##### Ecology.

Nothing is known of the biology of *A. c. granpiedrae*. All examined specimens were collected in July.

##### Remarks.

See the remarks under the species account of *A. ceramica* for why we consider *A. granpiedrae* to be a subspecies of *A. ceramica*. The holotype of *A. granpiedrae* and the female paratype are deposited in ISEZ, not in VBC as listed in Razowski and Becker (2010). The remaining male paratypes are likely in ISEZ as well. Two non-type females were also found in ISEZ that had been identified by Razowski as *A. ceramica*. It is unclear whether these were identified before or after *A. granpiedrae* was described. Razowski listed the holotype as having been collected in June, but we suspect the label was erroneously transcribed, as we examined a specimen with otherwise identical labels and accession numbers with the month of “vii” not “vi.”

#### Argyrotaenia vinalesiae

Taxon classificationAnimaliaLepidopteraTortricidae

Razowski & Becker, 2010

EEE97890-E5F2-5AB7-8B0C-EB70715521F4

Figs 5F , 17B , 23 ; Razowski and Becker 2010: figs 9, 10, 61

Argyrotaenia vinalesiae Razowski & Becker, 2010: 13

##### Diagnosis.

**Figure 6.**
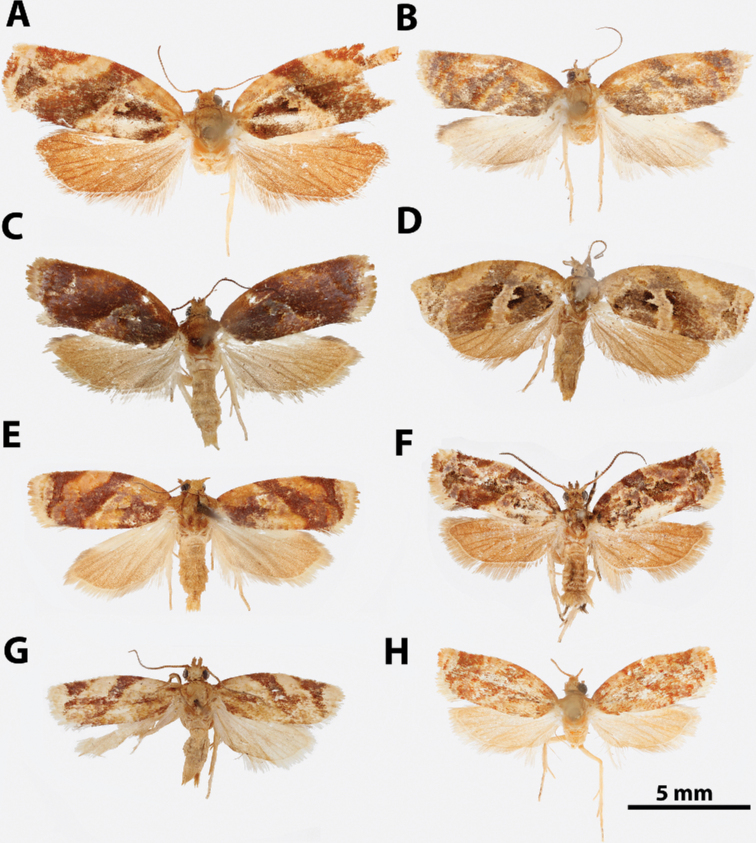
*Argyrotaenia amatana* adults. **A***A. amatana* ♀ (holotype of *A. neibana* syn. nov.), Dominican Republic (CMNH) **B***A. amatana* ♀ (holotype of *A. ochrochroa* syn. nov.), Turks & Caicos (CMNH) **C***A. amatana* ♀, The Bahamas (MEM) **D***A. amatana* ♀, Cuba (USNM) **E***A. amatana* ♂, Florida (CUIC) **F***A. amatana* ♂, Cuba (USNM) **G***A. amatana* ♂, Grand Cayman (BMNH) **H***A. amatana* ♂, Dominican Republic (CMNH).

*Argyrotaenia vinalesiae* (Fig. 5F) is most similar to *A. amatana* (Fig. 6), a widespread northern Caribbean species. It differs by its smaller size (4.5–5.0 mm in females), uniformly-colored forewing, and shorter, broader signum in the female genitalia (Fig. 17B) compared to *A. amatana* (Austin et al. 2019: fig. 4a). Male genitalia are indistinguishable from those of *A. amatana*.

##### Type material.

***Holotype*** ♂ [see remarks below]: **Cuba**: **Pinar del Río**: Viñales, 100 m, 20 viii 1990, V. O. BECKER Col; Col. BECKER 73817 [photograph examined] (VBC, see remarks below). Genitalia slide #404 [figure examined]. ***Paratype*** (♀): same data as holotype [photograph examined]. Genitalia slide #405 [figured examined] (VBC, see remarks below).

##### Additional material examined.

(2♀♀). **Cuba: Pinar del Río**: 2♀♀, same data as holotype (VBC). KAA diss. #0159; #0160, KAA_DNA_0034 (VBC).

##### Redescription.

**Male.** We were unable to examine any male specimens, so our redescription here is based on photographs of specimens in ISEZ and VBC and the figures available in Razowski and Becker (2010). ***Head*.** See Razowski and Becker (2010). ***Thorax*.** Scaling on dorsum of pro- and meso-thorax slightly darker than examined females. Forewing with basal quarter of costa gently curved, straight beyond; dorsal surface of forewing darker than female with more distinct banding: basal fascia and median fascia red-orange, fringe warm orange; dorsal surface of hindwing similar to female; ventral surface of wings unexamined. ***Abdomen*.** Vestiture similar to female. Genitalia (Razowski and Becker 2010: fig. 9) with uncus uniformly broad throughout, quadrate at apex; arms of gnathos moderate, unmodified, evenly curved; transtilla complete, narrowest mesad; valvae broad, circular; sacculus to 0.33×; presaccular gap moderate, uniform in width; juxta hexagonal with moderate notch, small setae present laterally; phallus (Razowski and Becker 2010: fig. 10) pistol-shaped, gently curved, caulis moderate; deciduous cornuti present (five observed in corpus bursae of one examined female), moderate in size, slightly undulate.

**Female (n = 2). *Head*.** Typical of genus. Scales on vertex, frons, and labial palpus golden yellow to straw yellow. Scape with scales similarly colored; sensillae approximately 0.5 × width of flagellomere; scales on flagellomeres bicolored, alternating between a golden yellow apical row and a caramel brown basal row. ***Thorax*.** Typical of genus. Scales on dorsum of pro- and meso-thorax golden yellow; tegulae concolorous. Scaling on lateral surface of foreleg straw yellow, tarsi warm brown; scaling on midleg and hindleg pale yellow; medial surface of all legs with pale yellow scaling. Forewing (Fig. 5F) with basal quarter of costa gently curved, straight beyond; FWL 4.5–5.0 mm (mean = 4.8; n = 2); dorsal surface uniformly warm orange-yellow to golden yellow, fringe concolorous; dorsal surface of hindwing orange-yellow, strigulae absent, fringe concolorous; ventral surface of both wings similar to ventral surface but slightly paler. ***Abdomen*.** Vestiture concolorous with dorsal surface of hindwing, slightly darker terminally. Genitalia (Fig. 17B) with papillae anales triangular; apophyses posteriores approximately 0.5 × length of sternum VII; apophyses anteriores approximately 0.67 × length of sternum VII; sterigma lightly sclerotized, thin, broadly bowl-shaped; ductus bursae widening gradually anteriorly; ductus seminalis arising at approximately 0.2 × length of ductus bursae; corpus bursae large, circular; signum moderate, moderately hooked at apex; capitulum moderately acute, opposite-facing.

##### Distribution.

*Argyrotaenia vinalesiae* is known from a series of specimens taken on a single night in Viñales, Cuba at an elevation of 100 m (Fig. 23).

##### Ecology.

Nothing is known of the biology of *A. vinalesiae*. The short series of this species was collected in August.

##### Remarks.

The holotype of *A. vinalesiae* is listed as a female in the original description, but the male adult and its genitalia illustrated are listed as the holotype. Both the holotype and paratype were found in ISEZ, not VBC as listed in Razowski and Becker (2010). The male specimen in ISEZ has a red holotype label, so we interpret the “female” in the description to be an error and the holotype to be male.

We were unable to find significant differences in male genitalia of *A. vinalesiae* and *A. amatana*. Despite their sympatry in western Cuba, differences in size and forewing pattern, as well as COI sequence divergences, support treating the two as distinct species (see remarks under *A. amatana* and *A. jamaicana* regarding these three species’ relationships).

#### Argyrotaenia jamaicana

Taxon classificationAnimaliaLepidopteraTortricidae

Razowski & Becker, 2000b

1E84ECC9-65B7-5F45-9C5D-383E4AF1E7A2

Figs 5G, H , 15B , 17A , 23

Argyrotaenia jamaicana Razowski & Becker, 2000b: 313

##### Diagnosis.

**Figure 7.**
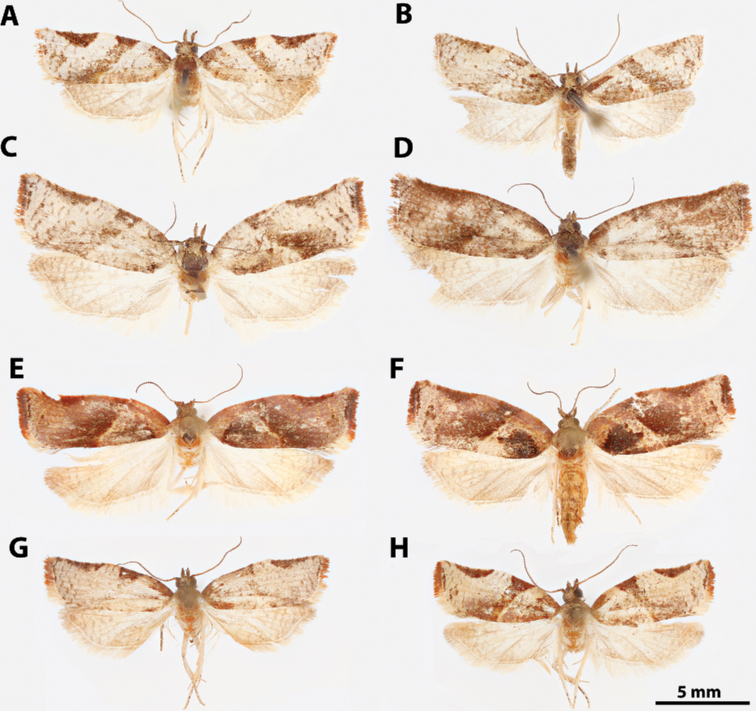
*Argyrotaenia* adults. **A***A. bisignata* holotype ♂, Dominican Republic (CMNH) **B***A. bisignata*, ♂ Dominican Republic (CMNH) **C***A. bisignata* paratype ♀, Dominican Republic **D***A. bisignata* paratype ♀, Dominican Republic (CMNH) **E***A. felisana* holotype ♀, Dominican Republic (CMNH) **F***A. felisana* ♀, Dominican Republic (CUIC) **G***A. felisana* ♂, Dominican Republic (CUIC) **H***A. felisana* ♂, Dominican Republic (CUIC).

*Argyrotaenia jamaicana* is strongly sexually dimorphic. Worn males could be confused with *A. amatana* because of their diminutive size, but the strongly concave costa at the distal third of the forewing of *A. jamaicana* (Fig. 5G) should easily separate them from males of *A. amatana* (Fig. 6E–H). Females are also similar to those of *A. amatana*, but also possess a strongly concave costa at the apical third of the forewing and have a less strongly contrasting forewing pattern (Fig. 5H) compared to females of *A. amatana* (Fig. 6A–D). Females could also be confused with females of *A. felisana* (Fig. 7E, F) from Hispaniola, another sexually dimorphic species, especially because some females of *A. felisana* also possess a strongly concave forewing costa (Fig. 7E). However, *A. jamaicana* females have a more orange overall hue in both the forewing and hindwing (Fig. 5H). Male genitalia of *A. jamaicana* (Fig. 15B) are likely to be confused with *A. amatana*, *A. bisignata*, and *A. razowskiana*. From *A. amatana* (Austin et al. 2019: fig. 3a), *A. jamaicana* differs in having a narrower uncus and longer terminal plate of the gnathos. From *A. bisignata* (Fig. 15A) it differs in having a much longer, thinner terminal plate of the gnathos and more curved phallus. From *A. razowskiana* (Fig. 15C) it differs in having a broader neck of the uncus and a more curved phallus. The female genitalia of *A. jamaicana* (Fig. 17A) most closely resemble those of the same three species, but can be separated by having smaller, less elongate papillae anales and a more evenly rounded capitulum. Both *A. jamaicana* and *A. amatana* occur on Jamaica, but they appear to be allopatric, with *A. jamaicana* restricted to mid- and high elevations and *A. amatana* to the immediate coast (Fig. 23).

##### Type material.

***Holotype*** ♂: **Jamaica**: Greenhills, Hardwar Gap, 27 iii 1936, E. Paine [examined]. Razowski genitalia slide #12274 [examined] (CMNH).

##### Additional material examined.

(17♂♂, 8♀♀). **Jamaica**: **Portland**: 2♂♂, 1♀, Green Hills, 11 iii [19]66, S.S. Duckworth, W.D. Duckworth (1♂ CUIC, remainder USNM). KAA diss. #0128(♀), (USNM). 3♂♂, Hardwar Gap, “Green Hills”, 16–17 vii 1963, Flint & Farr. One with JAP diss. #3182, USNMdiss. ##68325 [examined] (USNM). 1♂, 1♀, 1 mi N Hardwar Gap, 12–20 xi 1966, E.L. Todd (♂ CUIC, ♀ USNM). KAA diss. #0127(♀) (USNM). **St. Andrew**: 1♀, Newcastle, Rothschild Bequest, B.M. 1939-1 (BMNH). 1♂, Newcastle, str. at mile 16.5, 30 vii 1962, O. Farr, R. Flint (USNM). 2♂♂, same as previous, but 18 vii 1963 (CUIC, USNM). 1♂, Chestervale, Yallahs River, 24–25 vii 1962, O. Farr, R. Flint, KAA diss. #0131 (USNM). 1♂, same as previous, but 17 vii 1963 (USNM). 1♂, Hermitage Dam, 22–23 vii 1962, O. Farr, R. Flint (USNM). **St. Ann**: 1♂, Moneague, *Parthenium hysterophorus* ex. 23 ii 1905, Wlsm, 77032. Walsingham Collection, 1910–427. [*Tortrix partheniana* type ♂]. **St. Catherine**: 4♂♂, 4♀♀, Mt. Diablo, Hollymount, 2754 ft., 21–24 iv [19]73, Don Davis, Mignon Davis (2♀♀ CUIC; remainder USNM, including 1♂ USNMENT01480198 and 1♀ USNMENT01480208). KAA diss. #0129 (♀) (USNM). 1♀, Worthy Park, 2.2 mi N on Camperdown Road, R.E. Woodruff, 18–25 ii [19]70, malaise trap (USNM).

##### Redescription.

**Male (n = 17). *Head*.** Typical of genus. Scales on vertex straw yellow intermixed with a few light red-orange scales. Frons with scaling red-orange. Labial palpus with scales on lateral surface dull red-orange, with scattered straw yellow and brick red scales; medial surface pale yellow. Scape pale yellow to straw yellow; sensillae approximately same width as flagellomere, recurved, but not as strongly as in other Caribbean *Argyrotaenia*; dorsal scales of flagellum alternating between a basal row of mahogany red and apical row of red-orange scales. ***Thorax*.** Typical of genus. Dorsum of pro-and meso-thorax pale yellow to red-orange; tegulae concolorous, slightly darker in some specimens. Lateral surface of forelegs warm brown to dark brown, lateral surface of midlegs and hindlegs straw yellow to white, tarsi and tibial spurs occasionally warm brown. Medial surface of legs white. Forewing (Fig. 5G) costa with a conspicuous concavity at distal third, FWL 5.0–7.0 mm (mean = 5.9; n = 17). Scaling on dorsal surface of forewing with antemedian and postmedian interfasciae light yellow, strongly mottled with orange and ochraceous red throughout, banding obsolete in some specimens, well-developed in others; basal fascia, median fascia, and subapical blotch variable, sometimes nearly obsolete, visible only as faint brick red along costa, in other specimens jet black with a wash of blue-gray scales; fringe with apical half dark red-orange near apex, basal scales of fringe replaced with pale yellow scales towards tornus. Dorsal surface of hindwing golden orange; short fringe scales concolorous, longer scales pale yellow to off-white. Ventral surface of forewing orange, with white and ochraceous red markings along costa. Ventral surface of hindwing as on dorsal surface. ***Abdomen*.** Vestiture with scaling concolorous with hindwing, almost gold. Genitalia (Fig. 15B) with uncus moderate in width, narrowest at midpoint, slightly bulbous in distal third, apicoventral setae sparse, short; arms of gnathos unmodified, evenly curved, but with dorsal ridge giving appearance of it being strongly bent; tegumen moderate; transtilla thick, U-shaped; valva broadly circular; sacculus apparent at base to 0.5 × of valva, narrow beyond; dense cluster of slender deciduous setae at base of valva; presaccular gap relatively narrow; juxta hexagonal, shallowly notched; phallus evenly curved, caulis minute; approximately twelve cornuti in holotype, approximately 0.33 × length of phallus, thin, curved, deciduous.

##### Description.

**Female (n = 8). *Head*.** As in male except with extensive ochraceous red scaling on vertex, frons, and scape; lateral surface of labial palpus dull red-orange, with scattered brick red scales; sensillae short, porrect, no more than 0.5 × width of flagellum. ***Thorax*.** As in male but dorsum of pro- and meso-thorax with more extensive mahogany red scaling. Forewing with slightly more pronounced concavity along distal third of costa at subapical blotch; FWL 6.5–9.5 mm (mean = 7.6; n = 8). Dorsal surface of forewing (Fig. 5H) with banding more apparent than in male, mottling absent, basal fascia, median fascia, and subapical blotch mahogany red, but overlaid with purplish scaling, which is most noticeable under magnification. Submedian and subterminal interfascia straw yellow, but similarly overlaid with purplish scaling, obscuring most yellow scales. Fringe with apical lighter than male, apical half salmon pink, occasional brick red scales present. Dorsal surface of hindwing with fringe entirely concolorous with hindwing. ***Abdomen*.** Vestiture golden orange to warm brown. Genitalia (Fig. 17A) with papillae anales triangular, rounded laterally; apophyses posteriores approximately 0.5 × length of sternum VII; apophyses anteriores 0.75–1.0 × length of sternum VII; sterigma well-sclerotized, broadly bowl-shaped; ductus bursae widening gradually anteriorly; ductus seminalis arising at approximately 0.2 × length of ductus bursae; corpus bursae large, ovoid; signum moderate in width, long, J-shaped; capitulum of signum prominent, evenly rounded.

##### Distribution.

*Argyrotaenia jamaicana* is known exclusively from Jamaica (Fig. 23) at mid- to high elevations (350–1230 m). It appears to be replaced by *A. amatana* on the immediate coast.

##### Ecology.

One male from BMNH was reared from *Parthenium hysterophorus* L. (Asteraceae). It is likely a generalist. Capture dates range from February to November, suggesting several generations per year.

##### Remarks.

The above represents the first description of the female of *A. jamaicana*.

The holotype of *Argyrotaenia minisignaria chalarostium* was erroneously labeled as a female paratype of *A. jamaicana*. See the remarks under *Claduncaria chalarostium* for a full explanation. One male from BMNH is labeled as “*Tortrix partheniana* type ♂.” We can find no published record of this name and treat it as an unavailable manuscript name.

One male and one female were barcoded, but a sequence of > 500 bp was only recovered for the female, so we are unable to discuss sequence divergence within this species. See the remarks under *A. amatana* regarding this species’ relationship to it. COI sequence divergence between *A. jamaicana* (n = 1) and *A. vinalesiae* (n = 1) was 3.2%.

#### Argyrotaenia amatana

Taxon classificationAnimaliaLepidopteraTortricidae

(Dyar, 1901)

A0EE69B8-E140-5C98-96EF-71FC91BA512F

Figs 6 , 23 ; Austin et al. 2019: figs 1; 3a, f; 4a

Lophoderus amatana Dyar, 1901: 24
Eulia amatana (Dyar, 1901): Fernald [1903]: 485.
Tortrix chioccana Kearfott, 1907: 72
Argyrotoxa chiococcana Meyrick, 1912: 52; unjustified emendation
Argyrotaenia neibana Razowski, 1999: 310, syn. nov.
Argyrotaenia ochrochroa Razowski, 1999: 310, syn. nov.
Argyrotaenia ochrotona , misspelling in Razowski & Becker, 2000b: 312

##### Diagnosis.

*Argyrotaenia amatana* is a highly variable species, making it difficult to diagnose externally. Specimens from the same populations can vary dramatically in coloration, size, and maculation. It is most likely to be confused with *A. jamaicana* and *A. vinalesiae*, its two closest Caribbean relatives based on COI sequence data (Figs 3, 4). *Argyrotaenia amatana* (Fig. 6) differs from *A. vinalesiae* (Fig. 5F) in having a distinctly banded and slightly larger forewing, and in having a thinner, more curved signum in the female genitalia (Austin et al. 2019: fig. 4a) compared to *A. vinalesiae* (Fig. 17B); the male genitalia of the two are indistinguishable. It differs from *A. jamaicana* (Fig. 5G, H) in lacking a distinct concavity along the distal third of the forewing costa (Fig. 6) and having a wider presaccular gap in the male genitalia (Austin et al. 2019: fig. 3a) compared to *A. jamaicana* (Fig. 15B); the female genitalia of the two are indistinguishable.

##### Type material.

***Lophoderus amatana***: ***Syntypes*** 3♀♀: **USA**: Florida: Palm Beach Co., Palm Beach, r.f. *Nectandra* [=*Ocotea*] [photos examined] (USNM). ***Argyrotaenia neibana***: ***Holotype*** ♀: **Dominican Republic**: Ba[h]oruco: Sierra de Neiba, Los Guineos on upper Rio Colorado, 18°35'N, 71°11'W, 630 m, 11–12 viii 1990, mesic riparian woodland, J. Rawlins, S. Thompson [examined]. Razowski genitalia slide #1698 [examined] (CMNH). ***Argyrotaenia ochrochroa***: ***Holotype*** ♀: TURKS & CAICOS: Providenciales: Erebus Hotel area, ca. 21°48'N, 72°15'W, 28–30 i 1978, at hotel lights, H. Clench, M. Clench [examined]. Razowski genitalia slide #10695 [examined] (CMNH).

##### Additional material examined.

(60♂♂, 33♀♀). **The Bahamas: Cat Island**: 1♂, vic. Ocean Dream Resort, E of Smith Town, 24.232273, -75.454536, 23 vi 2014, J. Miller, M. Simon, D. Matthews, G. Goss, Bahamas Survey MGCL Accession No. 2014-15, MGCL 238585 (MGCL). 1♀, same as previous, but MGCL 238590 (MGCL). 1♂, same as previous, but MGCL 238601 (MGCL). **Crooked Island**: 1♂, 1.5 mi. E of Landrail Pt., 22.813263, -74.321186, 10 vi 2013, M. Simon, G. Goss, M. Simon MGCL Accession No. 2013-21, MGCL 233031, KAA diss. #0001 (MGCL). 1♀, same as previous, but 6 vi 2013, M. Simon & G. Goss, MGCL 234816 (MGCL). 1♀, same as previous, but MGCL 232998 (MGCL). 1♀, Pittstown Point, 22.831211, -74.438717, 9 vi 2013, M. Simon, G. Goss, M. Simon MGCL Accession No. 2013-21, MGCL 232999 (MGCL). 1♀, N side of Horseshoe Beach nr. Gun Bluff, 22.835432, -74.323017, 6 vi 2013, M. Simon, G. Goss, M. Simon MGCL Accession No. 2013-21, MGCL 232997 (MGCL). 1♂, 0.5 mi. E of Ferry at Church Grove Settlement, 22.758933, -74.242501, 6 vi 2014, M. Simon & M. Simon, Bahamas Survey MGCL Accession No. 2014-13, MGCL 236778 (MGCL). **Eleuthera**: 1♂, N of Queen’s Hwy, 2.4 mi. SE Governor’s Harbour, 25.174333, -76.2105, 26 vi 2014, J. Miller, M. Simon, D. Matthews, G. Goss, Bahamas Survey MGCL Accession No. 2014-15, D. Matthews Genitalia Prep. #1800, MGCL 239708 (MGCL). **Grand Bahama**: 1♂, vic. Owl’s Hole, 26.587496, -78.469854, 27 x 2014, J. Miller, M. Simon, R. Rozycki, D. Matthews, Bahamas Survey MGCL Accession No. 2014-31, MGCL 241372 (MGCL). **Great Exuma**: 1♂, SW of Hoopers Bay, 23.518167, -75.823667, 26 v 2014, J. Miller, M. Simon, D. Matthews, G. Goss, Bahamas Survey MGCL Accession No. 2014-14, MGCL 235147, KAA diss. #0002 (MGCL). 1♂, same as previous, but MGCL 234182 (MGCL). 1♀, same as previous, but MGCL 235148, KAA diss. #0003 (MGCL). 1♀, Simons Pt., 23.31.50, 75.47.30 [23.53238, -75.79478], 18 iv 1986, T. L. McCabe (MEM). 1♀, same as previous, but 17 i 1980 (MEM). **Great Inagua**: 1♀, 3 mi. SW of Morton dock, 21.022222, -73.685556, 27 vii 2014, M. J. Simon, G. Goss, Bahamas Survey MGCL Accession No. 2014-21, MGCL 237690 (MGCL). 1♀, 1.3 mi. NNE of Morton dock, 21.066111, -73.638056, 27 vii 2014, M. J. Simon, G. Goss, Bahamas Survey MGCL Accession No. 2014-21, MGCL 238059, KAA diss. #0004 (MGCL). **Long Island**: 1♂, NE of Whitehouse, 23.407167, -75.160500, 1 vi 2014, J. Miller, G. Goss, M. Simon, D. Matthews, Bahamas Survey MGCL Accession No. 2014-14, MGCL 235953 (MGCL). 1♀, Deadman’s Cay, vic. airport, 23.1755, -75.096333, 29 v 2014, J. Miller, G. Goss, M. Simon, D. Matthews, Bahamas Survey MGCL Accession No. 2014-14, MGCL 235817 (MGCL). **Mayaguana**: 1♂, Pirates Well, Baycaner Beach, 22.435833, -73.102222, 31 vii–1 viii 2014, M. J. Simon, G. Goss, Bahamas Survey MGCL Accession No. 2014-21, MGCL 237511, KAA diss. #0005 (MGCL). **New Providence**: 1♂, Adventure Learning Zoo off Marshall Rd., 25.004472, -77.353807, 10 iv 2014, J. Miller, M. Mundle, D. Matthews & Entomology Class, Bahamas Survey MGCL Accession No. 2014-10, MGCL 235078 (MGCL). **North Abaco**: 1♀, 1 mi. S of Blackwood Village, 26.785115, -77.431319, 6 vi 2016, J. Miller, M. Simon, G. Goss, D. Matthews, Bahamas Survey MGCL Accession No. 2016-09, MGCL 246725 (MGCL). **North Andros**: 1♂, Stanyard Creek Road, 24.730556, -77.886111, 6–7 vi 2013, J. Miller, M. Simon, G. Goss, A. Shahan, J. Y. Miller coll[ectio]n, MGCL Accession #2010-45, MGCL 233013 (MGCL). **San Salvador**: 1♂, beach NE of Gerace Research Centre, 24.120114, -74.461898, 24 vii 2015, D. Matthews, T. A. Lott, R. W. Portell, San Salvador Island Survey ID, D. Matthews et al., MGCL Acc. #2015-57, MGCL 243204, KAA diss. #0006 (MGCL). **South Andros**: 1♀, farm road N The Bluff, 24.130088, -77.59068, 30 iii 2014, J. Miller, M. Simon, R. Rozycki, D. Matthews, Bahamas Survey MGCL Accession No. 2014-9, MGCL 233852 (MGCL). **Cayman Islands**: **Grand Cayman**: 2♂♂, N coast of North Side, 9 vii 1938, C.B. Lewis, G.H. Thompson, Light Trap B, 17 iv–26 viii 1938, Oxf. Un. Cayman Is. Biol. Exped. (BMNH). 1♂, same as previous, but 5 vii 1938 (BMNH). 1♂, N coast of Rum Point, 4 v 1938, C.B. Lewis, G.H. Thompson, Light Trap, 17 iv–26 viii 1938, Oxf. Un. Cayman Is. Biol. Exped., KAA diss. #0038 (BMNH). 1♂, East end of East End, 16 v 1938, C.B. Lewis, G.H. Thompson, Light Trap B, 17 iv–26 viii 1938. Oxf. Un. Cayman Is. Biol. Exped., KAA diss. #0039 (BMNH). **Cayman Brac**: 2♀♀, West end of Cotton-tree Land, 22 v 1938, C.B. Lewis, G.H. Thompson, Light Trap B, 17 iv–26 viii 1938. Oxf. Un. Cayman Is. Biol. Exped, KAA diss. #0040 (BMNH). **Cuba**: **Havana**: 1♂, District of Habana [Havana], 1934, Father Roberto, Rothschild Bequest (BMNH). **Guantánamo**: 2♂♂, Baracoa, ix–[19]02, W. Schaus, 1905–244, one with KAA diss. #0037 (BMNH). 2♀♀, B[ara]coa, collection Wm. Schaus. KAA diss. #0197 (USNM). 1♂, Imías, La Farola, 700 m, vii 1990, V. O. Becker (VBC). **Holguín**: 3♂♂, Mayari, Loma de la Bandera, 12 vii 1990, V. O. Becker (VBC). 17♂♂, 8♀♀, Pinares de Mayari, 640 m, vii 1990, V. O. Becker (VBC). **Cienfuegos**: 1♂, nr. Pasa Caballos, 6 km S Cienfuegos, 13–14 ii 1981, 10 m, D.R. Davis (CUIC). **Pinar del Río**: 1♂, Mogote dos Hermanos, 3 km W Viñales, 7–8 ii 1981, ca. 150 m, D.R. Davis. USNM genitalia slide #142190 (USNM). 1♂, Las Animas, Sierra Rangel, 1500 ft., 28 iv [19]33, S.C. Bruner & A.R. Otero. E.E.A. Cuba, Ento. No. 10156. USNMdiss. #68330 [examined] (USNM). 10♂♂, 1♀, Sierra del Rosario, 400 m, 5–15 vi 1990, V. O. Becker (VBC). **Sancti Spíritus**: 2♂♂, Topes de Collantes, Canchánchara Repressa, 21°54.4'N, 80°1.4'W, 9 xii 1994, D.R. Davis; KAA diss. #0036; USNMENT01480181 (USNM). **Santiago de Cuba**: 1♂, Loma del Gato, Sierra del Cobre, Oriente, 24–30 ix 1935, 2600 ft., J. Acuña, S.C. Bruner, L.C. Scaramuzza. E.E.A. Cuba Ento. No. 10584. USNMdiss. #68331 [examined] (USNM). 1♂, La Gran Piedra, 1100 m, 18–21 vii 1990, O. Becker (VBC). 1♂, 1♀, Sierra Maestra, Pico Cuba, 1500 m, 31 vii 1990, V. O. Becker (VBC). 1♂, Turquino, 470 m, 27–29 vii 1990, V. O. Becker (VBC). **Dominican Republic**: **Azua**: 2♂♂, East side of crest, Sierra Martin Garcia, 7 km WNW Barrero, 18°21'N, 70°58'W, 860 m, 25–26 vii 1992, cloud forest adjacent to disturbed forest, C. Young, R. Davidson, S. Thompson, J. Rawlins, KAA diss. #0095, #0096 (CMNH). **Dajabon**: 1♀, 13 km S Loma de Cabrera, ca. 400 m, 20–22 v 1973, Don & Mignon Davis; KAA diss. #0094; USNMENT01480185 (USNM). **La Estrelleta**: 1♀, 4 km SE Rio Limpio, ca. 760 m, 24–25 v 1973, Don & Mignon Davis (USNM). **Pedernales**: 1♀, 26 km N Cabo Rojo, 18°06'N, 71°38'W, 730 m, 16 vii 1992, mesic deciduous forest with scattered pines, C. Young, R. Davidson, S. Thompson, J. Rawlins, KAA_DNA_0001, KAA diss. #0093 (CMNH). **Jamaica: Clarendon**: 2♀♀, nr. Jackson Bay Cave, 1.5 mi SE Jack. Beach, 50 ft., 4 v 1973, Don Davis, Mignon Davis; USNMENT01480187 (USNM).

##### Redescription.

A redescription was given in Austin et al. (2019). However, having now seen specimens from the Cayman Islands, Cuba, Hispaniola, Jamaica, and the Turks & Caicos, we can say a little more about the range of forewing pattern variation in *A. amatana*. Surprisingly, forewing pattern (Fig. 6) is most variable among populations on small islands such as the Florida Keys, The Bahamas, Turks & Caicos, and the Cayman Islands. Both the strongly contrasting (Fig. 6E, G) and weakly contrasting (Fig. 6F, H) forms of the males exist together on these islands. Peninsular Florida, Cuba, and Hispaniola possess predominantly the weakly contrasting form of *A. amatana* males. Twelve deciduous cornuti were present in one specimen examined.

##### Distribution.

*Argyrotaenia amatana* is one of only four Caribbean *Argyrotaenia* species known from lower elevations (*A. flavoreticulana*, The Bahamas; *A. kimballi*, The Bahamas; *A. vinalesiae*, Cuba) and it is by far the most common species in collections. It is widespread in the northern Caribbean, with records from Florida, The Bahamas, Turks & Caicos, Cuba, the Dominican Republic, Jamaica, and the Cayman Islands (Fig. 23). Most specimens were collected along the coast at elevations below 100 m. However, on both Cuba and Hispaniola there exist populations at much higher elevations (400–1500 m). On Jamaica, the species appears to be replaced by *A. jamaicana* at mid- to high elevations.

##### Ecology.

Polyphagous. The known hostplants for *A. amatana* are listed by Austin et al. (2019). This species can be found year-round at lower elevations, but may have a more restricted flight period at higher elevations on Cuba and Hispaniola.

##### Remarks.

We propose the synonymy of *A. ochrochroa* Razowski and *A. neibana* Razowski with *A. amatana* because the genitalia of the holotypes of these two species are indistinguishable from those of *A. amatana*. The forewings of the two species, though highly divergent, are within the range of variation of *A. amatana* (see Austin et al. 2019). Razowski (1999) did not refer to *A. amatana* in his diagnoses, and we suspect that this is because *A. amatana* had not been yet been recorded in the Caribbean when these two species were described.

*Argyrotaenia ochrochroa* Razowski, 1999 (Fig. 6B) is known from a single female collected in Providenciales, Turks & Caicos. Razowski mistakenly listed it as being from the Dominican Republic and it was not given a detailed diagnosis. This error was repeated in subsequent publications. Razowski & Becker, 2000b compared its genitalia to those of *Argyrotaenia nuezana*, from which it can be easily separated by forewing pattern alone. *Argyrotaenia ochrochroa* looks much like *A. amatana* but with unusually distinct gray suffusions on the wings. The genitalia are indistinguishable from those of *A. amatana* from neighboring islands in The Bahamas.

The case of *A. neibana* (Fig. 6A) is slightly more noteworthy because of the higher elevation from which the female holotype was collected (630 m). Similar specimens from Hispaniola and Cuba range in elevation from 400–1500 m. On Hispaniola, none are known from the coast, but this may be an artifact of sampling bias more than a distributional anomaly. We found no significant differences between the holotype genitalia and those of *A. amatana*, corroborating Austin et al. (2019). Mid-elevation males from Hispaniola have genitalia indistinguishable from those of *A. amatana*. Forewing patterns of the examined Hispaniolan and Cuban specimens match closely with the “typical” form of *A. amatana* from Florida. Unfortunately, we were only able to examine photographs of mid- and high-elevation Cuban specimens in ISEZ and VBC and unable to dissect males for comparison.

Among Caribbean taxa, COI sequence data strongly support a clade composed of *A. amatana*, *A. vinalesiae*, and *A. jamaicana* (Figs 3, 4). Maximum COI sequence divergence was 0.5% among barcoded specimens of *A. amatana* used in this study (n = 3). Minimum COI sequence divergence between *A. amatana* (n = 3) and *A. vinalesiae* (n = 1) was 1.6%; and for *A. amatana* (n = 3) and *A. jamaicana* 2.8% (n = 1).

#### Argyrotaenia flavoreticulana

Taxon classificationAnimaliaLepidopteraTortricidae

Austin & Dombroskie, 2019

B11FE21F-385B-52FE-AB56-BB2891D8351B

 Austin et al. 2019: figs 2a–c; 3c, d, h; 4b

Argyrotaenia flavoreticulana Austin & Dombroskie, 2019: 9

##### Diagnosis.

*Argyrotaenia flavoreticulana* is unlikely to be confused with any described Caribbean Archipini. Its straw yellow FW with obsolete banding (Austin et al. 2019: fig. 2a–c) separate it from all other Caribbean species in the tribe. See Austin et al. (2019) for a full diagnosis.

##### Type material.

***Holotype*** ♂: **The Bahamas: Great Exuma**: Simons Pt, 23.31.50, 75.47.30 [23.53238, -75.79478], 10 iv 1986, Tim L. McCabe, (CUIC) [examined]. ***Paratypes*** (2♂♂, 2♀♀): **The Bahamas: Long Island**: 1♂, blue hole E of Anderson, 23.533233, -75.237334, 31 v 2014, J. Miller, G. Goss, M. Simon, D. Matthews, Bahamas Survey MGCL Accession No. 2014-14, MGCL 236227, K.A. diss. #0008 (MGCL). 1♀, same as previous, but Bahamas Survey MGCL Accession No. 2014-14, D. Matthews Genitalia Prep. #1843 MGCL 236228 (MGCL). **South Andros**: 1♂, W of The Bluff Settlement, 24.106939, -77.557659, 29 iii 2014, J. Miller, M. Simon, R. Rozycki, D. Matthews, Bahamas Survey MGCL Accession No. 2014-9, D. Matthews Genitalia Prep. #1825, MGCL 233628 (MGCL). **Great Exuma**: 1♀, Simons Pt, 23.31.50, 75.47.30 [23.53238, -75.79478], 17 i 1980, Tim L. McCabe(TM) [all examined].

##### Description.

See Austin et al. 2019.

##### Distribution.

*Argyrotaenia flavoreticulana* is known from Great Exuma, Long Island, and South Andros Island in The Bahamas (Austin et al. 2019).

##### Ecology.

Nothing is known of the biology of *A. flavoreticulana*. Specimens have been collected from January to April.

#### Argyrotaenia kimballi

Taxon classificationAnimaliaLepidopteraTortricidae

Obraztsov, 1961

6DA265C1-35A9-5733-83DB-25C0F8DA47B4

 Austin et al. 2019: figs 2d; 3b, g; 4c

Argyrotaenia kimballi Obraztsov, 1961: 13

##### Diagnosis.

*Argyrotaenia kimballi* is most similar to *A. amatana*, which also occurs in The Bahamas. It can be separated from the latter by its conspicuously bicolored median fascia, which is uniformly colored in *A. amatana*.

##### Type material.

***Holotype*** ♂: **USA: Florida**: Highlands Co., Archbold Biological Station, 10 ii 1958, R. W. Pease, Jr., genitalia on slide, no. 509-Obr. (AMNH) [photo examined]. ***Paratypes*** (5♂♂, 1♀): **USA**: 2♂♂, same as holotype but 25 xii 1957 and 5 i 1958 [not examined]. 3♂♂, same as holotype but 31 xii 1959, 5 i 1960, and 14 i 1960, S.W. Frost [not examined] (Collection of C.P. Kimball, possibly donated to AMNH after Kimball’s death). 1♀, same as holotype but 22 ii 1958 (genitalia on slide, no. 510-Obr.) [not examined] (AMNH).

##### Additional material examined.

(5♂♂). **The Bahamas**: **North Andros**: 1♂, Captain Bill’s Blue Hole, 24.742046, -77.862031, 13 vi 2012, Mark Simon, Gary Goss, Rick Rozycki & Michael Simon, M. Simon MGCL Accession No. 2012-28, MGCL 233014 (MGCL). 1♂, 2.4 mi. S of Staniard Creek, dirt road W of Queen’s Hwy., 24.797594, -77.888264, 27 x 2011, J.Y. Miller, M. Simon, G. Goss, D. Matthews, MGCL Accession No. 2011-32, MGCL 233015 (MGCL). **South Abaco**: 1♂, Schooner Bay, coppice trail, 26.167000, -77.181167, 30 x 2014, J. Miller, M. Simon, R. Rozycki, D. Matthews, Bahamas Survey MGCL Accession No. 2014-31, D. Matthews Genitalia Prep. #1795, MGCL 238664 (MGCL); 1♂, Schooner Bay Institute, 26.161333, -77.187667, 31 x 2014, J. Miller, M. Simon, R. Rozycki, D. Matthews, Bahamas Survey MGCL Accession No. 2014-31, MGCL 241639 (MGCL); 1♂, vicinity of Sawmill Sink, 26.218346, -77.210170, 31 x 2014, J. Miller, M. Simon, R. Rozycki, D. Matthews, N. & M. Albury, Bahamas Survey MGCL Accession No. 2014-31, MGCL 241702 (MGCL).

##### Description.

See Austin et al. 2019.

##### Distribution.

*Argyrotaenia kimballi* is known in the USA from east Texas to Florida, north to Tennessee and Maryland. In the Caribbean, it has only been recorded from The Bahamas (Austin et al. 2019).

##### Ecology.

*Argyrotaenia kimballi* is reported to be a minor pest on *Citrus* in Florida (Bullock et al. 1997). Its food preference in the Caribbean is unknown, but it is likely a generalist.

#### Argyrotaenia bisignata

Taxon classificationAnimaliaLepidopteraTortricidae

Razowski, 1999

EDED22C1-37A1-5E06-AEA6-8A30C722F199

Figs 7A–D , 15A , 16F , 24B

Argyrotaenia bisignata Razowski, 1999: 310

##### Diagnosis.

*Argyrotaenia bisignata* (Fig. 7A–D) is most similar to *A. felisana* (Fig. 7E–H). It is most easily separated by distribution and male genitalia. *Argyrotaenia bisignata* is endemic to the Sierra de Bahoruco of the Dominican Republic and likely occurs in neighboring regions of Haiti (Fig. 24B). *Argyrotaenia felisana* occurs on every mountain range in the Dominican Republic except the western portion of Sierra de Bahoruco (Fig. 24B). Adults cannot be separated reliably by forewing pattern. Male genitalia of *A. bisignata* differ in possessing a significantly wider neck of the uncus (Fig. 15A) compared to *A. felisana* (Fig. 14E). In most specimens, dissection is not necessary; scales can be gently brushed from the tip of the abdomen to expose the critical structures. Female genitalia are typical of genus. Razowski (1999) mentioned the presence of a “minute basal sclerite at base of [corpus] bursae” and the absence of the basal sclerite of the ductus bursae. There is a sclerite near the base of the corpus bursae in the examined type material and one KAA dissection, but this character is present in other species of *Argyrotaenia* and may be variable. Females are best identified through association with males or by distribution.

##### Type material.

***Holotype*** ♂: **Dominican Republic: Pedernales**: 5 km NE Los Arroyos, 18°15'N, 71°45'W, 1680 m, 17–18 vii 1990, C. Young, J.E. Rawlins, S. Thompson [examined], Razowski genitalia slide #10711 [examined] (CMNH). ***Paratypes*** (16♂♂, 2♀♀): same as holotype or with dates 15–16 vii 1990 (1♂) or 28 vii 1990 (3♂♂, 1♀). Razowski genitalia slides #10712(♀) [slide examined]; #10713(♀), KAA_DNA_0004 [slide not examined]; 10714(♂) [slide not examined]; KAA diss. #0041 (♂, see remarks), KAA_DNA_0002; KAA diss. #0044(♂), KAA_DNA_0003 [9♂♂, 2♀♀ adults examined] (CMNH).

##### Additional material examined.

(5♂♂, 2♀♀). **Dominican Republic**: **Pedernales**: 5♂♂, 5 km NE Los Arroyos, 1680 m, 18°15'N, 71°45'W, 30 ix 1991, R. Davidson, C. Young, S. Thompson, J. Rawlins, cloud forest (4 CMNH, 1 CUIC). KAA diss. #0055 (CMNH). 1♀, La Abeja, 38 km NNW Cabo Rojo, 18°09'N, 71°38'W, 1250 m, 15 vii 1987, J.E. Rawlins, R.L. Davidson, green paratype label, not a paratype label (see remarks). 1♀, 5 km NE Los Arroyos, 18°15'N, 71°45'W, 1680 m, 17–18 vii 1990, C. Young, J.E. Rawlins, S. Thompson, green paratype label, not a paratype label (see remarks), Razowski genitalia slide #10715 [slide not examined].

##### Redescription.

**Male (n = 15). *Head*.** Typical of genus. Scales on vertex pale yellow. Frons predominantly dark brown, intermixed with pale yellow and mahogany red scales. Labial palpus with scales on lateral surface of all three segments dark brown, occasionally with mahogany red scales toward apex of second segment; second segment expanded apically. Medial surface of palpus pale yellow. Scape dark brown basally, pale yellow apically; sensillae approximately 0.75–1.00 × width of flagellomere, recurved; dorsal scales of flagellum alternating between a dark brown and pale yellow row. ***Thorax*.** Typical of genus. Dorsum of pro- and meso-thorax concolorous with vertex; tegulae similar, but intermixed with dark brown and mahogany red scales. Forelegs and midlegs with lateral surface dark brown. Hindlegs entirely pale yellow to white. Medial surface of legs pale yellow to white. FWL 7.0–8.5 mm (mean = 7.2 mm; n = 15); basal quarter of costa gently curved, straight beyond except for minute concavity along subapical blotch at apical third. Dorsal surface of forewing (Fig. 7A, B) distinctly bicolored, with antemedian and postmedian interfascia nearly white, with faint strigulae throughout, but most noticeably in the subterminal area. Basal fascia, median fascia, and subapical blotch brick red to brown. Tornal blotch faint. This combination gives most males of this species a very “clean” appearance. There is a small, but usually distinctive dark brown dot at the end of the discal cell. Fringe with apical half brick red, tornal half off-white. Dorsal surface of hindwing pale yellow to white, becoming pale brown towards apex, with faint strigulae throughout, most noticeably at apex. Fringe with short pale brown scales along entire outer margin and longer off-white scales also present along posterior half. Ventral surface of forewing dark brown, pale yellow along costa from 0.5 × length to apex with dark brown dots. Ventral surface of hindwing white to pale yellow with dark brown dots, larger than on dorsal surface, concentrated along costal edge. ***Abdomen*.** Vestiture with basal segments pale yellow, apical segments dark brown. Genitalia (Fig. 15A) with uncus widening gradually, bulb approximately 2 × width of neck, unmodified, rounded at apex; arms of gnathos broad, unmodified, abruptly deflexed near terminal plate, which is notched at base; tegumen unmodified; transtilla complete, unmodified; valvae rounded; sacculus to 0.33 ×; dense cluster of similar setae present at base of valvae; juxta diamond-shaped with shallow notch; phallus pistol-shaped, slightly bent at apex; caulis reduced; approximately 14–16 cornuti present in two specimens examined (including holotype), 0.33 × length of phallus, moderate in width, slightly undulate, deciduous.

**Female (n = 4). *Head*.** As in male except lateral surface of palpus sometimes with more prominent mahogany red scaling, antennae with sensillae minute, approximately 0.25 × width of flagellomere. ***Thorax*.** As in male in coloring on legs, thorax occasionally dark brown. Forewing (Fig. 7C, D) larger, with FWL 7.5–8.5 mm (mean = 8.1 mm; n = 4); concavity at distal third slightly more apparent in most specimens. Dorsal surface of forewing similar in pattern (Fig. 7C), but often less contrasting in color: some specimens with antemedian and postmedian interfasciae heavily suffused with red-orange (Fig. 7D). Fringe with short dark gray scales present basally on apical half; long pale red to off-white scales present on tornal half. Frenulum with three bristles. ***Abdomen*.** Vestiture as in male. Genitalia (Fig. 16F) with papillae anales triangular; apophyses posteriores approximately 0.5 × length of sternum VII; apophyses anteriores approximately 0.67 × length of sternum VII; sterigma lightly sclerotized, quadrate; ductus bursa narrow at base, gradually widening to corpus bursae; ductus seminalis arising at approximately 0.15 × length of ductus bursae; corpus bursa large, ovoid, with a small basal sclerite; signum long, thin, J-shaped; capitulum of signum globose, smooth.

##### Distribution.

*Argyrotaenia bisignata* is restricted to the Sierra de Bahoruco in the Dominican Republic (Fig. 24B). It is expected to occur in the Chaîne de la Selle of neighboring Haiti. Records are from 1250 to 2070 m elevation.

##### Ecology.

Nothing is known of the biology of *A. bisignata*. Specimens range in capture date from May to November, with most specimens examined taken in July.

##### Remarks.

Razowski included two female paratypes in his original description, but we have examined four females with paratype labels and have seen a fifth in ISEZ. According to ICZN Article 72.4.5, only the two listed in Razowski (1999) are to be considered paratypes (ICZN 1999). Because two female specimens with paratype labels share the same data labels, we have selected one as a paratype. We have affixed an additional label beneath the two specimens examined which were not included in Razowski (1999) explaining this. The same should be done with the specimen in ISEZ and any other such female specimens found.

One male paratype (KAA diss. #0041, KAA_DNA_0002) was found to differ from the rest of the type series in both forewing pattern and genitalia. COI barcoding suggests it is a close relative of *A. cryptica*, but differences in both forewing pattern and genitalia suggest that they may not be conspecific.

COI barcoding revealed 0% sequence divergence between the two specimens of *A. bisignata* sampled.

#### Argyrotaenia felisana

Taxon classificationAnimaliaLepidopteraTortricidae

Razowski, 1999

8E3B7934-9129-58BF-A870-8E7A69FF56C0

Figs 7E–H , 14E , 16E , 24B

Argyrotaenia felisana Razowski, 1999: 309

##### Diagnosis.

*Argyrotaenia felisana* (Figs 7E–H, 14E, 16E) most closely resembles *A. bisignata* (Figs 7A–D, 15A, 16F) in forewing appearance and genitalia. See the diagnosis under that species.

##### Type material.

***Holotype*** ♀: **Dominican Republic: Independencia**: Sierra de Neiba, just south of crest, 5 km WNW Angel Feliz, 1780 m, cloud forest, 18°41'N, 71°47'W, 13–15 x 1991, J. Rawlins, R. Davidson, C. Young, S. Thompson (CMNH) [examined], Razowski genitalia slide #10692 [examined] (CMNH).

##### Additional material examined.

(14♂♂, 35♀♀). **Dominican Republic: Azua**: 1♂, East side of crest, Sierra Martin Garcia, 7 km WNW Barrero, 18°21'N, 70°58'W, 860 m, 25–26 vii 1992, cloud forest adjacent to disturbed forest, C. Young, R. Davidson, S. Thompson, J. Rawlins, KAA diss. #0076 (CMNH). **Barahona**: 1♀ (abdomen missing), nr. Filipinas Larimar Mine, 6–11 vii 1993, R.E. Woodruff, KAA_DNA_0060 (FSCA). 1♀, Eastern Sierra Bahoruco, Reserva Cachote, 11.3 km NNW Paraiso, 18°05'54"N, 71°11'21"W, 1230 m, cloud forest with tree ferns, 3 v 2006, R. Davidson, C. Nunez, D. Koenig, J. Hyland, J. Fetzner, C. Young, J. Rawlins, KAA diss. #0056, KAA_DNA_0005 (CMNH). **Elías Piña**: 1♂, Sierra de Neiba, 9.0 km WSW Hondo Valle, 18°41'34"N, 71°46'52"W, 1843 m, 25 vi 2003, disturbed montane woodland with pine, J. Rawlins, C. Young, R. Davidson, C. Nunez, P. Acevedo, M. de la Cruz, KAA diss. #0046 (CMNH). **Independencia**: 1♂, 2♀♀, same data as holotype (1♂ CUIC, remainder CMNH). KAA diss. #0045(♂, CUIC); #0070(♀), KAA_DNA_0014 (CMNH). 1♂, 15♀♀, Sierra de Neiba near crest, 5.5 km NNW Angel Feliz, 18°41'N, 71°47'W, 1750 m, 21–22 vii 1992, dense cloud forest, J. Rawlins, S. Thompson, C. Young, R. Davidson (1♀ CUIC, remainder CMNH). KAA diss. #0042(♂), KAA_DNA_0017; #0050(♀); #0081(♀) (CMNH). 1♂, 6♀♀, Sierra de Neiba, south slope near summit, 4.0 km N Angel Feliz, 18°40'21"N, 71°46'05"W, 1825 m, 1–2 iv 2004, broadleaf cloud forest without pine, J. Rawlins, C. Young, R. Davidson, #0051(♀), #0074(♂) (CMNH). 1♂, same data as previous except 1 v 2006, J. Hyland, C. Young, R. Davidson, D. Koenig, J. Fetzner, J. Rawlins (CMNH). 1♀, Sierra de Bahoruco, north slope, 13.5 km SE Puerto Escondido, 1789 m, ecotonal *Pinus* grassland 18°12'18"N, 71°31'08"W, 24–25 xi 2004, J.E. Rawlins, C. Young, C. Nunez, V. Verdecia, W.A. Zanol, KAA diss. #0054, KAA_DNA_0006 (CMNH). **La Estrelleta [Independencia**]: 1♂, Sierra de Neiba at crest, 5.5 km WNW N Angel Feliz, 1800 m, 18°41'N, 71°47'W, 15 x 1991, cloud forest, R. Davidson, C. Young, S. Thompson, J. Rawlins, KAA diss. #0064 (CMNH). **La Vega**: 2♂♂, 1♀, La Palma, 12 km E of El Rio, 2–13 vi 1969, Flint & Gomez (1♂ CUIC, remainder USNM); KAA diss. #0077(♂), USNMENT01480223; USNMENT01480225 (♀) (USNM). 2♂♂, Convento, 12 km S of Constanza, 6–13 vi 1969, Flint & Gomez, KAA diss. #0079 (USNM). 1♂, Constanza, 2–6 vi 1969, Flint & Gomez (USNM). 1♀, Cordillera Central, 4.1 km SW El Convento, 18°50'37"N, 70°42'48"W, 1730 m, 31 v 2003, dense secondary evergreen forest with pine, J. Rawlins, R. Davidson, C. Nunez, C. Young, P. Acevedo, KAA diss. #0080, KAA_DNA_0018 (CMNH). 1♂, same data as previous except 1710 m, 14 xi 2002, secondary broadleaf forest, W.A. Zanol, C.W. Young, C. Staresinic, J. Rawlins, KAA diss. #0047 (CMNH). 1♀, 2.5 km SW Pinar Bonito, 18°51'N, 70°43'W, 1430 m, 26 ix 1992, riparian vegetation near stream in pine woodland, J. Rawlins, R. Davidson, M. Klingler, S. Thompson, KAA diss. #0071 (CMNH). **Monseñor Nouel**: 1♀, 1 km E Paso Alto de Casabito, 7 km NW La Ceiba, 1130 m, 19°02'N, 70°29'W, 28 vii 1992, cloud forest, R. Davidson, J. Rawlins, S. Thompson, C. Young (CUIC). **Peravia [San José de Ocoa**]: 1♂, 4♀♀, 3 km SW La Nuez, upper Rio Las Cuevas, 1880 m, 18°39'N, 70°36'W, 5–6 x 1991, cloud forest on river, J. Rawlins, R. Davidson, C. Young, S. Thompson (1♀ CUIC, remainder CMNH). KAA diss. #0043(♂), KAA_DNA_0016; #0082(♀), KAA_DNA_0015 (CMNH). **Puerto Plata**: 1♀, Pico El Murazo, north slope near summit, 19°41'N, 70°57'W, 910 m, 28 xi 1992, mesic deciduous forest, J. Rawlins, R. Davidson, M. Klingler, S. Thompson, KAA diss. #0053, KAA_DNA_0019 (CMNH).

##### Description.

**Male (n = 14). *Head*.** Typical of genus. Scales on vertex variable in color, usually with some combination of pale yellow, dark brown, or mahogany red. Frons dark brown, occasionally with mahogany red scales dorsally. Lateral surface of labial palpus with scales on first segment pale yellow, occasionally intermixed with dark brown scales; second and third segment dark brown and mahogany red. Medial surface of palpus intermixed with pale yellow and dark brown scales. Scape dark brown, nearly black, with a few mahogany red scales sometimes present apically. Sensillae approximately width of flagellomere, recurved; dorsal scales of flagellum alternating between a straw yellow and dark brown row. ***Thorax*.** Typical of genus. Dorsum of pro- and meso-thorax variable: either pale yellow, dark brown, or mahogany red or some combination thereof. Lateral surface of forelegs and midlegs dark brown; hindlegs pale yellow to white, tarsi and tarsal spurs warm brown. Medial surface of legs pale yellow to white. Forewing (Fig. 7G, H) with basal third of costa smoothly curved, straight beyond except for subtle concavity along subapical blotch at apical third; FWL 6.0–8.0 mm (mean = 6.8; n = 14). Dorsal surface of forewing with antemedian and postmedian interfasciae fascia light brown to white, with faint darker brown to black reticulations, which are most apparent near fringe. Basal fascia, median fascia, and subapical blotch dark brown or deep mahogany red; under magnification these areas tinted with gray or salmon pink scales, especially along inner margin. Tornal blotch faint to obsolete. Fringe with apical half salmon pink to mahogany red, occasionally with a few dark gray scales, tornal half concolorous with interfasciae. Dorsal surface of hindwing white to light brown, with faint dark brown strigulae, especially towards apex. Fringe with pale short brown scales present along entire margin, longer pale yellow scales present along entire margin, becoming darker at apex. Ventral surface of forewing warm brown, costa straw yellow with warm brown dots. Ventral surface of hindwing as on dorsal surface. ***Abdomen*.** Vestiture with first two segments pale yellow, remaining segments warm brown, becoming slightly darker terminally. Genitalia (Fig. 14E) with uncus extremely narrow at base, gradually widening to large bulb (acutely pointed in one population from near Constanza); apicoventral setae projecting laterally from bulb; arms of gnathos of unmodified, moderate, abruptly bent at terminal plate. Tegumen with small patch of sockets laterally; transtilla moderate, complete, unmodified; valvae ovoid; presaccular gap narrow, widening slightly at apex of valvae; sacculus to 0.33 × of valvae; juxta shallowly notched, with small patch of sockets laterally; phallus pistol-shaped, caulis reduced; approximately 5–18 cornuti observed: moderate, slightly undulate, approximately 0.25 × length of phallus, deciduous.

##### Redescription.

**Female (n = 36). *Head*.** As in male except scales on vertex predominantly pale yellow and antennal sensillae short, porrect, no more than 0.5 × width of flagellomere. ***Thorax*.** Dorsum of pro- and meso-thorax predominantly pale yellow, only rarely with dark brown of mahogany red scales, a few specimens with mahogany red posterior thoracic scale tuft. Tegulae concolorous with dorsum of pro- and meso-thorax. Legs as in male, but with hindlegs sometimes entirely warm brown. Dorsal surface of forewing (Fig. 7E, F) with slightly more pronounced concavity in some specimens (Fig. 7E) but nearly straight (Fig. 7F) in others; FWL 6.5–9.5 mm (mean = 8.0; n = 36); much wider dark brown or mahogany red median fascia as compared to male. Under magnification, the white antemedian and postmedian interfasciae almost completely overlaid by blue-gray and salmon pink scales, giving appearance of a much less contrasting overall forewing pattern and a slightly purple hue. Fringe with much more extensive dark gray scaling on apical half than in male, long brick red scales present from apex to near tornus. Frenulum with two or three bristles, asymmetrical in number in several specimens examined. ***Abdomen*.** Genitalia (Fig. 16E) with papillae anales triangular; apophyses posteriores approximately 0.5 × length of sternum VII; apophyses anteriores approximately 0.67 × length of sternum VII; sterigma broad, quadrate; ductus bursa approximately 1.5–2 × length of sternum VII, broadening anteriorly; ductus seminalis arising at approximately 0.25 × length of ductus bursae; corpus bursae ovoid, with or without a minute sclerite at base; signum long, J-shaped; capitulum of signum globose, smooth.

##### Distribution.

*Argyrotaenia felisana* appears to be the most widespread *Argyrotaenia* in the Dominican Republic, occurring on all major mountain ranges, but has not been recorded in the western Sierra de Bahoruco, where it is replaced by *A. bisignata* (Fig. 24B). Collection localities range from 860 to 1880 m elevation.

##### Ecology.

Nothing is known of the biology of *A. felisana*. Examined specimens were collected from April to November, suggesting multiple generations per year.

##### Remarks.

The above represents the first description of the male of *A. felisana*. Initial associations based on wing pattern and shared locality data were subsequently confirmed with COI barcodes.

The specific epithet of this species is based on an incorrect transcription by Razowski. The holotype label reads “Angel Feliz”, but Razowski erroneously transcribed this part of the label as “Angel Felis” in the original description. However, because there is no clear evidence of inadvertent error within the original publication, the incorrect spelling must be retained (ICZN Article 32.5.1).

A series of five males from the vicinity of Constanza deposited in USNM differ in having a slightly spade-shaped uncus but otherwise agree with other males in genitalia and wing pattern. COI sequence divergence among barcoded specimens of *A. felisana* ranged from 0% to 3.3% (n = 9), but in the absence of significant observed morphological differences between populations, we choose to treat *A. felisana* as a single broadly distributed species on Hispaniola.

#### Argyrotaenia nuezana

Taxon classificationAnimaliaLepidopteraTortricidae

Razowski, 1999

65FA6A8A-22FE-580F-BBAC-51683184A7C4

**Figure 8.**
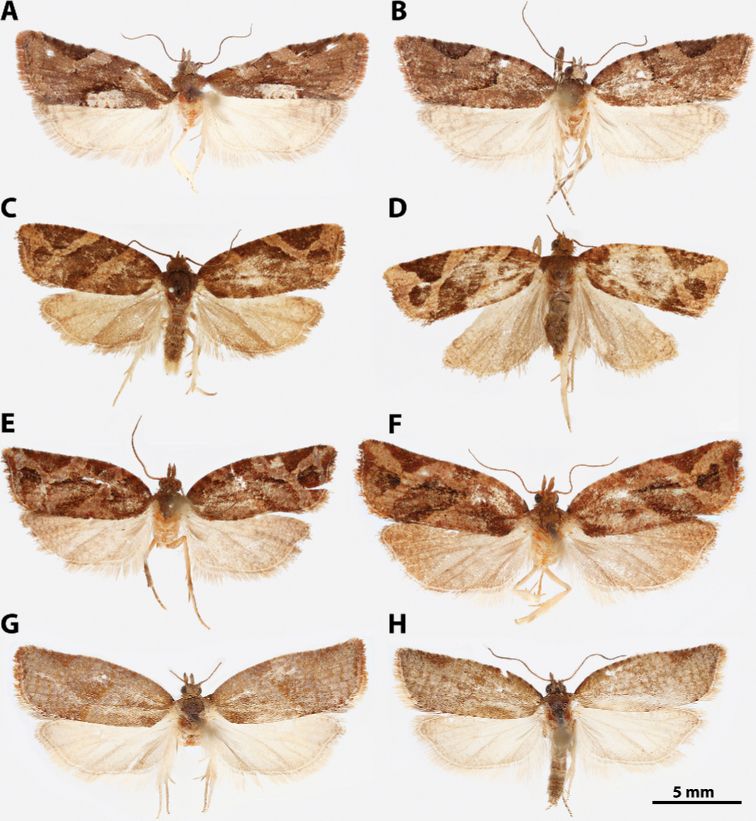
*Argyrotaenia* adults. **A***A. nuezana* holotype ♀, Dominican Republic (CMNH) **B***A. nuezana* ♂, Dominican Republic (CMNH) **C***A. cubae* ♂, Cuba (VBC) **D***A. cubae* ♀, Cuba (VBC) **E***A. browni* sp. nov. holotype ♂, Dominican Republic (CMNH) **F***A. browni* sp. nov. paratype ♀, Dominican Republic (CUIC) **G***A. razowskiana* sp. nov. paratype ♀, Dominican Republic (CMNH) **H***A. razowskiana* sp. nov. holotype ♂, Dominican Republic (CMNH).

Figs 8A, B , 14D , 16D , 25B

Argyrotaenia nuezana Razowski, 1999: 309
Argyrotaenia nuesana , misspelling in Razowski, 1999: 317

##### Diagnosis.

*Argyrotaenia nuezana* can be separated from all other Caribbean Archipini by its large size (FWL 8.5–10.5 mm), its dark chocolate brown color, and the presence of a dark L-shaped mark along the medial half of the inner margin of the median fascia in most specimens (Fig. 8A, B). In some females, this mark borders a distinctive rectangular patch of white scales (Fig. 8A). The male genitalia (Fig. 14D) are most similar to those of *A. cubae* (Fig. 14C) in that they both possess extremely wide folds of the valvae, but the uncus of *A. nuezana* expands apically. The female genitalia of *A. nuezana* (Fig. 16D) are typical of genus, but the signum is especially slender and strongly hooked.

##### Type material.

***Holotype*** ♀: **Dominican Republic: La Vega**: 24 km SE La Constanza, 18°44'N, 70°36'W, 2220 m, 16 viii 1990, grassland, J.E. Rawlins, S. Thompson [examined], Razowski genitalia slide #10694 [examined] (CMNH). ***Paratype*** (♀): **Dominican Republic**: **Peravia [San José de Ocoa**]: 3 km SW La Nuez, tributary to Rio Las Cuevas, 18°40'N, 70°36'W, 1870 m, 5–6 viii 1990. J Rawlins, S. Thompson [examined], Razowski genitalia slide #10693 [examined] (CMNH).

##### Additional material examined.

(12♂♂, 10♀♀). **Dominican Republic: La Vega**: 1♂, 6♀♀, 18 km SE Constanza, 18°46'N, 70°39'W, 2310 m, 25 xi 1992, M. Klingler, J. Rawlins, R. Davidson, S. Thompson, pine woodland near head of small canyon (1♀ CUIC; remainder CMNH, including 1♀ with KAA_DNA_0008). KAA diss. #0027 (♀) (CMNH). 4♂♂, 2♀♀, Reserva Cientifica Valle Nuevo, Sector La Nevera, 3 km WNW La Nuez, 2200 m, 18°42'N, 70°36'W, 7 x 1991, C. Young, S. Thompson, R. Davidson, J. Rawlins, mesic pine woodland (1♂, 1♀ CUIC; remainder CMNH). KAA diss. #0023 (♂), #0024 (♀), #0028 (♂) (CMNH). 2♂♂, 1♀, Cordillera Central, Reserva Valle Nuevo, La Nevera, 15.3 km SE Valle Nuevo, 18°41'39"N, 70°35'28"W, 2244 m, 25 v 2003, wet montane forest with pine, R. Davidson, C. Young, C. Nunez, J. Rawlins, P. Acevedo (1♂ CUIC, remainder CMNH). 1♀, Cordillera Central, Valle Nuevo Station, 5.2 km ESE Valle Nuevo, 18°46'42"N, 70°38'22"W, 2277 m, 23 v 2003, open pine-shrub woodland, C. Young, J. Rawlins, C. Nunez, R. Davidson, C. Acevedo, KAA diss. #0026, KAA_DNA_0007 (CMNH). 2♂♂, 5.2 km ESE Valle Nuevo, Valle Nuevo Field Station, 18°46'40"N, 70°38'22"W, 2120 m, 12–13 xi 2002, pine forest and grassland, W.A. Zanol, C.W. Young, C. Staresinic, J. Rawlins (CMNH). 3♂♂, Reserva Cientifica, Valle Nuevo, Sector La Nevera, 2150 m, 2 viii 1980, Allen Norrbom (1♂ CUIC, remainder CMNH).

##### Description.

**Male (n = 12). *Head*.** Typical of genus. Scales on vertex primarily pale yellow, brick red anteriorly. Scales on frons with dorsal dark gray, nearly black, occasionally with portions pale yellow. Lateral surface of labial palpus with first segment pale yellow, second segment dark brown to black, third segment primarily straw yellow, occasionally entirely light brown. Some specimens have brick red scales present on second and third segments. Medial surface of palpus pale yellow. Scape variable, with any combination of aforementioned colors. Sensillae variable in length and shape, short (0.5 × width of flagellomere) and relatively porrect in some individuals, as wide as flagellomere and curved in others; scales on dorsal surface of flagellomeres variable in color, usually dominated by pale yellow and brick red, dark brown or black scales sometimes present. ***Thorax*.** Typical of genus. Scales on dorsum of pro- and mesothorax chocolate brown, tegulae chocolate brown to light brown. Foreleg and midleg dark brown to black, pale yellow at apex of segments; medial surface pale yellow to white. Hindleg as in foreleg and midleg, but occasionally all pale yellow to white. Forewing (Fig. 8B) with costa gently curved along basal third, straight or nearly so beyond; FWL 8.5–9.5 mm (mean = 9.0; n = 12); apex distinctly acute, dorsal surface with warm brown base, overlaid with chocolate brown to black basal fascia, median fascia and subapical blotch. In fresher specimens, there are chalky blue-gray scales on inner margin of median fascia and subapical blotch. There is often a distinctive dark L-shaped mark extending parallel to inner margin and intersecting it at two-thirds wing length. Fringe salmon pink with short chalky blue-gray scales at vein terminals. Ventral surface light brown to white, inner margin and apical half of costa pale yellow. Dorsal surface of hindwing light brown to white, strigulae becoming apparent towards apex; fringe with short pale brown scales present along entire margin, longer pale brown scales also present, but becoming distinctly paler along posterior margin. Ventral surface as on dorsal surface. ***Abdomen*.** Vestiture with basal segments pale yellow to white, apical segments warm brown. Genitalia (Fig. 14D) with uncus broad, neck gradually widening apically, bulb quadrate, approximately 2.0 × wider than base of neck; socius obsolete; arms of gnathos broad and of uniform width; terminal plate robust, short, notched at base; tegumen with small patch of sockets present laterally; transtilla complete, uniform in width, unadorned; valva broad, semicircular, membranous; sacculus to 0.33 ×, presaccular gap extremely wide, occupying approximately half of surface of valva; juxta diamond-shaped with shallow notch, sockets for setae present laterally; phallus pistol-shaped, bent at nearly 90° angle; caulis small; cornuti not observed in dissected specimens, but sockets present, presumably deciduous.

##### Redescription.

**Female (n = 12). *Head*.** As in male except antenna with sensillae minute, no more than 0.25 × width of flagellomere. ***Thorax*.** Thorax, foreleg, and midleg as in male. Hindleg only rarely brown, usually pale yellow to white. Forewing (Fig. 8A) length 8.5–10.5 mm (mean = 9.5; n = 12). Dorsal surface of forewing similar to that of male, but some specimens have a rectangular patch of white scales at midpoint of inner margin bordering dark L-shaped mark on the median fascia; patch more developed in some specimens than in others. Frenulum with 2–4 bristles, occasionally asymmetrical in number. ***Abdomen*.** Vestiture as in male. Genitalia (Fig. 16D) with papillae anales broad, triangular, rounded laterally; apophyses posteriores approximately 0.67 × length of sternum VII, very thin; apophyses anteriores approximately 0.75 × length of sternum VII, very thin; sterigma broad, deep (difficult to see in slide-mounted specimens); ductus bursae narrow at base, widening gradually to corpus bursae; ductus seminalis arising at approximately 0.25 × length of ductus bursae; corpus bursae ovoid, with minute sclerite sometimes present at base of corpus bursae; signum long, thin, strongly hooked; capitulum with distinctly acute apex.

##### Distribution.

*Argyrotaenia nuezana* is restricted to the Cordillera Central of the Dominican Republic (Fig. 25B). All examined specimens are from La Vega and San José de Ocoa provinces, just south of Loma Alto de la Bandera at or above 1870 m elevation. Its range is likely restricted to this immediate area.

##### Ecology.

Capture dates range from March to November, suggesting multiple generations per year. Most include habitat labels mention the presence of pines, a putative host. The only native pine on Hispaniola is *Pinus occidentalis* Swartz (Pinaceae).

##### Remarks.

The above represents the first description of the male of *A. nuezana*. Association of the sexes was based on forewing pattern and shared localities and was subsequently confirmed with COI barcoding. COI sequence data of Caribbean species suggests that *A. nuezana* is sister to a Hispaniolan group of *Argyrotaenia* composed of *A. bisignata*, *A. cryptica*, *A. felisana*, *A. paradisei*, and *A. razowskiana* (Fig. 4). Whether or not this Hispaniolan group is monophyletic requires more extensive sampling of *Argyrotaenia*, especially in Central America.

#### Argyrotaenia cubae

Taxon classificationAnimaliaLepidopteraTortricidae

Razowski & Becker, 2010

9C0214AE-454D-5035-A1A9-682E146DEBBE

Figs 8C, D , 14C , 16C , 24C

Argyrotaenia cubae Razowski & Becker, 2010: 13

##### Diagnosis.

*Argyrotaenia cubae* most closely resembles *A. browni* in both forewing pattern and genitalia. Overall, *A. cubae* (Fig. 8C, D) has a more strongly contrasting appearance to the forewing without any hint of red scaling on the interfasciae compared to *A. browni* (Fig. 8E, F). Male genitalia of *A. cubae* (Fig. 14C) differ from *A. browni* (Fig. 14B) in possessing more pointed valvae with a significantly wider presaccular gap and a longer, thinner uncus. Female genitalia of *A. cubae* (Fig. 16C) differ from *A. browni* (Fig. 16B) in possessing a longer, thinner signum with an evenly rounded capitulum and broader papillae anales.

##### Type material.

***Holotype*** ♂: **Cuba: S[an]t[ia]go [de Cuba**]: Sier[ra] Maestra, P[ico] Cuba, 1500 m, 31 vii 1990, V.O. Becker Col. 73584 [photograph examined], genitalia slide #016 [figure examined] (VBC, see remarks below). ***Paratype*** (1♀): same data as holotype (VBC) [not examined], genitalia slide #017 [figure examined] (VBC, see remarks below).

##### Additional material examined.

(3♂♂, 4♀♀) **Cuba: S[an]t[ia]go [de Cuba**]: 3♂♂, 1♀, same data as type series. KAA diss. #0162 (♂), KAA_DNA_0022; #0163(♀), KAA_DNA_0023 (VBC). **Dominican Republic: Barahona**: 1♀, Eastern Sierra Bahoruco, Reserva Cachote, 12.8 km NE Paraiso, 18°05'54"N, 71°11'21"W, 1230 m, cloud forest with tree ferns, 19–21 v 2004, C. Young, C. Nunez, J. Rawlins, J. Fetzner; KAA diss. #0103 (CMNH). 1♀, same as previous except 21–23 iii 2004; KAA diss. #0100; KAA_DNA_0062 (CMNH). **La Vega**: 1♀, 4.1 km SW El Convento, 18°50'37"N, 70°42'48"W, 1710 m, secondary broadleaf forest, 14 xi 2002, W.A. Zanol, C.W. Young, C. Staresinic, J. Rawlins; KAA diss. #0102; KAA_DNA_0063 (CMNH).

##### Redescription.

**Male (n = 3). *Head*.** Typical of genus. Scales on vertex, frons, and lateral surface of labial palpus pale brown to dark chocolate brown. Scales on medial surface of labial palpus pale brown to straw yellow. Scape concolorous with vertex; sensillae approximately width of flagellomere, strongly curved; dorsal scales of flagellum alternating between a dark reddish-brown and golden yellow row. ***Thorax*.** Dorsum of pro- and meso-thorax warm chocolate brown, tegulae concolorous. Forelegs with scaling on lateral surface concolorous with thorax; midlegs with scaling on lateral surface pale brown; hindleg entirely pale to straw yellow; medial surface of all legs with scaling straw yellow. FWL 8.5–9.0 mm (mean = 8.7 mm; n = 3). Dorsal surface of forewing (Fig. 8C) with basal third gently curved, straight or nearly so beyond; basal fascia, median fascia, subapical blotch, and terminal blotch chocolate brown; antemedian and postmedian interfasciae pale brown, salmon pink and light red-orange scales present under magnification; fringe pale red-orange intermixed with a few chocolate brown scales, especially on apical half. Dorsal surface of hindwing light brown; strigulae faint, but more apparent towards apex; fringe light brown, slightly darker at apex. Ventral surface of forewing pale brown, dorsal pattern faintly visible. Ventral surface of hindwing pale brown, strigulae more apparent than on dorsal surface. ***Abdomen*.** Vestiture concolorous with dorsal surface of hindwing, straw yellow at apex. Genitalia (Fig. 14C) with uncus uniform in width, unmodified, rounded at apex, apicoventral setae long, projecting laterally on neck; arms of gnathos of unmodified, moderate, evenly curved; tegumen moderate; transtilla moderate, complete, unmodified; valvae semicircular, pointed at apex; presaccular gap wide, occupying approximately 0.5 × surface of valvae; sacculus apparent at base to 0.75 × of valvae, narrow beyond; juxta minutely notched; phallus pistol-shaped, caulis reduced; cornuti short, rounded at base, slightly curved at tip; four deciduous cornuti present in one specimen examined.

**Female (n = 4). *Head*.** As in males but vertex and frons intermixed with mahogany red scaling. Antennal sensillae short, porrect, no more than 0.5 × width of flagellomere. ***Thorax*.** As in male but with tegulae intermixed with mahogany red scales. Forewing (Fig. 8D) similar in pattern to male, but specimens from Hispaniola with fasciae darker brown. FWL 8.0–9.0 mm (mean = 8.5 mm; n = 4). ***Abdomen*.** Vestiture as in males. Genitalia (Fig. 16C) with papillae anales elongate, triangular, slightly swollen posteriorly; apophyses posteriores approximately 0.4 × length of sternum VII; apophyses anteriores approximately 0.8 × length of sternum VII; sterigma broad, quadrate; ductus bursa approximately 1 × length of sternum VII, broadening anteriorly; ductus seminalis arising at 0.25 × length of ductus bursae; corpus bursa ovoid; signum long, J-shaped; capitulum of signum evenly rounded, opposite-facing.

##### Distribution.

*Argyrotaenia cubae* is known from the Sierra Maestra range in southern Cuba, from the vicinity of Monumento Natural Miguel Domingo Fuerte on the eastern edge of the Sierra de Bahoruco in the Dominican Republic, and from the Cordillera Central in the Dominican Republic (Fig. 24C).

##### Ecology.

Nothing is known of the biology of *A. cubae*. Examined specimens were collected from March to November, suggesting multiple generations per year.

##### Remarks.

Both the holotype and paratype of *Argyrotaenia cubae* were found in ISEZ, not in VBC as listed in Razowski and Becker (2010). The females from the Dominican Republic agree well in forewing pattern, size, and genitalia to females from Cuba. Unfortunately, only one barcoded specimen yielded a COI sequence > 500 bp, so we are unable to discuss sequence divergence within this species with any level of significance.

#### Argyrotaenia browni sp. nov.

Taxon classificationAnimaliaLepidopteraTortricidae

9ED7C385-B6B4-59D4-8D83-F4AD7A789608

http://zoobank.org/2E320169-2469-492D-8D57-2595560A182E

Figs 8E, F , 14B , 16B , 24C

##### Diagnosis.

**Figure 9.**
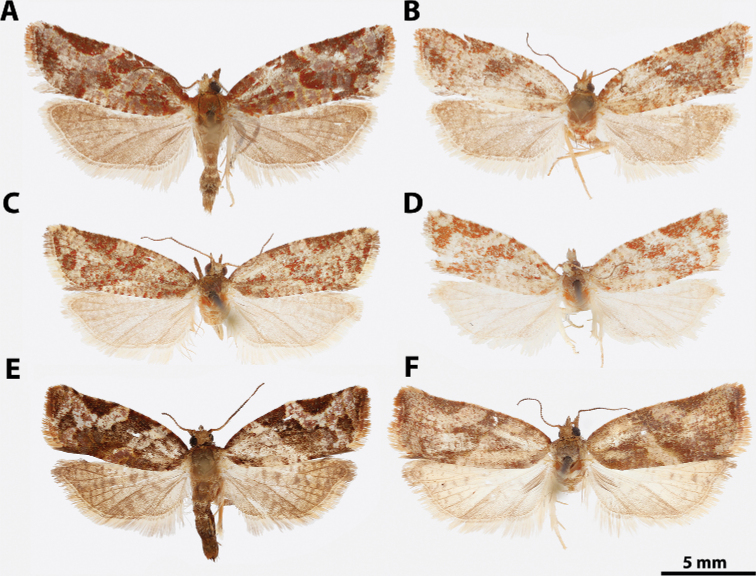
*Argyrotaenia* adults. **A***A. cryptica* sp. nov. holotype ♂, Dominican Republic (CMNH) **B***A. cryptica* sp. nov. paratype ♀, Dominican Republic (CMNH) **C***A. cryptica praeteritana* ssp. nov. holotype ♂, Dominican Republic (CMNH) **D***A. cryptica praeteritana* ssp. nov. paratype ♀, Dominican Republic (CMNH) **E***A. paradisei* sp. nov. holotype ♂, Dominican Republic (CMNH) **F***A. paradisei* sp. nov. paratype, ♀, Dominican Republic (CMNH).

*Argyrotaenia browni* most closely resembles *A. cubae* in both forewing pattern and genitalia. *Argyrotaenia browni* has a darker and redder overall hue to the forewing (Fig. 8E, F) compared to *A. cubae* (Fig. 8C, D). In addition, fresh specimens of *A. browni* are slightly more mottled and possess a distinct thin, black streak running parallel to the costa interrupting the median fascia. Male genitalia of *A. browni* (Fig. 14B) possess a broader uncus, more rounded valvae, and much narrower presaccular gap than *A. cubae* (Fig. 14C). Female genitalia of *A. browni* (Fig. 16B) possess a shorter, thicker signum, truncate capitulum, lateral edges of sterigma without significant sclerotization, and narrower papillae anales compared to *A. cubae* (Fig. 16C). Worn specimens could be confused with *A. paradisei* (Fig. 9E, F), with which it is sympatric on Sierra de Neiba. See the diagnosis of that species.

##### Type material.

***Holotype*** ♂: **Dominican Republic: Independencia**: Sierra de Neiba, south slope near summit, 4.0 km N Angel Feliz, 18°40'21"N, 71°46'05"W, 1825 m, 1–2 iv 2004, J. Rawlins, C. Young, R. Davidson, broadleaf cloud forest without pine (CMNH). HOLOTYPE *Argyrotaenia browni* Austin & Dombroskie [typed red label]. KAA diss. #0097 (CMNH). ***Paratypes*** (1♂, 2♀♀): **Dominican Republic**: **Elias Piña [Independencia**]: 1♀, Sierra de Neiba, 9.3 km SW Hondo Valle, 18°41'31"N, 71°47'03"W, 1901 m, 30 iv 2006, J. Rawlins, J. Hyland, R. Davidson, C. Young, D. Koenig, J. Fetzner, montane forest, *Podocarpus*, KAA diss. #0101 (CMNH). **Independencia**: 1♀, Sierra de Neiba near crest, 5.5 km NNW Angel Feliz, 18°41'N, 71°47'W, 1750 m, 21–22 vii 1992, J. Rawlins, S. Thompson, C. Young, R. Davidson, dense cloud forest. KAA diss. #0099, KAA_DNA_0021 (CUIC). **La Estrelleta [Independencia**]: 1♂, Sierra de Neiba at crest, 5.5 km WNW N Angel Feliz, 1800 m, 18°41'N, 71°47'W, 15 x 1991, R. Davidson, C. Young, S. Thompson, J. Rawlins, cloud forest, KAA diss. #0098, KAA_DNA_0020 (CMNH). All paratypes affixed with the following blue typed label: PARATYPE ♂/♀ *Argyrotaenia browni* Austin & Dombroskie, 2020.

##### Additional material examined.

(2♂♂, 2♀♀) **Dominican Republic: La Vega [Monseñor Nouel**]: 1♂, Loma del Casabito, 19°03'N, 70°31'W, 1390 m, wet cloud forest, 3 xi 2002, W.A. Zanol, C.W. Young, C. Staresinic, J. Rawlins, KAA diss. #0114, KAA_DNA_0032 (CMNH). 1♂ (abdomen missing), Cordillera Central, Loma Casabito, 15.8 km NW Bonao, 19°02'12"N, 70°31'08"W, 1455 m, evergreen cloud forest, east slope, 28 v 2003, J. Rawlins, C. Young, R. Davidson, C. Nunez, P. Acevedo, KAA_DNA_0033 (CMNH). **La Vega**: 1♀, Cordillera Central, Reserva Valle Nuevo, La Nevera, 15.6 km SE Valle Nuevo, 18°41'30"N, 70°35'24"W, dense cloud forest with pine, 2193 m, 25 iv 2006, J. Rawlins, C. Young, J. Fetzner, C. Nunez, KAA diss. #0125 (CMNH). **Peravia [San José de Ocoa**]: 1♀, 3 km SW La Nuez, upper Rio Las Cuevas, 18°39'N, 70°36'W, 1880 m, cloud forest on river, 5–6 x 1991, J. Rawlins, R. Davidson, C. Young, S. Thompson; KAA diss. #0134; KAA_DNA_0061 (CMNH).

##### Description.

**Male (n = 2). *Head*.** Typical of genus. Scales on vertex caramel brown. Scales on frons mahogany red intermixed with dark brown scales. Labial palpus with scales on lateral surface of all first and second segments predominantly dark brown, intermixed with pale yellow and mahogany red scales; third segment mostly pale yellow. Medial surface of palpus similar to lateral surface but with more pale yellow. Scape red-brown basally, pale yellow apically; sensillae approximately width of flagellomere, strongly curved; dorsal scales of flagellum alternating between scales of mahogany red and pale yellow. ***Thorax*.** Typical of genus. Dorsum of pro- and meso-thorax dark brown; tegulae variable, from mahogany red to dark brown to nearly white. Forelegs and midlegs dark brown with scattered pale yellow or mahogany red scales. Hindlegs variable, with some combination of dark brown, pale yellow, or mahogany scaling; tibial spurs of paratype bright orange. Medial surface of legs pale yellow to white. FWL 8.0–8.5 mm (mean = 8.3; n = 2); costa with basal third evenly curved, straight beyond. Ground color of forewing (Fig. 8E) chocolate brown, with antemedian and postmedian interfasciae warm brown with a slightly gray wash. More intricately colored under magnification, with submedian and postmedian fascia washed with salmon pink scales and bordered with light red-orange. Basal fascia, median fascia, subapical blotch, and tornal blotch intermixed with mahogany red scales. A diffuse black streak runs in the median area of the forewing to near the fringe. Fringe multicolored: short dark gray scales and longer salmon pink and brick red scales at apex, gradually replaced with short brick red scales and long salmon pink and light red-orange towards termen. Dorsal surface of hindwing pale brown, becoming darker towards apex, strigulae faint, becoming more apparent at apex. Fringe with pale brown scales present along entire margin, intermixed with a few brick red and gray scales at apex, longer off-white scales present along posterior half. Ventral surface of forewing warm brown, pale red-orange along costa. Ventral surface of hindwing white to pale brown with dark brown and salmon pink strigulae along costa to apex. ***Abdomen*.** Vestiture concolorous with dorsal surface of hindwing. Genitalia (Fig. 14B) with uncus moderate in width, uniform throughout, neck as wide as bulb, unmodified, rounded at apex, apicoventral setae long, projecting laterally; arms of gnathos moderate, unmodified, smoothly curved throughout; tegumen unmodified; transtilla complete, unmodified; valvae broadly rounded; sacculus to one-third; dense cluster similar setae present at costal half of base of valvae; juxta broadly rounded with shallow notch; phallus pistol-shaped, shallowly curved, caulis reduced; a single cornutus observed in one specimen, approximately 0.5 × length of phallus, moderate in width, straight, deciduous.

**Female (n = 2). *Head*.** As in male except lateral scales on palpus predominantly pale yellow. Sensillae minute, no more than 0.5 × width of flagellomere, porrect. ***Thorax*.** As in male but forewing (Fig. 8F) slightly larger: FWL 10.0–10.5 mm (mean = 10.3 mm; n = 2) and with black medial streak on dorsal surface of forewing more apparent. Fewer light red-orange scales on forewing, with markings more well-defined. Hindwing fringe with more extensive red and gray scaling at apex. Frenulum with three bristles. ***Abdomen*.** Vestiture as in male. Genitalia (Fig. 16B) with papillae anales narrow, slightly curved laterally; apophyses posteriores and anteriores both approximately 0.67 × length of sternum VII; sterigma lightly sclerotized, quadrate; ductus bursa 1.75 × length of corpus bursae, broad for almost entire length; ductus seminalis arising at approximately 0.2 × length of ductus bursae; corpus bursa small for genus, ovoid; signum moderate, curved; capitulum of signum truncate.

##### Etymology.

We take great pleasure in naming this species in honor of Dr. Richard L. Brown, W.L. Giles Distinguished Professor at Mississippi State University and Director of the Mississippi Entomological Museum, in honor of his unparalleled career in Lepidoptera morphology and systematics and for his role as a mentor to both authors.

##### Distribution.

*Argyrotaenia browni* is known from the Cordillera Central and the Sierra de Neiba in the Dominican Republic (Fig. 24C) and is expected in neighboring regions of Haiti as well. Collection localities range from 1390 to 2193 m elevation.

##### Ecology.

Nothing is known of the biology of *A. browni*. Specimens were collected from April to November.

##### Remarks.

We examined four specimens of *Argyrotaenia browni* from the Cordillera Central which resemble the type series but differ slightly in forewing pattern and uncus shape. Both males and females have a more subdued, less contrasting forewing pattern. The dissected male possesses a blunter uncus, but otherwise agree well.

A male and female paratype were barcoded but only a > 500 bp sequence was recovered for the female. Maximum sequence divergence between the Cordillera Central specimens barcoded was 0.3%. Sequence divergence for the paratype female and Cordillera Central specimens was 3.6–4.0%. For these morphological and molecular reasons, we exclude these specimens from the type series. We do not believe we have enough evidence to describe these Cordillera Central populations as a separate species, although future studies are warranted.

#### Argyrotaenia razowskiana sp. nov.

Taxon classificationAnimaliaLepidopteraTortricidae

8411647F-E760-5ED6-A736-2CCC4C02F605

http://zoobank.org/CD3FF846-9D47-44BF-9E09-0A0A572FC68E

Figs 8G, H , 15C , 17C , 23

##### Diagnosis.

*Argyrotaenia razowskiana* (Fig. 8G, H) is an externally unremarkable species. It more closely resembles males of *Claduncaria mesosignaria* (Fig. 11B) or *Clepsis deroni* (Fig. 12B) than any Caribbean *Argyrotaenia*. Male genitalia (Fig. 15C) are typical of genus and closely resemble several other Caribbean species but can be separated by the angled saccular margin at 0.33 × length (smoothly curved in all other Caribbean *Argyrotaenia*). Female genitalia (Fig. 17C) are typical of genus. The large size, plain brown forewing, hindwing without obvious strigulae, combined with typical *Argyrotaenia* genitalia should be sufficient to identify this species from any other archipine in the Caribbean.

##### Type material.

***Holotype*** ♂: **Dominican Republic: La Vega**: Cordillera Central, Valle Nuevo Station, 5.4 km ESE Valle Nuevo, 18°46'35"N, 70°38'20"W, 2260 m, 23 v 2003, C. Young, J. Rawlins, C. Nunez, R. Davidson, P. Acevedo, open, riparian grass-pine forest. HOLOTYPE *Argyrotaenia razowskiana* Austin & Dombroskie [typed red label] (CMNH). ***Paratypes*** (1♂, 2♀♀): **Dominican Republic**: **La Vega**: 1♂, 1♀, same data as holotype except 5.2 km ESE Valle Nuevo, 18°46'42"N, 70°38'22"W, 2277 m, open pine-shrub woodland. KAA diss. #0104 (♂, CUIC), KAA_DNA_0024; #0105 (♀, CMNH), KAA_DNA_0025. **Peravia [San José de Ocoa**]: 1♀, 3 km SW La Nuez, upper Rio Las Cuevas, 18°40'N, 70°36'W, 1850 m, 5–6 viii 1990, J. Rawlins, S. Thompson, KAA diss. #0106 (CMNH). All paratypes affixed with the following typed blue label: PARATYPE ♂/♀ *Argyrotaenia razowskiana* Austin & Dombroskie, 2020.

##### Description.

**Male (n = 2). *Head*.** Typical of genus. Scales on vertex with basal half white to pale yellow, apical half straw yellow. Frons straw yellow to light orange-red. Lateral surface of labial palpus with a mixture of dark brown and mahogany red scales; pale yellow on medial surface. Labial palpus missing in paratype. Scape with a mixture of straw yellow, dark brown, and mahogany red scales. Sensillae approximately width of flagellomere, recurved; scales on flagellomeres bicolored, with alternating rows of straw yellow and dark brown rows. ***Thorax*.** Typical of genus. Scales on dorsum of pro- and mesothorax almost completely missing in both males examined; the few remaining pale yellow. Tegulae predominantly warm brown, intermixed with straw yellow and mahogany red scales. Forelegs and midlegs dark brown on lateral surface. Hindlegs pale yellow to white, with tarsi and tibial spurs warm brown. Medial surface of legs pale yellow. Forewing (Fig. 8H) with basal third of costa gently curved, straight beyond (minutely concave along distal third in paratype); FWL 8.5–9.5 mm (mean = 9.0; n = 2). Dorsal surface of forewing uniformly warm brown, with faint dark brown reticulations throughout, except subapical blotch dark brown. Under magnification, mahogany red scales are also visible in this area and thinly scattered elsewhere. Fringe with short scales salmon pink basally, brick red apically except near tornus; longer scales red-orange, pale yellow at tornus. Dorsal surface of hindwing light grayish brown, strigulae absent, slightly darker along outer margin; fringe with short pale brown scales along entire margin, longer pale yellow to off-white scales also present along entire margin, becoming slightly darker at apex. Ventral surface of forewing warm brown with straw yellow costa with dark brown spots. Ventral surface of hindwing as on dorsal surface. ***Abdomen*.** Vestiture warm brown. Genitalia (Fig. 15C) with neck of uncus moderate, uniform in width, widening slightly to form rounded bulb; arms of gnathos moderate, unmodified, slightly bent; tegumen unmodified; transtilla complete, unmodified; valvae broad, nearly circular; sacculus to 0.25 ×; presaccular gap moderate; juxta diamond-shaped with shallow notch, sockets for setae present laterally; phallus elongate, pistol-shaped; caulis minute; approximately ten cornuti in one specimen examined: moderate, straight, approximately 0.25 × length of phallus, presumably deciduous.

**Female (n = 2). *Head*.** As in male except scales on vertex and frons with apical half warm brown or mahogany red, not straw yellow; sensillae short, porrect, 0.25–0.5 × width of flagellomere. ***Thorax*.** As in male except forewing (Fig. 8G) with darker, slightly more red overall hue, subapical blotch less distinct, FWL 9.5 mm (n = 2); under magnification salmon pink and mahogany red scales much more prevalent; forewing fringe with less extensive salmon pink and brick red scaling compared to male. Dorsal surface of hindwing with less extensive warm brown scaling near apex. ***Abdomen*.** Vestiture as in male. Genitalia (Fig. 17C) with papillae anales triangular; apophyses posteriores approximately 0.5 × length of sternum VII; apophyses anteriores approximately 0.67 × length of sternum VII; sterigma lightly sclerotized, thin, broadly bowl-shaped; ductus bursae widening gradually anteriorly; ductus seminalis arising at approximately 0.2 × length of ductus bursae; corpus bursae large, elongate ovoid; minute sclerite present near base; signum moderate in width, J-shaped; capitulum of signum prominent, evenly rounded.

##### Etymology.

We take great pleasure in naming this species after Dr. Józef Razowski in honor of his lifetime of immense contributions towards our current understanding of tortricid taxonomy.

##### Distribution.

*Argyrotaenia razowskiana* is known from La Vega and San José de Ocoa in the Dominican Republic, on the eastern edge of the Cordillera Central, south of Loma Alto de la Bandera (Fig. 23). Collection localities range from 1850 to 2277 m elevation.

##### Ecology.

Nothing is known of the biology of *A. razowskiana*. Capture dates are from May and August.

##### Remarks.

COI sequences for two specimens of *A. razowskiana* were identical.

#### Argyrotaenia cryptica

Taxon classificationAnimaliaLepidopteraTortricidae

2D583F7C-D7E2-5EB9-B7D6-65043287C0A3

http://zoobank.org/100DD712-C4C0-49B9-A6DD-413068223508

Figs 9A–D , 15D, E , 17E, F , 24D

##### Diagnosis.

*Argyrotaenia cryptica* (Fig. 9A–D) is unlikely to be confused with any other described Caribbean *Argyrotaenia*. Its narrow, elongate forewing with a distinctly acute apex, combined with its brick red fasciae serve to separate it from all other Caribbean Archipini. The male genitalia are most similar to those of *A. paradisei* (Fig. 15F), which possesses a shorter terminal plate of the gnathos and longer, more numerous cornuti compared to *A. cryptica* (Fig. 15D, E).

##### Remarks.

*A. cryptica* may represent a cryptic species complex and/or two or more lineages with high levels of incomplete lineage sorting. Several barcoded specimens with wildly different forewing patterns and genitalia clustered as *A. cryptica*. Unsurprisingly, barcoding may be of limited value in separating its two subspecies, which we describe based on subtle differences in wing pattern and genitalia, as well as distribution. We exclude the most extreme phenotypic examples from the type series of the two subspecies and restrict type series to a single locality or a set of closely situated localities. A Maximum Likelihood tree (Fig. 4) based on COI barcode sequence data strongly support that *A. cryptica* is sister to *A. paradisei*, a relationship supported by shared morphological traits. COI sequence divergence within barcoded specimens of *A. cryptica* was between 0.7% and 1.4% (n = 4), without respect to subspecies. Individual subspecies accounts follow.

#### Argyrotaenia cryptica cryptica ssp. nov.

Taxon classificationAnimaliaLepidopteraTortricidae

34F54575-60C7-58D5-86CB-8E10DE4C4D27

http://zoobank.org/6B24C28F-F2E4-46C6-A649-06AB82962F09

Figs 9A, B , 15E , 17E , 24D

##### Diagnosis.

*Argyrotaenia cryptica cryptica* can be separated most easily from *A. c. praeteritana* by range: *A. c. cryptica* is found in the Cordillera Central of the Dominican Republic, while *A. c. praeteritana* is found in the Sierra de Bahoruco of the Dominican Republic (and possibly neighboring regions of Haiti; Fig. 24D). The forewing pattern tends to be a little more washed out and the hindwing tends to be darker in *A. c. cryptica* (Fig. 9A, B) compared to *A. c. praeteritana* (Fig. 9C, D). In the male genitalia (Fig. 15E), the terminal plate of the gnathos is slightly shorter and less ventrally curved than in *A. c. praeteritana* (Fig. 15D). The bulb of the uncus is also slightly narrower. Female genitalia differ chiefly in the shape of the capitulum and size of basal sclerite in corpus bursae: capitulum acutely pointed and basal sclerite small in *A. c. cryptica* (Fig. 17E), while capitulum hooked and basal sclerite large in *A. c. praeteritana* (Fig. 17F), but with so few specimens examined it is unclear how variable these characters are.

##### Type material.

***Holotype*** ♂: **Dominican Republic: La Vega**: 23 km SE Costanza, 18°45'N, 70°37'W, 2225 m, 24–25 xi 1992, grassland with pines and scattered marshes, R. Davidson, M. Klinger, S. Thompson, J. Rawlins HOLOTYPE *Argyrotaenia cryptica cryptica* Austin & Dombroskie [typed red label] (CMNH). ***Paratypes*** (6♂♂, 2♀♀): **Dominican Republic**: **La Vega**: 2♂♂, 18 km SE Costanza, 18°46'N, 70°39'W, 2310 m, 25 xi 1992, pine woodland near head of small canyon, M. Klinger, J. Rawlins, R. Davidson, S. Thomas (1♂ CMNH, KAA_DNA_0030; 1♂ CUIC). 1♂, 5.2 km ESE Valle Nuevo, Valle Nuevo Field Station, 18°46'40"N, 70°38'22"W, 2120 m, 12–13 xi 2002, pine forest and grassland, W. A. Zanol, C. W. Young, C. Staresinic, J. Rawlins (CMNH). 1♂, 1♀, Cordillera Central Valle Nuevo Station, 5.2 km ESE Valle Nuevo, 18°46'42"N, 70°38'22"W, 2277 m, 23 v 2003, open pine-shrub woodland, C. Young, J. Rawlins, C. Nunez, R. Davidson, P. Acevedo (♂ CUIC, ♀ CMNH). KAA diss. #0171(♀) (CMNH). 1♂, Cordillera Central Valle Nuevo Station, 5.4 km ESE Valle Nuevo, 18°46'35"N, 70°38'20"W, 2260 m, 23 v 2003, open riparian grass-pine forest, C. Young, J. Rawlins, C. Nunez, R. Davidson, P. Acevedo. KAA diss. #0115 (CMNH). 1♂, Cordillera Central, 4.1 km SW El Convento, 18°50'38"N, 70°42'51"W, 1733 m, 31 v 2003, montane forest with pines near pasture, J. Rawlins, R. Davidson, C. Young, C. Nunez, P. Acevedo (CMNH). 1♀, Reserva Científica Valle Nuevo, Sector La Nevera, 3 km WNW La Nuez, 2200 m, 18°42'N, 70°36'W, 7 x 1991, mesic pine woodland, C. Young, S. Thompson, R. Davidson, J. Rawlins, KAA diss. #0118, KAA_DNA_0031 (CMNH). All paratypes affixed with the following typed blue label: PARATYPE ♂/♀ *Argyrotaenia cryptica cryptica* Austin & Dombroskie, 2020.

##### Description.

**Male (n = 7). *Head*.** Typical of genus. Scales on vertex white to maize yellow, a thin row of light orange scales sometimes present anteriorly. Scales on frons light red-orange. Labial palpus with scales of all three segments tricolored on lateral surface, intermixed with dark brown, mahogany red, and white scales; terminal segment occasionally entirely dark brown. Medial surface of palpus white with a few dark brown scales present anteriorly. Scape variable, with white, warm red-orange, or mahogany red scales, or some combination thereof. Sensillae approximately 1.5 × width of flagellomere, recurved; dorsal scales of flagellum alternating between a dark brown basal row and a pale buff apical row. ***Thorax*.** Typical of genus. Dorsum of pro- and meso-thorax red-orange intermixed with a few black scales; tegulae concolorous. Fore- and midlegs predominantly dark brown intermixed with pale yellow scales; hindlegs predominantly pale yellow to white intermixed with dark brown scales; tibiae and tibial spurs warm brown. Medial surface of legs pale yellow to white. FWL 7.5–9.5 mm (mean = 8.4; n = 7); costa with basal third very gently curved, straight beyond. Dorsal surface of forewing (Fig. 9A) with basal fascia, median fascia, and subapical blotch brick red; antemedian and postmedian interfasciae white (visible along costa), heavily suffused with pink-gray scales, obscuring much of the ground color; a few black scales scattered throughout, most conspicuous along costa. Fringe bicolored, apical half with long mahogany red scales and short dark gray scales, tornal half off-white with occasional small patches of dark gray scales; short portion of long scales along inner margin near tornus dark gray. Dorsal surface of hindwing gray, with faint strigulae towards apex. Fringe with short pale brown scales and longer off-white scales along entire margin. Ventral surface of hindwing warm brown, white and black spots present along costa. Ventral surface of hindwing as on dorsal surface but slightly paler and with strigulae more noticeable. ***Abdomen*.** Vestiture warm brown with terminal row of scales on each segment paler. Genitalia (Fig. 15E) with uncus moderate, unmodified, widening in apical half to bulb, apicoventral setae long; arms of gnathos unmodified, moderate, evenly curved, minutely hooked at apex, terminal plate robust, notched at base; tegumen widened slightly posteriorly, small patch of sockets for setae present laterally; transtilla broad, unmodified; valva circular; presaccular gap moderate, uniform in width; sacculus to 0.33 × of valva; juxta minutely notched. Phallus pistol-shaped, slightly downturned at apex, caulis minute, approximately eight cornuti observed: short, moderate in width, straight, approximately 0.25 × length of phallus, deciduous.

**Female (n = 2). *Head*.** As in male except with less extensive dark brown scaling on labial palpus, sensillae short, porrect, no more than 0.5 × width of flagellomere. ***Thorax*.** As in male but with less extensive pink-gray scaling on dorsal surface of forewing (Fig. 8B), which produces a more “washed-out” appearance. FWL 8.5–9.0 mm (mean = 8.7; n = 2). Fringe with less extensive gray scaling. Frenulum with two or three bristles, asymmetrical in number in one specimen examined. ***Abdomen*.** Vestiture similar to that of male. Genitalia (Fig. 16E) with papillae anales triangular, slightly rounded laterally; apophyses posteriores approximately 0.25 × length of sternum VII; apophyses anteriores approximately 0.75 × length of sternum VII; sterigma broad, well-sclerotized; ductus bursae broadening anteriorly; ductus seminalis arising at approximately 0.2 × length of ductus bursae; corpus bursa ovoid, minute sclerite at base of corpus bursa; signum thin to moderate, J-shaped; capitulum of signum acutely pointed.

##### Etymology.

The specific epithet *crypticus* Latin meaning hidden, refers to the possibility that *A. cryptica* may represent a cryptic species complex (see remarks under species account).

##### Distribution.

*Argyrotaenia cryptica cryptica* is restricted to the Cordillera Central of the Dominican Republic (Fig. 24D). Collection localities range from 1733 to 2310 m elevation.

##### Ecology.

Nothing is known of the biology of *A. c. cryptica*. Capture dates of examined specimens range from May to November, suggesting at least two generations per year.

##### Remarks.

See the remarks under the species account of *A. cryptica* for comments on this subspecies’ relationship to *A. c. praeteritana* and *A. paradisei*.

#### Argyrotaenia cryptica praeteritana ssp. nov.

Taxon classificationAnimaliaLepidopteraTortricidae

14538AE5-EAA0-5E03-A9FF-897F36C98979

http://zoobank.org/80C82507-D675-4ED7-8D1B-3287FFC787F6

Figs 9C, D , 15D , 17F , 24D

##### Diagnosis.

See diagnosis under *A. c. cryptica*.

##### Type material.

***Holotype*** ♂: **Dominican Republic: Pedernales**: 9.7 km NE Los Arroyos, 18°16'N, 71°44'W, 2070 m, 15–16 vii 1990, J. Rawlins, C.W. Young, S.A. Thompson, Razowski genitalia slide #10732 HOLOTYPE *Argyrotaenia cryptica praeteritana* Austin & Dombroskie [typed red label]. HOLOTYPE *Argyrotaenia cineriptera* Razowski [red label; see etymology below] (CMNH). ***Paratypes*** (7♂♂, 1♀): **Dominican Republic: Independencia**: 4♂♂, Sierra de Bahoruco, Loma del Toro, 18°17'16"N, 71°42'46"W, 2310 m, 7–8 xi 2002, meadow in pine woods, W. A. Zanol, C. W. Young, C. Staresinic, J. Rawlins (1♂ CUIC, remainder CMNH). KAA diss. #0113 (CMNH). 1♂, Sierra de Bahoruco, north slope, 13.5 km SE Puerto Escondido, 18°12'24"N, 71°30'54"W, 24–26 iii 2004, broadleaf *Pinus* dense woodland, R. Davidson, J. Rawlins, C. Young, D. Nunez, M. Rial (CUIC). 1♂, Sierra de Bahoruco, north slope, 18°41'31"N, 71°35'35"W [18–17–30N, 71–43–08W], 2116 m, broadleaf forest with pine, 8 xi 2002 W.A. Zanol, C.W. Young, C. Staresinic, J. Rawlins, KAA_DNA_0028 (CMNH). **Pedernales**: 1♂, 1♀, same data as holotype, Razowski genitalia slide #10731(♂); #10733(♀), KAA_DNA_0029 (CMNH). PARATYPE *Argyrotaenia cineriptera* Razowski [green label; see etymology below]. All paratypes affixed with the following typed blue label: PARATYPE *Argyrotaenia cryptica praeteritana* ♂/♀ Austin & Dombroskie, 2020.

##### Description.

**Male (n = 8). *Head*.** Typical of genus. Scape on vertex white to pale yellow, dark gray with white apices in one specimen examined. Scales on frons straw yellow to red-orange. Lateral surface of labial palpus variable, sometimes entirely warm brown intermixed with pale yellow scales, other times red-orange with terminal segment dark brown. Medial surface of labial palpus pale yellow. Scape equally variable, ranging from pale yellow to red-orange. Sensillae approximately 1.5 × as long as width of flagellomere; recurved in some individuals but not so in others; dorsal scales of flagellum alternating between a warm brown basal row and a nearly white apical row; apical row expanded slightly. ***Thorax*.** Typical of genus. Scales on dorsum of pro- and mesothorax variable, pale yellow or warm brown, but most commonly mahogany red. Tegulae concolorous with pro- and mesothorax except with a few pale yellow scales present apically. Forelegs entirely dark brown on lateral surface in specimens from Pedernales, suffused with mahogany red in specimens from Independencia. Midlegs and hindlegs warm brown intermixed with pale yellow scales, especially so on hindlegs. Forewing (Fig. 9C) with basal third very gently curved, straight beyond; FWL 7.5–9.0 mm (mean = 8.7; n = 8). Dorsal surface of forewing similar to *A. c. cryptica* (see description for that subspecies), but less washed out, giving a “grainier” appearance to it. Fringe without the red scaling present in *A. c. cryptica.* Dorsal surface of hindwing white in specimens from Pedernales, darker in specimens from Independencia. Ventral surfaces of both wings identical to *A. c. cryptica.****Abdomen*.** Vestiture warm brown with terminal row of scales on each segment white. Genitalia (Fig. 15D) with uncus moderate at neck, broadening to a rounded bulb with long apicoventral setae; socius obsolete; arms of gnathos moderate, unmodified, evenly curved; terminal plate moderate, distinctly curved, notched at base; tegumen with small patch of sockets present laterally; transtilla complete, uniform in width, unmodified; valvae nearly circular; sacculus to 0.33 × presaccular gap moderate, uniform in width throughout; juxta diamond-shaped with shallow notch, sockets present laterally. Phallus pistol-shaped, elongate, slightly downturned at apex; caulis small; approximately ten cornuti observed in one specimen: short, moderate in width, slightly undulate, approximately 0.2 × length of phallus, deciduous.

**Female (n = 1). *Head*.** As in male except with vertex and frons pale yellow to white, lateral surface of labial palpus pale yellow intermixed with a few warm brown scales; sensillae short, porrect, no more than 0.5 × as long as width of flagellomere**. *Thorax*.** As in male; forewing (Fig. 9D) as in male, FWL 7.5 mm. ***Abdomen*.** As in male. Genitalia (Fig. 17F) with papillae anales broad, triangular, rounded slightly laterally; apophyses anteriores and posteriores similar in length to those of *A. c. cryptica* (sternum VII removed prior to examination); sterigma moderate, bowl-shaped, thin laterally; ductus bursae narrow at base, widening gradually to corpus bursae; ductus seminalis arising at approximately 0.2 × length of ductus bursae; corpus bursae ovoid, with distinct, tooth-like sclerite at base; signum long, slightly hooked; capitulum irregularly rounded with conspicuous hook at apex.

##### Etymology.

The specific epithet is from *praeteritus*, Latin meaning “passed over,” referring to the fact that this taxon was known to Razowski, but unpublished. His manuscript name for it was ‘*cineriptera*’.

##### Distribution.

*Argyrotaenia cryptica praeteritana* is restricted to the Sierra de Bahoruco in the Dominican Republic (Fig. 24D). It likely occurs in neighboring regions of Haiti. Collection localities range from 1807 to 2310 m elevation.

##### Ecology.

Nothing is known of the biology of *A. c. praeteritana*. Examined specimens were collected in March, July, or November, suggesting multiple generations per year.

##### Remarks.

Razowski was the first to identify this species but did not publish on it. He had identified and labeled three specimens to serve as the type series. We did not remove these labels but added additional holotype/paratype labels beneath them. There is a discrepancy in the label data of one male specimen from Independencia. The label reads “Sierra de Bahoruco” but the coordinates are for the Sierra de Neiba. After comparing coordinates from specimens collected the previous night and discussing the situation with John Rawlins (CMNH), we interpret the coordinates to be incorrect. Dr. Rawlins kindly supplied us with the correct coordinates. See the remarks under the species account of *A. cryptica* for comments on this subspecies’ relationship to *A. c. cryptica* and *A. paradisei*.

#### Argyrotaenia paradisei sp. nov.

Taxon classificationAnimaliaLepidopteraTortricidae

625FFE3A-A9DD-556A-B50A-2ED1B775E1CE

http://zoobank.org/BBF54288-7210-4E2E-91EF-966666EEE3EA

Figs 9E, F , 15F , 17D , 24D

##### Diagnosis.

Undamaged males of *Argyrotaenia paradisei* (Fig. 9E) are unlikely to be confused with any other Caribbean *Argyrotaenia*. Worn specimens, however, could be confused with males of *A. browni* (Fig. 8E), with which it is sympatric, but lack the strongly contrasting off-white interfasciae present in *A. paradisei*. The genitalia (Fig. 15F) are distinct, however. The uncus of *A. paradisei* possesses a distinct bulb with setae only in the apicoventral area. In *A. browni* (Fig. 14B), the neck of the uncus is of uniform width throughout and possesses ventral setae across its entire length. Females of *A. paradisei* (Fig. 9F) are strikingly different from males and look like paler versions of *A. felisana* (Fig. 7E, F). Female genitalia (Fig. 17D) are typical of genus but have an unusually large basal plate the of signum; females are best identified through association with males.

##### Type material.

***Holotype*** ♂: **Dominican Republic: Independencia**: Sierra de Neiba near crest, 5.5 km NNW Angel Feliz, 18°41'N, 71°47'W, 1750 m, 21–22 vii 1992, dense cloud forest, J. Rawlins, S. Thompson, C. Young, R. Davidson HOLOTYPE *Argyrotaenia paradisei* Austin & Dombroskie [typed red label] (CMNH). ***Paratypes*** (5♂♂, 2♀♀): **Dominican Republic: Independencia**: 4♂♂, 1♀, same data as holotype (1♂ CUIC, remainder CMNH). KAA diss. #0049(♀), KAA_DNA_0064; #0116(♂), KAA_DNA_0026 (CMNH). **San Juan**: 1♂, Sierra de Neiba, Sabana del Silencio, 10.1 km SSW El Cercado, 18°39'07"N, 71°33'26"W, 2017 m, 16–17 xi 2004, cloud forest with juniper, *Danthonia*, J. Rawlins, C. Young, C. Nunez, V. Verdecia, W. Zanol, KAA diss. #0117, KAA_DNA_0027 (CMNH). 1♀, Sierra de Neiba, 9.3 km SSW El Cercado, 18°39'19"N, 71°32'49"W, 1968 m, 18–19 xi 2004, J. Rawlins, C. Young, C. Nunez, V. Verdecia, W. Zanol, KAA diss. #0073, KAA_DNA_0057 (CUIC). All paratypes affixed with the following typed blue label: PARATYPE ♂/♀ *Argyrotaenia paradisei* Austin & Dombroskie, 2020.

##### Description.

**Male (n = 6). *Head*.** Typical of genus. Scales on vertex pale yellow to straw yellow, a few dark brown or mahogany red scales sometimes present anteriorly. Frons with scaling mahogany red or dark brown. Labial palpus with lateral surface predominantly dark brown to black, a few mahogany red scales sometimes present on second segment; medial surface pale yellow. Scape dark brown to straw yellow. Sensillae approximately 1.5 × width of flagellomere, recurved; dorsal scales of flagellum alternating between a dark brown basal row and a straw yellow apical row. ***Thorax*.** Typical of genus. Dorsum of pro- and meso-thorax dark brown; tegulae concolorous with a few white scales posteriorly. Lateral surface of legs dark brown, hindlegs sometimes intermixed with pale yellow scales; medial surface of legs pale yellow to white. Forewing (Fig. 9E) with costa gently curved along basal third, straight or nearly so beyond, minutely concave along subapical blotch in some specimens; FWL 7.5–8.0 mm (mean = 7.8; n = 6). Dorsal surface of forewing with basal fascia, median fascia, subapical blotch, and tornal blotch dark red-brown with faint black reticulations throughout; antemedial and postmedial interfasciae off-white, but also with dark reticulations, contrasting strongly with ground color in most specimens. Overall forewing appearance for most specimens has a very crisp, yet strongly mottled appearance to it. Under magnification, blue-gray scales are sometimes present in median fascia. Fringe dark brick red with longer salmon pink scales, pale yellow at tornus. Dorsal surface of hindwing warm brown with distinct strigulae. Fringe with short pale brown scales present along entire margin, longer pale yellow scales also present, but becoming pale brown at apex and along posterior margin. Ventral surface of forewing dark brown, white spots present along costa. Ventral surface of hindwing as on dorsal surface, but slightly paler and more contrasting strigulae. ***Abdomen*.** Vestiture with segments dark brown ventrally, terminal row of scales on each segment white. Genitalia (Fig. 15F) with uncus moderate in width, widening apically to form rounded bulb, long apicoventral setae projecting laterally from bulb; arms of gnathos unmodified, moderate in width, terminal plate robust, minutely hooked apically, notched at base; tegumen unmodified, with small patch of sockets present laterally; transtilla moderate, even in width throughout, complete, unmodified; valvae nearly circular; presaccular gap moderate in width, even to apex; sacculus to 0.33 × of valvae; juxta shallowly notched, rounded laterally, sockets present laterally of notch; phallus pistol-shaped, slightly down-curved apically, caulis reduced, approximately 15–20 cornuti observed in two specimens examined, moderate in width and length, slightly undulate, approximately 0.25 × length of phallus.

**Female (n = 2). *Head*.** As in male except lateral surface of labial palpus with black scaling restricted to ventral and apical portions of second segment, predominantly mahogany red on lateral surface of other segments, scattered straw yellow scales present. Antenna with sensillae only observable ventrally, no more than 0.5 × width of flagellomere. ***Thorax*.** Thorax, foreleg, and midleg as in male. Forewing (Fig. 9F) length 8.0–8.5 mm (mean = 8.3; n = 2). Dorsal surface of forewing with basal fascia, median fascia, and subapical blotch dark brown, but heavily suffused with mahogany red and purple-gray scales under magnification; antemedian and postmedian interfasciae pale brown; fringe as in male but paler. In one paratype postmedian interfascia suffused with dark brown scales as to obscure it entirely. Dorsal surface of hindwing paler than male, but with strongly contrasting dark cubital pecten; strigulae less contrasting compared to male; fringe with less extensive long pale brown scales at apex and along posterior margin. Ventral surface of forewing pale brown, white along portions of costa, dark brown at apex. Ventral surface of hindwing white with strigulae strongly contrasting. Frenulum with two or three bristles. ***Abdomen*.** Vestiture as in male. Genitalia (Fig. 17D) with papillae anales broad, triangular, rounded laterally; apophyses posteriores approximately 0.5 × length of sternum VII, widened anteriorly; apophyses anteriores approximately 0.75 × length of sternum VII, widened anteriorly; sterigma deep, lightly sclerotized ventrally; ductus bursae narrow at base, widening gradually to corpus bursae; ductus seminalis arising at approximately 0.2 × length of ductus bursae; corpus bursae ovoid, with basal sclerite not observed; signum long, moderate, slightly curved (broken in Fig. 17D); capitulum globose, evenly-rounded, opposite-facing.

##### Etymology.

We take great pleasure in naming this species after Dr. Chris Paradise, professor and chair of biology at Davidson College, who was the undergraduate advisor and a mentor of KAA.

##### Distribution.

*Argyrotaenia paradisei* is known from two localities in the Sierra de Neiba of the Dominican Republic (Fig. 24D). It likely occurs in neighboring regions of Haiti. Collection localities range from 1750 to 2017 m elevation.

##### Ecology.

Nothing is known of the biology of *A. paradisei*. Capture dates of examined specimens are June, July, and November, suggesting at least two generations per year.

##### Remarks.

This is among the most strongly sexually dimorphic Caribbean *Argyrotaenia*. DNA barcoding was required to associate sexes. See remarks under *A. cryptica* regarding this species’ relationship to that species. Maximum COI sequence divergence within sampled *A. paradisei* was 0.1% (n = 4). One sequence (KAA_DNA_0059) clusters with *A. paradisei* based on COI data, but significant differences in both forewing pattern and genitalia make us question if it is conspecific.

#### Claduncaria

Taxon classificationAnimaliaLepidopteraTortricidae

Razowski, 2000, in Razowski & Becker, 2000a

0DC5E9FC-8B70-5028-A16E-E8146EB37B45

##### Type species.

*Cladotaenia ochrochlaena* Razowski, 1999

*Claduncaria* Razowski, 2000, in Razowski & Becker, 2000a: 208

*Cladotaenia* Razowski, 1999 (homonym of *Cladotaenia* Cohn, 1901): 312

##### Remarks.

Because we expand the concept of *Claduncaria*, which is endemic to the Greater Antilles, a new generic diagnosis and description is presented here.

##### Diagnosis.

Male genitalia (Fig. 18) either with uncus either apically broadened or divergently bifid; terminal plate of gnathos either vertically bifid or simple; transtilla with lateral processes. Female genitalia (Fig. 19) with ductus bursae not coiled; capitulum absent; signum absent or reduced.

##### Description.

Labial palpus 1.5–2 × width of compound eye; second segment expanded apically; ocellus small, separated from compound eye by approximately 1–1.5 × width of ocellus; chaetosemata 0.25–0.75 × length of scales on vertex; metathorax without dorsal scaling, with a small patch of pale yellow setae present instead. Costal fold absent; costa with basal third gently curved, straight beyond or nearly so. Male genitalia with a vertically bifid terminal plate of gnathos and broad, apically rounded valvae (*ochrochlaena* group) or simple terminal plate of gnathos and elongate, apically acute valvae (*mesosignaria* group); uncus either divergently bifid or apically broadened; socii present as small setose raised nubs (absent in *Cla. rufochlaena*); transtilla with lateral processes; phallus pistol- or dagger-shaped, sharp at apex, caulis variable. Female genitalia with papillae anales laterally notched and with distinct ventroposterior grooves (*ochrochlaena* group) or large and posteriorly swollen (*mesosignaria* group); sterigma well-sclerotized; colliculum present; signum reduced or absent entirely; capitulum absent. Some species sexually dimorphic in forewing coloration.

### Key to species of *Claduncaria*^2^

**Table:** 

1	Male with terminal plate of gnathos vertically bifid; valvae broad, apically rounded (Fig. 18A–C); female with papillae anales laterally notched and with distinct ventroposterior groove (Fig. 19A–D)	***ochrochlaena* group, 2**
–	Male with terminal plate of gnathos not vertically bifid; valvae elongate, apically acute (Fig. 18D–G); female with papillae anales conspicuously swollen apically, never with distinct ventroposterior groove (Fig. 19E–G)	***mesosignaria* group, 5**
2	Male with apically-quadrate arms of uncus (Fig. 18A, C); female with signum present (Fig. 19A, C, D)	**3**
–	Male with apically-rounded arms of uncus (Fig. 18B); female with signum absent (Fig. 19B); Hispaniola	***Cla. ochrochlaena***
3	Male with terminal plate of gnathos with vertically-paired processes acute (Fig. 18C); females with colliculum ring-like (Fig. 19C); Hispaniola	***Cla. rawlinsana* sp. nov.**
–	Male with terminal plate of gnathos with vertically-paired processes rounded (Fig. 18A); female with colliculum tube-like (Fig. 19A, D)	**4**
4	Female with groove in ventroposterior portion of papillae anales large, occupying at least 0.75 × length of posterior edge (Fig. 19D); male unknown; Hispaniola	***Cla. praedictana* sp. nov.**
–	Female with groove in ventroposterior portion of papillae anales moderate, occupying approximately 0.5 × length of posterior edge (Fig. 19A); male with vertically-paired processes terminally rounded, symmetrical (Fig. 18A); Cuba	***Cla. maestrana***
5	Jamaica	**6**
–	Cuba or Hispaniola	**7**
6	Male uncus divergently bifid (Fig. 18D); female unknown; Jamaica	***Cla. rufochlaena***
–	Female with signum absent (Fig. 19E); male unknown; Jamaica	***Cla. chalarostium***
7	FWL short (6.0–7.0 mm), uncus distinctly Y-shaped, notched mesally, not widening until 0.5 × length (Fig. 18G); female unknown; Hispaniola	***Cla. taino* sp. nov.**
–	FWL long (8.0–9.0 mm), male with uncus only with shallow indentation mesally, widening from base (Fig. 18E, F)	**8**
8	Male with uncus at apex 3 × width of neck (Fig. 18E); female with signum well-developed, at least 3 × as long as width at base (Fig. 19G); Hispaniola	***Cla. mesosignaria***
–	Male with uncus at apex no more than 2 × width of neck (Fig. 18F); female with signum reduced, approximately as long as width at base (Fig. 19F); Hispaniola	***Cla. minisignaria***

#### *ochrochlaena* group

##### Claduncaria maestrana

Taxon classificationAnimaliaLepidopteraTortricidae

Razowski & Becker, 2010

9E3170E4-FD89-5630-A56D-FEA6B46354F1

**Figure 10.**
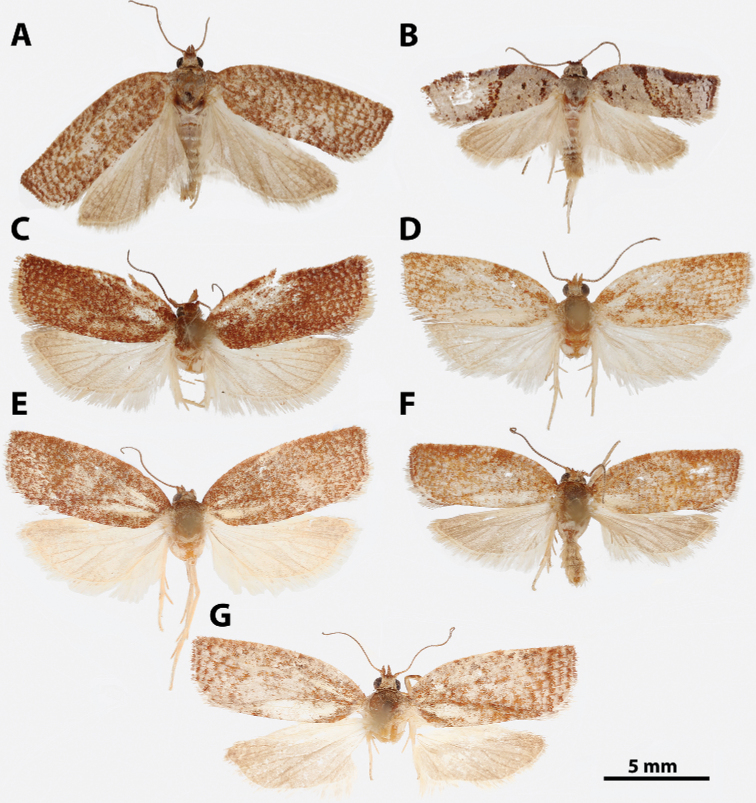
*Claduncaria ochrochlaena* group adults. **A***Cla. maestrana* ♀, Cuba (VBC) **B***Cla. maestrana*♂, Cuba (VBC) **C***Cla. ochrochlaena* ♀, Dominican Republic (CMNH) **D***Cla. ochrochlaena* holotype ♂, Dominican Republic (CMNH) **E***Cla. rawlinsana* sp. nov. paratype ♀, Dominican Republic (CMNH) **F***Cla. rawlinsana* sp. nov. holotype ♂, Dominican Republic (CMNH) **G***Cla. praedictana* sp. nov. holotype ♀, Dominican Republic (CMNH).

Figs 10A, B , 18A , 19A , 25B

Claduncaria maestrana Razowski & Becker, 2010: 11
Clepsis labisclera Razowski & Becker, 2010: 20, syn. nov.

###### Diagnosis.

**Figure 11.**
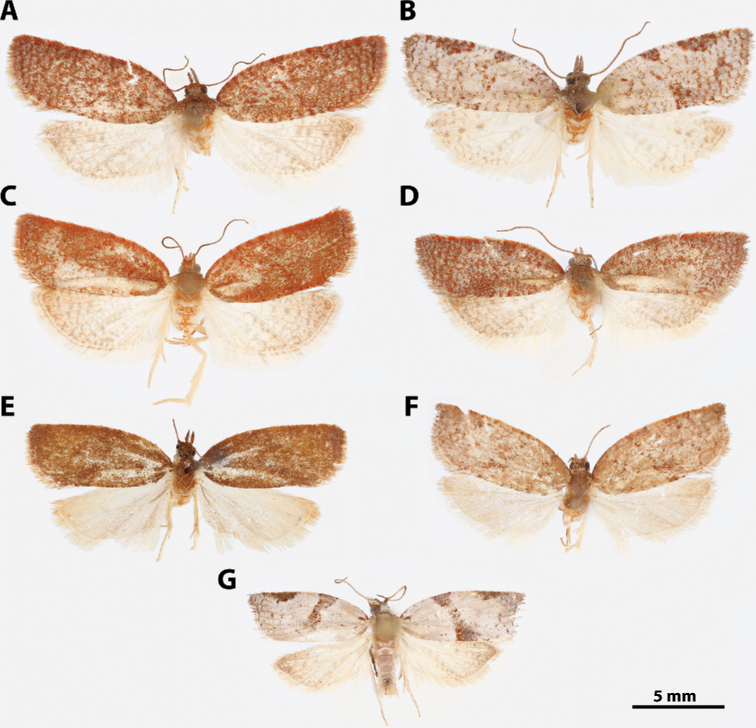
*Claduncariamesosignaria* group adults. **A***Cla. mesosignaria* holotype ♀, Dominican Republic (CMNH) **B***Cla. mesosignaria* ♂ (holotype of *Argyrotaeniathamaluncus* syn. nov.), Dominican Republic (CMNH) **C***Cla. minisignaria* holotype ♀, Dominican Republic (CMNH) **D***Cla. minisignaria* ♂, Dominican Republic (CMNH) **E***Cla. chalarostium* comb. nov., stat. nov. holotype ♀ (erroneously affixed with *Argyrotaenia jamaicana* paratype label), Jamaica (CMNH) **F***Cla. rufochlaena* holotype ♂, Jamaica (CMNH) **G***Cla. taino* sp. nov. holotype ♂, Dominican Republic (CMNH).

Males of *Claduncaria maestrana* (Fig. 10B) are most similar to males of *Cla. taino* (Fig. 11G) from Hispaniola. They can be easily separated by the shape of the uncus: divergently bifurcate in *Cla. maestrana* (Fig. 18A) and apically broadened in *Cla. taino* (Fig. 18G). Females (Fig. 10A) are most similar to *Cla. praedictana* (Fig. 10G) from Hispaniola, from which they can be separated by possessing relatively narrower ventroposterior grooves on the papillae anales (Fig. 19A) compared to *Cla. praedictana* (Fig. 19D).

###### Type material.

***Claduncaria maestrana***: ***Holotype*** ♂: **Cuba**: **S[an]t[ia]go [de Cuba**]: Sierra Maestra, P[ico] Cuba, 1500 m, 31 vii 1990, 73582 [photograph examined], genitalia slide #015 [figure examined] (VBC, see remarks below). ***Paratypes*** (3♂♂): **Cuba: Holguín**: Pin[ares de] Mayarí, 640 m, viii 1990, 72022 [not examined] (VBC, see remarks below). ***Clepsis labisclera***: ***Holotype*** ♀: **Cuba**: **S[an]t[ia]go [de Cuba**]: Sier[ra] Maestra, 1500 m, 31 vii 1990, 73583 [photograph examined], genitalia slide number not listed [figure examined] (VBC, see remarks below). ***Paratype*** (♀): **Cuba**: same data as holotype [not examined] (VBC, see remarks below).

###### Additional material examined.

(11♂♂, 2♀♀) **Cuba: S[an]t[ia]go [de Cuba**]: 7♂♂, 2♀♀, same data as *Claduncaria maestrana* holotype (1♂ KAA_DNA_0039); KAA diss. #0150 (♂); #0153 (♀); #0154(♀), KAA_DNA_0040 (VBC). **Holguín**: 2♂♂, same data as *Claduncaria maestrana* paratypes except vii 1990. KAA diss. #0152 (VBC). **S[an]t[ia]go**: 2♂♂, Gran Piedra, 20 vii 1990. KAA diss. #0151 (VBC).

###### Redescription.

**Male (n = 11). *Head*.** Scales on vertex pale brown, occasionally with a few brick red scales, usually concentrated anteriorly. Scales on frons brick red to deep blood red. Lateral surface of labial palpus concolorous with scales on frons, second segment expanded apically. Medial surface of labial palpus pale yellow. Scape concolorous with scales on frons, sometimes slightly darker. Sensillae approximately width of flagellomere, lightly curved; dorsal scales of flagellomere alternating between dark brown basal row and golden apical row. ***Thorax*.** Scales on dorsum of pro- and mesothorax concolorous with vertex. Lateral surface of foreleg with red-orange scales on coxa and femur; tibia and tarsus dark brown. Lateral surface of midleg light with red-orange and straw yellow scales, tarsi pale brown. Lateral surface of hindleg straw yellow, tarsi pale brown. Dorsal surface of forewing (Fig. 10B) with basal fascia, median fascia, and subapical blotch light red-orange, brick red, or dark brown, often most distinct along costa; antemedian and postmedian interfasciae ashy gray with faint red-orange reticulations present, usually with a pair of distinct red-orange dots present in antemedian interfascia; fringe with short scales dark red-brown, off-white at tornus; longer scales pale gray-brown; FWL 5.5–7.0 mm (mean = 6.2; n = 11). Dorsal surface of hindwing uniformly pale brown, with darker scales in outer half especially along veins, strigulae absent; fringe concolorous, including darker scales at apex. Ventral surface of forewing pale brown, pale red-orange along costa. Ventral surface of hindwing concolorous except apically where there is a defined pale base of short dark scales amongst longer pale scales. ***Abdomen*.** Vestiture straw yellow to pale brown. Genitalia (Fig. 18A) with uncus divergently bifurcate, broad at apex, smoothly rounded on anterior edge, quadrate on posterior edge with small ridge present, apicoventral setae minute; socii present as a small setose bump; arms of gnathos joined apically, vertically bifid, with both apices globose; terminal plate vertically bifurcate at apex; tegumen massive, robust; transtilla with small lateral processes, complete, unadorned; valvae semicircular, with dense patch of thin, deciduous setae present at base; sacculus to 0.75 ×; juxta with moderate notch, short setae present on lateral lobes; phallus pistol-shaped, slightly curved, sharply acute at apex, caulis moderate, sharp; cornuti thin, straight, approximately 0.25 × length of phallus.

**Female (n = 2). *Head*.** As in male but scaling on vertex pale red-orange; sensillae approximately 0.5 × width of flagellomere. ***Thorax*.** Scales on dorsum of pro- and mesothorax as in male, but with more extensive red-orange scaling. Legs as in male. Dorsal surface of forewing (Fig. 10A) red-orange or brick red, banding obscure; fringe brick red, off-white at tornus; FWL 8.5–9.0 mm (mean = 8.8; n = 2). Dorsal surface of hindwing pale brown, fringe as in male; strigulae distinct in one specimen examined; ventral surface of forewing as in male; ventral surface of hindwing as in male but with strigulae apparent. ***Abdomen*.** Vestiture pale brown. Genitalia (Fig. 19A) with papillae anales notched laterally, evenly roughened on anterior portion, only sparsely roughened on posterior portion with broad groove; apophyses posteriores approximately 0.5 × length of sternum VII; apophyses anteriores approximately 0.67 × length of sternum VII; sterigma heavily sclerotized, quadrate; antrum lightly sclerotized, colliculum present as tube-shaped structure; ductus bursae long, widening gradually anteriorly; ductus seminalis arising at approximately 0.2 × length of ductus bursae; signum short, straight; capitulum absent.

###### Distribution.

*Claduncaria maestrana* is known from three widely separated localities in the Sierra Maestra range of southeastern Cuba (Fig. 25B). This appears to be the most widely distributed species of *Claduncaria*; all other species are known from single mountain peaks or a series of closely-situated peaks. Collection localities range from 640 to 1500 m elevation.

###### Ecology.

Nothing is known of the biology of *Cla. maestrana*. Capture dates of examined specimens are from July and August.

###### Remarks.

The holotypes of *Clepsis labisclera* Razowski & Becker and *Claduncaria maestrana* Razowski & Becker were collected from the same locality on the same date. The female genitalia are not like those of any known *Clepsis*, but they fit well with our revised concept of *Claduncaria*. Razowski may have placed it in *Clepsis* because females of *Claduncaria* were previously unknown. The notched papillae anales, heavily sclerotized, quadrate sterigma, and reduced signum corroborate that *Clepsis labisclera* Razowski & Becker is the female of *Claduncaria maestrana* Razowski & Becker. Razowski listed the paratypes as having been collected in August, but we suspect the label was erroneously transcribed. We examined two males with identical VBC accession numbers and labels, but with “vii” instead of “viii.” The holotype and paratype of *Cle. labisclera*, as well as the holotype of *Cla. maestrana* were found in ISEZ, not VBC as listed in Razowski and Becker (2010). The remaining male paratypes of *Cla. maestrana* are probably in ISEZ as well.

Two specimens of *Cla. maestrana* were submitted for barcoding. Unfortunately, one failed completely and the other provided only an incomplete sequence (280 bp), so we were unable to include it in either analysis (Figs 3, 4).

##### Claduncaria ochrochlaena

Taxon classificationAnimaliaLepidopteraTortricidae

(Razowski, 1999)

48BCBFA6-6FE8-5DC6-975D-118B297E6957

Figs 10C, D , 18B , 19B , 25B

Cladotaenia ochrochlaena Razowski, 1999: 312

###### Diagnosis.

The combination of a divergently bifurcate uncus with smooth, rounded apices and an irregular vertically bifurcate terminal plate of the gnathos in the male genitalia (Fig. 18B) distinguish *Cla. ochrochlaena* from all other members of the genus. Female genitalia (Fig. 19B) have the following unique combination of features: a narrow groove in the ventroposterior portion of the papillae anales and the complete absence of a signum.

###### Type material.

***Holotype*** ♂: **Dominican Republic: Pedernales**: 5 km NE Los Arroyos, 18°15'N, 71°45'W, 1680 m, 28 vii 1990, C.W. Young, J.E. Rawlins, S. Thompson [examined], Razowski genitalia slide #10699 [examined] (CMNH).

###### Additional material examined.

(3♂♂, 1♀) **Dominican Republic**: **Independencia**: 2♂♂ [one with abdomen missing], Sierra de Bahoruco, north slope, 18°41'31"N, 71°35'35"W [18°17'30"N, 71°43'08"W], 2116 m, 8 xi 2002, broadleaf forest with pine, W.A. Zanol, C. W. Young, C. Staresinic, J. Rawlins. KAA diss. #0120, KAA_DNA_0043 (CMNH). **Pedernales**: 1♂, same data as holotype except with 30 ix 1991, cloud forest, J. Rawlins, R. Davidson, C. Young, S. Thompson, J. Rawlins. 1♀, same data as holotype except 20 x 1991, cloud forest, J. Rawlins, R. Davidson, C. Young, S. Thompson, KAA diss. #0126, KAA_DNA_0044 (CMNH).

###### Redescription.

**Male (n = 4). *Head*.** Scales on vertex and frons red-orange to mahogany red. Labial palpus with lateral surface of all three segments light red-orange, medial surface pale yellow. Scape brick red to mahogany red with a few straw yellow scales. Dorsal scales of flagellum with first few segments with alternating rows of straw yellow and red-orange scales, red-orange scales becoming dark brown after first few segments. Sensillae 0.5–0.75 × width of flagellomere, only slightly recurved. ***Thorax*.** Dorsum of pro- and mesothorax light red-orange to warm brown; tegulae concolorous. Lateral surface of forelegs light red-orange, tarsi dark brown; lateral surface of midlegs straw yellow, tarsi dark brown; lateral surface of hindlegs pale yellow to white. Medial surface of legs pale yellow to white. Dorsal surface of forewing (Fig. 10D) ochraceous red, overlaid by a thin network of white reticulations; median fascia and subapical blotch visible along costa, brick red; fringe with short scales pale red-orange, long scale off-white to pale brown; FWL 7.0–8.0 mm (mean = 7.5; n = 4). Dorsal surface of hindwing white, light brown shading present towards apex; fringe off-white, pale brown at extreme apex. Ventral surface of forewing warm brown. Ventral surface of hindwing white. ***Abdomen*.** Vestiture warm brown. Genitalia (Fig. 18B) with uncus divergently bifurcate, moderate in width, smoothly rounded apically, apicoventral setae not observed, but sockets present at apices; socii present as small nub with projecting setae; arms of gnathos robust, slightly irregular, smooth; terminal plate irregularly bifurcate; tegumen massive, robust, swollen anteriorly, unmodified; transtilla with lateral processes, complete, unadorned; valvae somewhat triangular but with rounded apex, with dense patch of long, thin, deciduous setae at base; sacculus to 0.8 ×; juxta with shallow notch, sockets present on lateral lobes; phallus pistol-shaped, abruptly angled, sharply acute at apex, caulis prominent; two cornuti present in each of two specimens examined (including holotype): thin, straight, approximately 0.33 × length of phallus.

###### Description.

**Female (n = 1). *Head*.** As in male except vertex, frons, and lateral surface of palpus entirely brick red. Sensillae short, no more than 0.25 × width of flagellomere. ***Thorax*.** As in male but dorsum of pro- and mesothorax and tegulae entirely brick red. Dorsal surface of forewing (Fig. 10C) with costa subtly concave along distal third; uniformly red with fine network of brick red reticulations; fringe with short scales concolorous with ground color of forewing, long scales pale yellow to off-white; FWL 7.5 mm. Dorsal surface of hindwing as in male but fringe with short scales pale brown, longer scales off-white. Frenulum with three bristles. ***Abdomen*.** Vestiture unknown. Genitalia (Fig. 19B) with papillae anales notched laterally, evenly roughened except for narrow groove on ventroposterior portion; apophyses posteriores approximately 0.5 × length of sternum VII; apophyses anteriores approximately 0.5 × length of sternum VII, slightly kinked; sterigma heavily sclerotized, shallow; antrum lightly sclerotized; colliculum present as short tube-shaped structure; ductus bursae long, of almost uniform width throughout; ductus seminalis arising at approximately 0.2 × length of ductus bursae; corpus bursae small; signum, capitulum absent.

###### Distribution.

*Claduncaria ochrochlaena* is known from two localities in the Dominican Republic in the Sierra de Bahoruco near the Haitian border (Fig. 25B). It is expected to occur in neighboring regions of Haiti. It is sympatric with *Cla. minisignaria*.

###### Biology.

Nothing is known of the biology of *Cla. ochrochlaena*. Examined specimens were collected from July to November.

###### Remarks.

The above represents the first description of the female of *Cla. ochrochlaena*. There is a discrepancy in the label data of two male specimens from Independencia. The label reads “Sierra de Bahoruco” but the coordinates are for the Sierra de Neiba. After comparing coordinates from specimens collected the previous night and discussing the situation with John Rawlins (CMNH), we interpret the coordinates to be incorrect. Dr. John Rawlins kindly supplied us with the correct coordinates. COI sequences for two barcoded specimens of *Cla. ochrochlaena* were identical

##### Claduncaria rawlinsana sp. nov.

Taxon classificationAnimaliaLepidopteraTortricidae

AE7C17DA-36B7-53B4-892C-4E3A2A5F57C3

http://zoobank.org/96CD1EC6-5AB3-4621-95F9-987F7FDDF56D

Figs 10E, F , 18C , 19C , 25B

###### Diagnosis.

*Claduncaria rawlinsana* (Fig. 10E, F) is most likely to be confused with *Cla. ochrochlaena* (Fig. 10C, D). Both occur in the Sierra de Bahoruco of Hispaniola, but do not appear to be sympatric (Fig. 25B). Male genitalia of *Cla. rawlinsana* (Fig. 18C) can be separated from those of *Cla. ochrochlaena* (Fig. 18B) by possessing uncus arms with quadrate apices, which are rounded in *Cla. ochrochlaena*. Female genitalia differ in the presence of a signum in the corpus bursae (Fig. 19C), which is absent in *Cla. ochrochlaena* (Fig. 19B). Females could also be confused with those of *Cla. praedictana* (Fig. 19D), but can be distinguished by the narrower ventroposterior grooves of the papillae anales compared to that species, in addition to their disjunct ranges (Fig. 25B).

###### Type material.

***Holotype*** ♂: **Dominican Republic: Pedernales**: Sierra de Ba[h]oruco, Aceitillar, 25.2 km ENE Pedernales, 18°05'29"N, 71°31'16"W, 1272 m, 14 vi 2003, dense broadleaf forest, pine, C. Young, J. Rawlins, C. Nunez, R. Davidson, P. Acevedo, M. de la Cruz. HOLOTYPE *Claduncaria rawlinsana* Austin & Dombroskie [typed red label] (CMNH). ***Paratypes*** (1♂, 1♀): **Dominican Republic: Pedernales**: 1♂, same data as holotype, KAA diss. #0121, KAA_DNA_0047 (CUIC). 1♀, 37 km N Cabo Rojo, 1480 m, 18°09'N, 71°35'W, 19 x 1991, grassland with pines, J. Rawlins, R. Davidson, C. Young, S. Thompson, KAA diss. #0122, KAA_DNA_0048 (CMNH). All paratypes affixed with the following typed blue label: PARATYPE ♂/♀ *Claduncaria rawlinsana* Austin & Dombroskie, 2020.

###### Description.

**Male (n = 2). *Head.***

Scales on vertex and frons ochraceous red to brick red. Labial palpus with lateral surface of all three segments light red-orange, medial surface pale yellow. Scape brick red to straw yellow. Dorsal scales of flagellum with first few segments with alternating rows of straw yellow and ochraceous red, ochraceous red scales becoming dark brown after first few segments. Sensillae 0.50–0.75 × width of flagellomere, porrect. ***Thorax*.** Dorsum of pro- and mesothorax light red-orange intermixed with warm brown and white scales; tegulae concolorous. Legs similar to *Cla. ochrochlaena*. Dorsal surface of forewing (Fig. 10F) and ventral surfaces of both wings identical to *Cla. ochrochlaena*, but with banding slightly more distinct, fringe more salmon pink under high magnification; FWL 6.5–7.0 (mean = 6.8; n = 2). Dorsal surface of hindwing similar to *Cla. ochrochlaena* but with more extensive light brown shading. ***Abdomen*.** Vestiture with pale yellow to white scaling. Genitalia (Fig. 18C) with uncus divergently bifurcate, quadrate at apices; apicoventral setae minute, projecting from apices; socii present at small nubs with setae projecting; arms of gnathos robust, smooth; terminal plate vertically bifurcate at apex, both apices sharpened; tegumen massive, robust; transtilla with small pointed lateral processes, complete, unadorned; valvae nearly triangular, rounded at apex, with dense patch of long, thin, deciduous setae at base; sacculus to 0.75 ×; juxta with shallow notch, short setae present on lateral lobes; phallus pistol-shaped, downcurved, sharply acute at apex, caulis moderate; two cornuti observed in one specimen examined: thin, straight, approximately 0.2 × length of phallus.

**Female (n = 1). *Head*.** As in male except with vertex, frons, palpi, and flagellomeres with more extensive brick red scaling. Sensillae short, porrect, no more than 0.25 × width of flagellomere. ***Thorax*.** As in male except with more extensive brick red scaling on dorsum of pro- and mesothorax as well as tegulae. Dorsal surface of forewing (Fig. 10E) similar to *Cla. ochrochlaena* but lacking subtle subapical concavity of *Cla. ochrochlaena* and with slightly more ochreous and less brick red scaling; fringe as in male, but with less extensive salmon pink scaling; FWL 8.0 mm. Dorsal surface of hindwing almost uniformly white, only faint pale brown shading near apex. Ventral surface of both wings similar to *Cla. ochrochlaena*. Frenulum with three bristles. ***Abdomen*.** Vestiture unknown. Genitalia (Fig. 19C) with papillae anales notched laterally, evenly roughened except for moderate groove on ventroposterior portion; apophyses posteriores approximately 0.5 × length of sternum VII; apophyses anteriores approximately 0.67 × length of sternum VII; sterigma heavily sclerotized, deep, wide, quadrate; antrum lightly sclerotized; colliculum present as narrow ring; ductus bursae long, of almost uniform width throughout; ductus seminalis arising at approximately 0.2 × length of ductus bursae; corpus bursae small; signum short, straight; capitulum absent.

###### Etymology.

We take great pleasure in naming this species after Dr. John E. Rawlins, curator emeritus of the Section of Invertebrate Zoology at the Carnegie Museum of Natural History, who led numerous entomological expeditions to the Dominican Republic and collected the vast majority of all specimens examined for this study.

###### Distribution.

*Claduncaria rawlinsana* is known from two localities in the Dominican Republic in the eastern end of the Sierra de Bahoruco (Fig. 25B). Collection localities range from 1272 to 1480 m elevation.

###### Ecology.

Nothing is known of the biology of *Cla. rawlinsana*. Capture date of examined specimens are from June and October.

###### Remarks.

See the remarks under *Cla. praedictana* regarding this species’ relationship to it. COI sequences between two barcoded specimens of *Cla. rawlinsana* were identical.

##### Claduncaria praedictana sp. nov.

Taxon classificationAnimaliaLepidopteraTortricidae

029E0B1D-1504-573A-A6A0-7B363904A3C0

http://zoobank.org/72F3C08A-2D4F-4D40-8F7D-552483C93B17

Figs 10G , 19D , 25B

###### Diagnosis.

Females of *Cla. praedictana* (Fig. 9G) are most similar to females of *Cla. maestrana* (Fig. 9A). See the diagnosis under that species. Males are unknown (but see remarks below).

###### Type material.

***Holotype*** ♀: **Dominican Republic: Monseñor Nouel**: 1 km E Paso Alto de Casabito, 7 km NW La Ceiba, 1130 m, 19°02'N, 70°29'W, 28 vii 1992, cloud forest, R. Davidson, J. Rawlins, S. Thompson, C. Young; KAA diss. #0123; KAA_DNA_0042. HOLOTYPE *Claduncaria praedictana* Austin & Dombroskie [typed red label] (CMNH).

###### Description.

**Male.** Male unknown.

**Female (n = 1). *Head*.** Scales on vertex white and warm brown, blood red anteriorly. Scales on frons red-orange. Labial palpus with lateral surface entirely red-orange, medial surface pale yellow. Slight purple iridescence present on lateral surface of palpus, visible at certain angles. Scape straw yellow with a few blood red scales. Dorsal scales of flagellum with alternating rows of warm brown and straw yellow, many missing. Sensillae short, straight, no more than 0.5 × width of flagellomere. ***Thorax*.** Dorsum of pro- and mesothorax warm brown with a few red-orange scales. Metathorax missing (see remarks below), but presumably typical of genus. Tegulae concolorous with dorsum of pro- and mesothorax. Foreleg with many scales missing, but apparently light red-orange on lateral surface, tarsi warm brown; midlegs similar; hindlegs pale yellow to white on lateral surface. Medial surface of legs pale yellow to white. Dorsal surface of forewing (Fig. 10G) heavily worn, but apparently light red-orange with dark brown reticulations; banding faint; fringe damaged, but appears to have short scales pale gray-brown, intermixed with brick red scales at apex and replaced with off-white scales at tornus; FWL 8.0 mm. Dorsal surface of hindwing white with heavy brown shading towards apex, no strigulae; fringe with short scales pale brown along entire margin, long scales off-white. Ventral surface of forewing warm brown, costa straw yellow with light red-orange spots. Ventral surface of hindwing white with light brown strigulae at apex. Frenulum with at least two bristles. ***Abdomen*.** Vestiture unknown. Genitalia (Fig. 19D) with papillae anales notched laterally, evenly roughened, except for broad groove occupying most of swollen ventroposterior portion; apophyses posteriores approximately 0.5 × length of sternum VII; apophyses anteriores approximately 0.5 × length of sternum VII, curved; sterigma heavily sclerotized, broad, quadrate; antrum lightly sclerotized; colliculum present as short tube-shaped structure; ductus bursae long, widening gradually anteriorly; ductus seminalis arising at approximately 0.2 × length of ductus bursae; corpus bursae small; signum short, straight; capitulum absent.

###### Etymology.

The specific epithet *praedictana*, from *praedictus* (Latin), refers to the hypothesized structure of the yet unknown male genitalia (but see remarks below).

###### Distribution.

At present, *Cla. praedictana* is only known from the vicinity of Loma del Casabito in the Cordillera Central of the Dominican Republic at an elevation of 1130 m (Fig. 25B).

###### Biology.

Nothing is known of the biology of *Cla. praedictana*. The holotype was collected in July.

###### Remarks.

The hindwings and metathorax of the holotype broke off when removing the abdomen for dissection. The hindwings were carefully reattached before photographing, but unfortunately the metathorax was lost.

Despite the close proximity (< 4 km) of the type localities of *Cla. praedictana* and *Cla. taino* (known only from males), we do not believe the two species are conspecific. Based on the genitalia, *Cla. praedictana* is a member of the *ochrochlaena* group, whereas *Cla. taino* is a member of the *mesosignaria* group. We predict that the yet-to-be-discovered males of *Cla. praedictana* will have a strongly divergent bifid uncus, similar to that of *Cla. maestrana*. Further, a partial DNA barcode was recovered for the holotype of *Cla. praedictana* (563 bp) and a complete DNA barcode for a paratype of *Cla. taino*, and sequence divergence was 11.1%.

Our Maximum Likelihood analysis (Fig. 4) suggests that *Cla. praedictana* may be sister to *Cla. rawlinsana*. Minimum sequence divergence between these two species was 0.9%. Differences in the width of the ventroposterior groove of the papillae anales and patterns of distribution in *Claduncaria* support *Cla. praedictana* as being distinct from *Cla. rawlinsana*.

#### *mesosignaria* group

##### Claduncaria mesosignaria

Taxon classificationAnimaliaLepidopteraTortricidae

(Razowski, 1999) comb. nov.

B21BF388-358C-5F16-821D-08A9BF57180E

Figs 11A, B , 18E , 19G , 25A

Argyrotaenia mesosignaria Razowski, 1999: 311
Argyrotaenia thamaluncus Razowski, 1999: 311, syn. nov.
Clepsis mesosignaria error in figure of Razowski & Becker, 2010: 37

###### Diagnosis.

*Claduncaria mesosignaria* (Fig. 11A, B) is most similar to *Cla. minisignaria* (Fig. 11C, D). *Claduncaria mesosignaria* is a markedly sexually dimorphic species, whereas *Cla. minisignaria* is not. Male genitalia of *Cla. mesosignaria* (Fig. 18E) are distinct from those of *Cla. minisignaria* (Fig. 18F) in possessing a broader apex of the uncus and a completely straight phallus. Female genitalia can be separated from those of *Cla. minisignaria* by the presence of a moderate signum (Fig. 19G), which is much shorter in *Cla. minisignaria* (Fig. 19F).

###### Type material.

***Argyrotaenia mesosignaria***: ***Holotype*** ♀: **Dominican Republic**: La Vega: 9 km SE Constanza, near Valle Nuevo, 18°50'N, 70°42'W, 1930 m, 17 viii 1990, J.E. Rawlins, S. Thompson [examined], Razowski genitalia slide #10702 [examined] (CMNH). ***Argyrotaenia thamaluncus***: ***Holotype*** ♂: Dominican Republic: Peravia [San José de Ocoa]: 3 km SW La Nuez, upper Rio Las Cuevas, 18°40'N, 70°36'W, 1850 m, 5–6 viii 1990, J. Rawlins, S. Thompson [examined], Razowski genitalia slide #10704 [examined] (CMNH).

###### Additional material examined.

(3♂♂, 4♀♀) **Dominican Republic: Peravia [San José de Ocoa**]: 2♂♂, 2♀♀, 3 km SW La Nuez, upper Rio Las Cuevas, 1880 m, 18°39'N, 70°36'W, 5–6 x 1991, J. Rawlins, R. Davidson, C. Young, S. Thompson, cloud forest on river (1♂, 1♀ CUIC; remainder CMNH). KAA diss. #0107(♀), KAA_DNA_0037 (CMNH); #0108(♀, CUIC); #0111(♂), KAA_DNA_0038 (CMNH). 1♀, same as previous except 2 ix 1995, J. Rawlins, G. Onore, R. Davidson, KAA diss. #0110 (CMNH). **La Vega**: 1♂, 2.3 km SE Constanza, 18°45'N, 70°37'W, 2225 m, 24–25 xi 1992, R. Davidson, M. Klingler, S. Thompson, J. Rawlins, grassland with pines and scattered marshes, KAA diss. #0112 (CMNH). 1♀, Cordillera Central, Valle Nuevo Station, 5.2 km ESE Valle Nuevo, 18°46'40"N, 70°38'26"W, 2288 m, 23 v 2003, C. Young, J. Rawlins, C. Nunez, R. Davidson, P. Acevedo, open pine forest on slope, KAA diss. #0109 (CMNH).

###### Redescription.

**Male (n = 4). *Head*.** Scales on vertex and frons pale brown. Lateral surface of labial palpus with first segment mahogany red-orange, second segment red-orange on basal half and pale brown on apical half, third segment pale brown, white at extreme apex. Labial palpus with remarkable iridescent purple and green coloration when viewed at certain angles. Medial surface of labial palpus pale yellow. Scape light brown with occasional mahogany red scales. Sensillae approximately 1.25 × width of flagellomere, lightly curved; dorsal scales of flagellomere dark brown with bases golden. ***Thorax*.** Scales on dorsum of pro- and mesothorax concolorous with vertex. Foreleg dark brown with red-orange scales present on coxa and femur; midleg dark brown to light brown; hindleg pale yellow with tibial spurs and tarsi pale brown. Dorsal surface of forewing (Fig. 11B) with antemedian and postmedian interfasciae ashy gray to pale brown, nearly white in some individuals; basal fascia, median fascia, and postmedian fascia dark brown, most visible along costa, sometimes fading to obsolescence near inner margin, mahogany red scales scattered throughout, but most dense along costa; fringe with short scales gray-brown, especially along apical half, becoming concolorous with ground color of forewing towards tornus but still with a few small patches of gray-brown scales or lone brick red scales; longer scales concolorous with shorter scales but without red; FWL 8.5–9.0 mm (mean = 8.8; n = 4). Dorsal surface of hindwing white but with dark brown strigulae, especially so near apex; concolorous with dorsal surface of hindwing, including darker scales at apex. Ventral surface of forewing warm brown, costa white with dark brown spots. Ventral surface of hindwing as on dorsal surface, but more distinctive strigulae. As on palpus, similar green-purple iridescence visible on ventral surfaces of wings from certain angles. ***Abdomen*.** Vestiture with first segment white, remaining segments warm brown, white scales present at tip of abdomen. Genitalia (Fig. 18E) with uncus robust at base, widening dramatically to broad apex, approximately as wide as tegumen, indented slightly medially, apicoventral setae moderate, projecting from lateral lobes; socii present as small nubs with projecting setae; arms of gnathos robust, minutely roughened on lateral surface; terminal plate long, smoothly rounded, with thin medial ridge; tegumen massive, robust, unmodified; transtilla with large lateral processes, complete, unadorned; valvae acute apically, nearly triangular, with dense patch of long, deciduous, paddle-like setae present near base; sacculus to 0.8 ×; juxta with moderate notch, short setae present on lateral lobes; phallus dagger-like, nearly completely straight, sharply acute at apex, caulis obsolete; two to four cornuti observed in three specimens examined (including holotype of *A. thamaluncus*): thin, straight, approximately 0.25 × length of phallus, deciduous (cornutus observed in ductus bursae of one female examined).

**Female (n = 5). *Head*.** As in male except scaling on vertex and frons brick red to red-orange, concolorous with scales on labial palpus. Sensillae short, porrect, no more than 0.5 × width of flagellomere. ***Thorax*.** Dorsum of pro- and mesothorax as in male but with more extensive brick red or red-orange scaling. Dorsal surface of forewing (Fig. 11A) almost uniformly brick red, heavily reticulated; median fascia and subapical blotch only faintly discernable as a slightly darker shade of red; fringe with short scales chalky purple-gray, longer scales pale orange-yellow; FWL 8.5–10.5 mm (mean = 9.4; n = 5). One individual with a more red-orange hue to the forewings, making banding more apparent. Under magnification this individual with dark brown scaling on median fascia and subapical blotch as in males. Frenulum with three bristles. ***Abdomen*.** Vestiture as in male but with brick red to red-orange scaling. Genitalia (Fig. 19G) with papillae anales massive, laterally rounded and apically slightly swollen, evenly roughened on ventral surface; apophyses posteriores approximately 0.5 × length of sternum VII; apophyses anteriores approximately 0.67 × length of sternum VII; sterigma heavily sclerotized, quadrate; antrum lightly sclerotized, colliculum present as tube-shaped structure; ductus bursae widening gradually anteriorly; ductus seminalis arising at approximately 0.2 × length of ductus bursae; corpus bursae not much wider than widest portion of ductus bursae, thus obscuring junction of corpus and ductus; signum short, straight; capitulum absent.

###### Distribution.

*Claduncaria mesosignaria* is known from the Cordillera Central in the Dominican Republic in the provinces of La Vega and San José de Ocoa. It appears to be highly restricted in its distribution (Fig. 25A). Collection localities range from 1850 to 2288 m elevation.

###### Ecology.

Nothing is known of the biology of *Cla. mesosignaria*. Captures dates of examined specimens range from May to November.

###### Remarks.

Because of the similarity of the male genitalia of *Argyrotaenia thamaluncus* to those of *Argyrotaenia minisignaria* (see remarks under *Cla. minisignaria*), the identical data labels many of the specimens possess, and only 0.54% COI sequence divergence between a barcoded male and female, there is sufficient evidence to support *A. thamaluncus* as the previously unknown male of *Argyrotaenia mesosignaria*.

Because both species were described in the same paper, one name does not have priority over the other. We opt to preserve *A. mesosignaria* and treat *A. thamaluncus* as a junior synonym to reduce potential confusion and to ensure the holotype of *A. mesosignaria* and *A. minisignaria* is of the same sex. Despite lacking a bifid uncus, the presence of small setose nub-like socii, a robust, well-sclerotized tegumen, a transtilla with lateral processes, and pointed valvae, place both *A. mesosignaria* and *A. minisignaria* in *Claduncaria*.

Our Maximum Likelihood analysis (Fig. 4) strongly support the monophyly of *A. mesosignaria* + *A. minisignaria*. Minimum COI sequence divergence between the two species was 5.3%.

##### Claduncaria minisignaria

Taxon classificationAnimaliaLepidopteraTortricidae

(Razowski, 1999) comb. nov.

F974338E-5D1B-5C7F-A20A-4212C04F831E

Figs 11C, D , 18F , 19F , 25A

Argyrotaenia minisignaria Razowski, 1999: 311

###### Diagnosis.

*Claduncaria minisignaria* (Fig. 11C, D) is most similar to *Cla. mesosignaria* (Fig. 11A, B). See diagnosis for that species.

###### Type material.

***Holotype*** ♀: **Dominican Republic: Pedernales**: 8 km NE Los Arroyos, 18°16'N, 71°44'W, 1940 m, 14 vii 1990, J. Rawlins, C.W. Young, S.A. Thompson [examined], Razowski genitalia slide #10700 [examined] (CMNH). ***Paratype*** (♀): same as previous [examined], Razowski genitalia slide #10701 [not examined], KAA_DNA_0045 (CMNH).

###### Additional material examined.

(1♂) **Dominican Republic**: 1♂, same data as holotype [examined], Razowski genitalia slide #10703 [examined], KAA_DNA_0046 (CMNH).

###### Description.

**Male (n = 1). *Head*.** Scales on vertex warm brown with row of red-orange scales anteriorly. Scales on frons red-orange with shorter light brown scales present ventrally. Labial palpus with lateral surface entirely red-orange, with exception of apical tip of third segment, which is white. Lateral surface of labial palpus with iridescent purple coloration faintly visible at certain angles, but not as dramatic as in *Cla. mesosignaria*. Medial surface of labial palpus pale yellow. Scape red-orange intermixed with a few dark brown scales. Dorsal scales of flagellum dark brown with bases golden. ***Thorax*.** Scales on dorsum of pro- and mesothorax dark brown. Tegulae concolorous with pro- and mesothorax but with a few pale brown scales at apex. Forelegs with ventral surface red-orange with a few dark brown scales on tarsi; midlegs missing; hindlegs with ventral surface red-orange, tarsi missing; medial surface pale yellow. Dorsal surface of forewing (Fig. 11D) brick red, but red scaling only visible under magnification, heavily suffused with warm brown scales with darker reticulations, causing moth to appear almost uniformly brown; banding faint; fringe predominantly gray-brown, long scales intermittently brick red or red-orange; FWL 8.0 mm. Dorsal surface of hindwing white with heavy warm brown shading and strigulae towards apex; fringe off-white, becoming darker towards apex. Ventral surface of forewing warm brown; costa pale yellow with red-orange spots. Ventral surface of hindwing white with less extensive brown shading. ***Abdomen*.** Vestiture unknown. Genitalia (Fig. 18F) with uncus robust at base, widening in apical half to broad apex, almost as wide as tegumen, nearly flat apically, apicoventral setae moderate, projecting from lateral lobes; socii present as a small nub with projecting setae; arms of gnathos robust, minutely roughened on lateral surface; terminal plate long, smoothly rounded, with thin medial ridge; tegumen large, robust, unmodified; transtilla with lateral processes, but difficult to see, complete, unadorned; valvae acute apically, slightly elongate, nearly triangular, with dense patch of long, deciduous, paddle-like setae present near base; sacculus to 0.8 ×; juxta with deep notch, short setae present on lateral lobes; phallus pistol-shaped, gently curved, sharply acute at apex, caulis minute; two cornuti observed: thin, straight, approximately 0.5 × length of phallus.

###### Redescription.

**Female (n = 2). *Head*.** As in male except vertex and frons entirely red-orange. Labial palpus entirely red-orange. Scape entirely red-orange. Dorsal scales of flagellum red-orange with golden bases, becoming dark brown at approximately 0.33 × length of antenna. ***Thorax*.** As in male except dorsum of pro- and mesothorax with more extensive red-orange scaling. Legs as in male but with no brown scales on tarsi; midlegs similar to coloration on forelegs. Dorsal surface of forewing (Fig. 11C) red-orange and salmon pink under magnification, but more brick red without magnification; banding faint to obsolete; light red-orange; fringe almost entirely red-orange, longer scales pale orange towards tornus; FWL 8.5–10.0 mm (mean = 9.3; n = 2). Frenulum with three bristles. ***Abdomen*.** Vestiture unknown. Genitalia (Fig. 19F) identical to those of *Claduncaria mesosignaria*, except lateral lobes of sterigma rounded and signum reduced to a near sclerite. Sternum VII not present in holotype slide.

###### Distribution.

*Claduncaria minisignaria* is known from a single locality in the Dominican Republic in the Sierra de Bahoruco near the Haitian border (Fig. 25A). It is expected to occur in neighboring regions of Haiti as well. It is sympatric with *Cla. ochrochlaena*. The sole collection locality is at 1940 m elevation.

###### Biology.

Nothing is known of the biology of *Cla. minisignaria*. The three examined specimens were collected in July.

###### Remarks.

The above represents the first description of the male of *Cla. minisignaria*. Razowski determined the single known male of this species as *Argyrotaenia mesosignaria*, but did not include it in the original description of the species. Both the specimen and genitalia slide possess these determination labels. Because it is from the same night and location as the type series of *Cla. minisignaria*, there is no reason to believe they are not conspecific with that species. See remarks under *Cla. mesosignaria* regarding this species’ transferal to *Claduncaria* and its relationship to that species.

##### Claduncaria chalarostium

Taxon classificationAnimaliaLepidopteraTortricidae

(Razowski & Becker, 2000b), comb. nov. stat. nov.

040E4F94-0ACD-59F0-B267-CD94DBB33BD8

Figs 11E , 19E , 25A

Argyrotaenia minisignaria chalarostium Razowski & Becker, 2000b: 315

###### Diagnosis.

The female of *Claduncaria chalarostium* (Fig. 11E) possesses the following unique combination of features: genitalia (Fig. 19E) with papillae anales apically swollen without a ventroposterior groove and complete absence of a signum in the corpus bursae. Males are unknown.

###### Type material.

***Holotype*** ♀: **Jamaica**: Blue Mt. Peak, viii, Avinoff & Shoumatoff [examined], genitalia slide #12273 [examined], KAA_DNA_0036 (CMNH).

###### Description.

**Male.** Male unknown.

###### Redescription.

**Female (n = 1). *Head*.** Scales on vertex, frons, lateral surface of palpus brick red, medial surface pale yellow. Scape brick red. Dorsal scales of flagellum with alternating rows of brick red and pale yellow scales. Sensillae short, porrect, approximately 0.25 × width of flagellomere. ***Thorax*.** Dorsum of pro- and mesothorax brick red; tegulae concolorous. Lateral surface of forelegs brick red, tibia and tarsi dark brown, medial surface straw yellow; midlegs missing; hindlegs straw yellow. Dorsal surface of forewing (Fig. 11E) uniformly brick red, banding obsolete; fringe with short scales chalky purple-gray along apical half, pale red-orange along tornal half; long scales entirely pale red-orange; FWL 8.5 mm. Dorsal surface of hindwing uniformly pale yellow, slightly orange towards apex, some gray scaling on inner half; no strigulae apparent; fringe concolorous, including pale red-orange scales at apex. Ventral surface of forewing pale brown, light red-orange along costa and along fringe. Ventral surface of hindwing as on dorsal surface. ***Abdomen*.** Vestiture unknown. Genitalia (Fig. 19E) with papillae anales large, without obvious groove, but slightly indented semi-circular patch present on ventrolateral surface; apophyses anteriores short, approximately 0.25 × length of sternum VII, barely extending beyond papillae anales; apophyses posteriores short, approximately 0.33 × length of sternum VII; sterigma quadrate, heavily sclerotized; antrum lightly sclerotized; colliculum represented by a pair of lateral sclerites; ductus bursae moderate, widening gradually to corpus bursae; ductus seminalis arising at 0.2 × length of ductus bursae; corpus bursae moderate; signum, capitulum absent.

###### Distribution.

*Claduncaria chalarostium* is known from a single female collected on Blue Mountain Peak, the highest point of Jamaica, with an elevation of 2256 m (Fig. 25A).

###### Biology.

Nothing is known of the biology of *Cla. chalarostium*. The only specimen was collected in August (but see remarks below).

###### Remarks.

Initially, the holotype of *A. m. chalarostium* Razowski & Becker, 2000b could not be located in CMNH. It was eventually discovered as a mislabeled female paratype of *Argyrotaenia jamaicana* Razowski & Becker, 2000b, a species for which females were unknown at the time of description. The genitalia slide and data label on this “paratype” are identical to those illustrated and transcribed by Razowski and Becker (2000b). We placed an additional label beneath this specimen explaining this, but have left the *A. jamaicana* paratype label in place.

The collection data on the label of the holotype of *A. m. chalarostium* was identical to that on the label of *Cla. rufochlaena*, the date and month on the former had been subsequently crossed out, and “Aug.” had been written instead. We are uncertain when and why this was done, but interpret the handwritten date to be correct.

Our Maximum Likelihood analysis (Fig. 4) suggests that this species may belong to the *ochrochlaena* group, but the genitalia are more similar to members of the *mesosignaria* group. In the absence of more robust molecular sampling, we choose to include *Cla. chalarostium* in the *mesosignaria* group.

Based on morphology, *Cla. chalarostium* and *Cla. rufochlaena* appear to be members of the *mesosignaria* group. In addition, both are known only from single specimens from Blue Mountain Peak, the former a single female and the latter a single male, which could lead to the conclusion that they are male and female of the same species. However, partial DNA barcodes were recovered from the holotypes. After cutting and alignment, a sequence divergence of 5.9% was observed, so we maintain them as separate species pending the discovery of additional specimens.

If future research supports the synonymization of these two aforementioned taxa, it would set a new and unusual taxonomic precedent. Both taxa were described in different articles in the same journal, published on the same date. Thankfully, ICZN 24.1 clearly supports the priority of *Cla. rufochlaena*, as it was originally described as a full species, whereas *A. m. chalarostium* was described as a subspecies.

##### Claduncaria rufochlaena

Taxon classificationAnimaliaLepidopteraTortricidae

Razowski & Becker, 2000a

F6535661-77B3-56A5-8AC9-0CF2552AD45B

Figs 11F , 18D , 25A

Claduncaria rufochlaena Razowski & Becker, 2000a: 208

###### Diagnosis.

Males of *Claduncaria rufochlaena* are unique among described *Claduncaria* in possessing both a divergently bifurcate uncus and a smoothly rounded terminal plate of the gnathos without a vertical bifurcation (Fig. 18D). Females are unknown.

###### Type material.

***Holotype*** ♂: **Jamaica**: Blue Mt. Peak, 14 vii 1936, Avinoff & Shoumatoff [examined], genitalia slide #12275 [examined], KAA_DNA_0035 (CMNH).

###### Redescription.

**Male. (n = 1). *Head*.** Scales on vertex missing, scales on frons and frons red-orange, intermixed with dark brown scales. Labial palpus with lateral surface red-orange, becoming predominantly dark brown towards apex, slightly iridescent when viewed at an angle under light; medial surface pale yellow. Scape dark brown, brick red at apex. Dorsal scales of flagellum with segments of basal third pale yellow, alternating rows of pale yellow and warm brown beyond. Sensillae 1 × width of flagellomere, nearly porrect, but slightly hooked apically. ***Thorax*.** Dorsum of pro- and mesothorax light brown with a few brick red scales; tegulae concolorous, but with more brick red scales. Forelegs missing; midleg with lateral surface straw yellow, tibia silvery brown; hindlegs straw yellow to pale yellow. Dorsal surface of forewing (Fig. 11F) with banding faint; antemedian and postmedian interfasciae warm brown with faint darker reticulations, basal fascia, median fascia, and postmedian fascia brown, median fascia most distinct, darker than interfasciae, scattered pinkish-orange scales visible under magnification; fringe pale red-orange, chalky gray at apex; apex slightly produced; FWL 8.5 mm. Dorsal surface of hindwing uniformly pale brown, no strigulae apparent; fringe concolorous, slightly darker at apex. Ventral surface of both wings pale brown, a few red-orange scales present along forewing costa. ***Abdomen*.** Vestiture unknown. Genitalia (Fig. 18D) with uncus divergently bifurcate, branches thin, pointed at apices; apicoventral setae projecting from apices; socii not observed; arms of gnathos moderate, smooth; terminal plate smoothly rounded with medial ridge; tegumen robust, unmodified; transtilla with large pointed lateral processes, complete; valvae triangular, elongate, rounded on ventral edge, patch of deciduous setae at base not observed; sacculus to 0.9 ×; juxta with broad V-shaped notch, short setae not observed on lateral lobes; phallus pistol-shaped, sharply elongate and acute at apex, caulis pronounced; three cornuti observed: thin, straight, approximately 0.25 × length of phallus.

###### Description.

**Female.** Female unknown.

###### Distribution.

*Claduncaria rufochlaena* is known from a single male collected on Blue Mountain Peak, the highest point of Jamaica with a peak elevation of 2256 m (Fig. 25A).

###### Biology.

Nothing is known of the biology of *Cla. rufochlaena*. The only known specimen was collected in July.

###### Remarks.

See remarks under *Cla. chalarostium* concerning possibly conspecificity with that species. Our Maximum Likelihood analysis (Fig. 4) suggest that *Cla. rufochlaena* may belong to the *ochrochlaena* group. Though it does possess a divergently bifid uncus, other characters such as the rounded terminal plate of the gnathos and shape of the valva support its inclusion in the *mesosignaria* group. In the absence of more robust molecular sampling, we choose to include *Cla. rufochlaena* in the *mesosignaria* group.

##### Claduncaria taino sp. nov.

Taxon classificationAnimaliaLepidopteraTortricidae

3B60EE84-AA2A-5659-A02D-D2A47E247746

http://zoobank.org/7617D4C0-6E97-4B50-AFF1-A25F0EE1B940

Figs 11G , 18G , 25A

###### Diagnosis.

Males of *Claduncaria taino* (Fig. 11G) are most likely to be confused with those of *Cla. maestrana* (Fig. 10B) from Cuba. The male genitalia of *Cla. taino* (Fig. 18G) differs from those of *Cla. maestrana* (Fig. 18A) in lacking a strongly divergently bifurcate uncus and possessing a terminal plate of the gnathos without a vertical bifurcation. Females are unknown.

###### Type material.

***Holotype*** ♂: **Dominican Republic: La Vega**: Cordillera Central, Loma Casabito, 15.8 km NW Bonao, 19°02'12"N, 70°31'08"W, 1455 m, 28 v 2003, evergreen cloud forest, east slope, J. Rawlins, C. Young, R. Davidson, C. Nunez, P. Acevedo. HOLOTYPE *Claduncaria taino* Austin & Dombroskie [typed red label] (CMNH). ***Paratypes*** (2♂♂): **Dominican Republic: La Vega**: 2♂♂, Loma del Casabito, 19°03'N, 70°31'W, 1390 m, 3 xi 2002, wet cloud forest, W.A. Zanol, C.W. Young, C. Staresinic, J. Rawlins (CUIC, CMNH). KAA diss. #0119, KAA_DNA_0041 (CMNH). All paratypes affixed with the following typed blue label: PARATYPE ♂ *Claduncaria taino* Austin & Dombroskie, 2020.

###### Description.

**Male (n = 3). *Head*.** Scales on vertex and frons white, a few brick red and brown scales present near base of antenna. Labial palpus with scales on lateral surface of first segment red-orange, second segment with lateral surface red-orange on basal half, white on apical half, third segment white; medial surface of palpus white. Scape dark brown with a few brick red and white scales. Dorsal scales of flagellum with alternating rows of white and brown. Sensillae 0.5–0.75 × width of flagellomere, nearly porrect. ***Thorax*.** Dorsum of pro- and mesothorax white to pale brown; tegulae concolorous. Foreleg and midleg with lateral surface red-orange and dark brown scaling, tarsi dark brown; hindlegs white. Medial surface of legs white. Dorsal surface of forewing (Fig. 11G) silvery-white with dark brown median fascia and subapical blotch, brick red scales present along inner margin of median fascia under magnification, fringe chalky gray-brown, becoming paler at tornus; FWL 6.0–7.0 mm (mean = 6.5; n = 3). Dorsal surface of hindwing white with faint brown strigulae and shading near apex; fringe concolorous, including faint brown scales near apex. Ventral surface of forewing warm brown, light red-orange along costa. Ventral surface of hindwing as on dorsal surface but lacking brown shading, making brown strigulae appear more prominent. ***Abdomen*.** Vestiture white to pale brown, terminal segment straw yellow. Genitalia (Fig. 18G) with uncus Y-shaped, medial notch deeper than in similar members of *mesosignaria* group; apicoventral setae projecting from apices; socii present as small nubs with projecting setae (not observed in *Cla. rufochlaena*); arms of gnathos moderate, minutely roughened on lateral surface; terminal plate long, smoothly rounded at apex with medial ridge; tegumen robust, unmodified; transtilla broad, unadorned; valvae triangular, flat on dorsal edge, rounded on ventral edge, lacking patch of deciduous setae at base; sacculus to 0.8 ×; juxta with deep V-shaped notch, short setae not observed on lateral lobes; phallus pistol-shaped, sharply acute at apex, caulis small; two cornuti observed in one specimen examined: thin, straight, approximately 0.33 × length of phallus.

**Female.** Female unknown.

###### Etymology.

The specific epithet honors the Taíno people, the principle inhabitants of Hispaniola prior to European colonization.

###### Distribution.

*Claduncaria taino* is known from the vicinity of Loma del Casabito in the Cordillera Central of the Dominican Republic (Fig. 25A). Collection localities range from 1390 to 1455 m elevation.

###### Biology.

Nothing is known of the biology of *Cla. taino*. Capture dates of examined specimens are from May and November, suggesting multiple generations per year.

###### Remarks.

See remarks under *Cla. praedictana*. We predict that the yet-to-be discovered females of *Cla. taino* will possess apically swollen papillae anales without a ventroposterior groove, similar to other members of the *mesosignaria* group.

##### Clepsis

Taxon classificationAnimaliaLepidopteraTortricidae

Guenée, 1845

78FC30B0-367D-51EB-9B35-CC25E6A35527

###### Type species.

*Tortrix rusticana* Hübner [1796–1799] *sensu* Treitschke, 1830 [= *Tortrix senecionana* Hübner, [1818–1819]

*Clepsis* Guenée, 1845: 149

*Clepsodes* Diakonoff, 1957 [subgenus of *Clepsis*]: 240

*Mochlopyga* Diakonoff, 1964: 44

*Pseudamelia* Obraztsov, 1954 [subgenus of *Clepsis*]: 196

*Siclobola* Diakonoff, 1948: 25

*Smicrotes* Clemens, 1860: 355

###### Remarks.

The following description is specific to Caribbean *Clepsis*. The Caribbean species of *Clepsis* are not conspecific with *Tortrix senecionana* Hübner, [1818–1819], the type species of *Clepsis*. *Smicrotes* Walker, currently a synonym of *Clepsis*, may need to be resurrected to accommodate many species currently placed in *Clepsis*, including all the Caribbean species mentioned below. As it currently stands, *Clepsis* is paraphyletic and in need of careful taxonomic revision.

###### Redescription.

**Figure 12.**
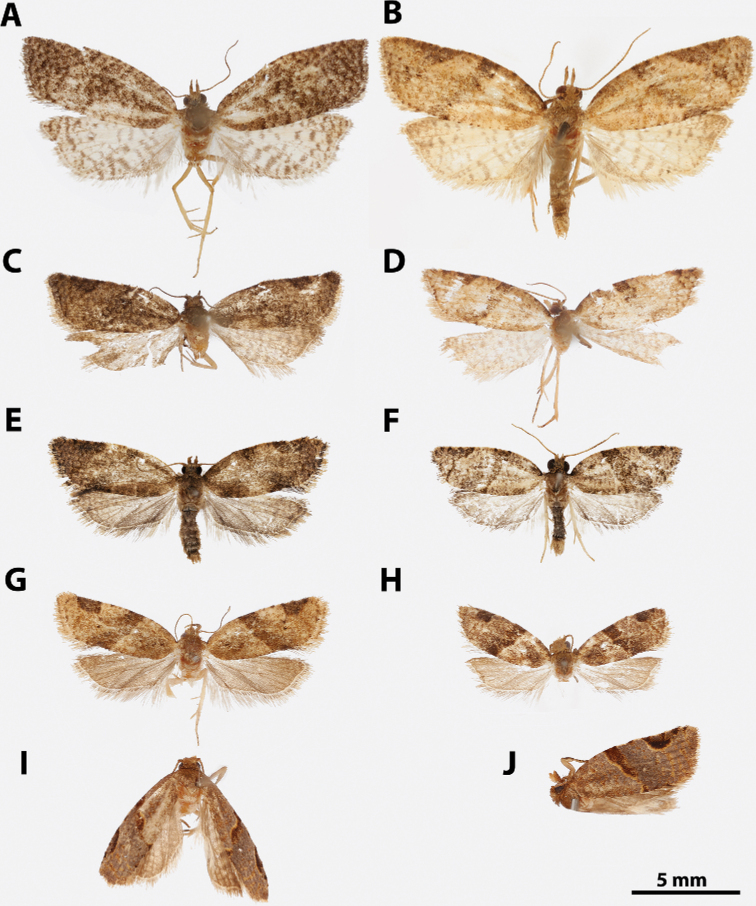
*Clepsis* adults. **A***Cle. deroni* sp. nov. holotype ♀, Dominican Republic (CMNH) **B***Cle. deroni* sp. nov. paratype ♂, Dominican Republic (CUIC) **C***Cle. jamesstewarti* sp. nov. holotype ♀, Dominican Republic (CMNH) **D***Cle. jamesstewarti* sp. nov. paratype ♂, Dominican Republic (CMNH) **E***Cle. davisi* sp. nov. holotype ♀, Guadeloupe (CUIC) **F***Cle. davisi* sp. nov. paratype ♂, Guadeloupe (CUIC) **G***Cle. peritana* ♀, Cuba (USNM) **H***Cle. peritana* ♂, Cuba (CUIC) **I***Cle. peroniae* sp. nov. holotype ♀, Dominican Republic, dorsal (USNM) **J** same as previous, lateral, rotated for ease of comparison.

Labial palpus 1.5–2.0 × width of compound eye; second segment expanded apically. Ocellus minute, separated from compound eye by approximately 0.5–1.0 × width of ocellus. Chaetosemata 0.25–0.75 × length of scales on vertex. Dorsal scaling on metathorax absent, with a small patch of setae present instead, usually concolorous with cubital pecten. Costal fold absent; costa with basal third gently curved, straight beyond, never with concavity along distal third like in some species of *Argyrotaenia*. Forewing pattern (Fig. 12) generally with ground color straw yellow to brown, never with red scaling like in many species of *Argyrotaenia* and *Claduncaria*. Generally smaller (FWL 4.0–6.5 mm) than most species of *Argyrotaenia* and *Claduncaria*, although *Cle. deroni* (Fig. 12A, B) is unusually large for *Clepsis* (FWL 7.0–9.5 mm). Some species sexually dimorphic in forewing coloration. Male genitalia (Fig. 20A–C, Austin et al. 2019: fig. 3e) with uncus with weakly-developed bulb; gnathos with arms evenly curved, acutely united apically; socii obsolete; tegumen moderate; labides separate, densely spined; valvae triangular, weakly-sclerotized; juxta near-hexagonal, with or without dorsal notch; phallus variable. Female genitalia (Fig. 21A–D; Austin et al. 2019: fig. 4d) wildly variable: papillae anales usually triangular, but occasionally broadly rectangular (*Cle. deroni*, Fig. 21A); ductus bursae usually tightly coiled with cestum present, but occasionally only loosely coiled and cestum absent (*Cle. peroniae*, Fig. 21C); signum usually present but occasionally absent (*Cle. peritana*, see Austin et al. 2019: fig. 4d); capitulum present or absent.

### Key to species for females of Caribbean *Clepsis*

**Table:** 

1	Signum absent (Austin et al. 2019: fig. 4d); Cuba, The Bahamas	***Cle. peritana***
–	Signum present (Fig. 21A–D)	**2**
2	Cestum absent (Fig. 21C); Hispaniola	***Cle. peroniae* sp. nov.**
–	Cestum present (Fig. 21A, B, D)	**3**
3	Capitulum absent (Fig. 21A, C); FWL > 7.0 mm; Hispaniola	***Cle. deroni* sp. nov.**
–	Capitulum present (Fig. 21B, D); FWL < 7.0 mm	**4**
4	Lateral edges of sterigma with short anterior extensions (Fig. 21D); Guadeloupe, Dominica	.***Cle. davisi* sp. nov.**
–	Lateral edges of sterigma without short anterior extensions (Fig. 21B); Hispaniola	***Cle. jamesstewarti* sp. nov.**

### Key to species for males of Caribbean *Clepsis*^3^

**Table:** 

1	Phallus distinctly bent ventrally (Fig. 20A); FWL > 7.0 mm (Fig. 12B); Hispaniola	***Cle. deroni* sp. nov.**
–	Phallus not distinctly bent ventrally; FWL < 7.0 mm	**2**
2	Labides large, globose, densely spined, nearly joined mesally; neck of uncus broad (Fig. 20C); Guadeloupe, Dominica	***Cle. davisi* sp. nov.**
–	Labides more sparsely spined, not appearing inflated or globose, broadly separated mesally; neck of uncus narrow	**3**
3	Phallus elongate with distinctly acute apex (Fig. 20B); Hispaniola	***Cle. jamesstewarti* sp. nov.**
–	Phallus shorter with less distinctly acute apex (Austin et al. 2019: fig 3e); Cuba, The Bahamas	***Cle. peritana***

#### Clepsis deroni sp. nov.

Taxon classificationAnimaliaLepidopteraTortricidae

85D9483B-53C5-5ED9-A58C-DFE21B108EF4

http://zoobank.org/FB9335AF-FD0C-486E-8672-4B245613E3C6

Figs 12A, B , 20A , 21A , 26

##### Diagnosis.

*Clepsis deroni* can be separated from all other Caribbean *Clepsis* by its large size (FWL 7.0–9.5 mm; Fig. 12A, B) and female genitalia with a thin, straight signum without a capitulum (Fig. 21A).

##### Type material.

***Holotype*** ♀: **Dominican Republic: Peravia [San José de Ocoa**]: 3 km SW La Nuez, upper Rio Las Cuevas, 1880 m, cloud forest on river, 18°39'N, 70°36'W, 5–6 x 1991, J. Rawlins, R. Davidson, C. Young, S. Thompson. KAA diss. #0058. HOLOTYPE *Clepsis deroni* Austin & Dombroskie [typed red label] (CMNH). ***Paratypes*** (8♂♂, 7♀♀): **Dominican Republic: Peravia [San José de Ocoa**]: 6♂♂, 4♀♀, same data as holotype (2♂♂, 1♀ CUIC; remainder CMNH, including 1♀ KAA_DNA_0049). KAA diss. #0057(♂) (CMNH). 1♀, same as holotype except 2 ix 1995, J. Rawlins, G. Onore, R. Davidson; KAA diss. #0067; KAA_DNA_0051 (CMNH). **La Vega**: 2♂♂, 2♀♀, Reserva Cientifica Valle Nuevo, Sector La Nevera, 3 km WNW La Nuez, 2200 m, 18°42'N, 70°36'W, 7 x 1991, C. Young, S. Thompson, R. Davidson, J. Rawlins, mesic pine woodland (1♀ CUIC; remainder CMNH, including 1♀ KAA_DNA_0052); KAA diss. #0078(♂), KAA_DNA_0050 (CMNH). All paratypes affixed with the following typed blue label: PARATYPE ♂/♀ *Clepsis deroni* Austin & Dombroskie, 2020.

##### Description.

**Male (n = 8). *Head*.** Scales on vertex and frons straw yellow to pale brown. Scaling on lateral surface of labial palpus with first segment straw yellow, second and third segment pale brown, becoming slightly darker apically; medial surface of labial palpus pale yellow. Scape concolorous with vertex, a few dark brown scales present dorsally. Dorsal scales of flagellum with alternating rows of brown basal scales and straw yellow apical scales. Sensillae approximately 1 × width of flagellomere, nearly porrect. ***Thorax*.** Dorsum of pro- and meso-thorax with scaling sometimes concolorous with vertex, but usually brown; tegulae concolorous. Foreleg and midleg with lateral surface dark brown; hindlegs pale brown to straw yellow. Medial surface of legs straw yellow. Dorsal surface of forewing (Fig. 12B) with ground color dirty straw yellow, heavily mottled with dark brown, giving forewing a strongly mottled look; basal fascia obsolete; median fascia and subapical blotch dark brown; fringe predominantly straw yellow, a few long, scattered pale brown scales present; FWL 7.0–8.5 mm (mean = 7.8; n = 8). Dorsal surface of hindwing white to pale brown, heavily marked with dense, dark brown strigulae; fringe similar to forewing fringe. Ventral surface of forewing dark brown, straw yellow markings present along costa. Ventral surface of hindwing concolorous with dorsal surface, but strigulae more distinct. ***Abdomen*.** Vestiture warm brown. Genitalia (Fig. 20A) with neck of uncus, moderate, parallel-sided; bulb wide, subquadrate, densely covered with apicoventral setae; socii obsolete; arms of gnathos narrow, evenly curved throughout entire length, joined acutely at apex; tegumen moderate, unadorned; labides spinulate; valvae triangular, weakly-sclerotized, produced at apex; sacculus well-developed, to 0.5 ×; juxta hexagonal with moderate dorsal notch. Phallus (Fig. 20A) irregularly shaped, angled at approximately 140°; caulis minute; one spindle-shaped cornutus observed in one specimen.

**Female (n = 8)**. ***Head*.** As in male, but scaling almost entirely dark brown. Sensillae minute, no more than 0.5 × width of flagellomere. ***Thorax*.** As in male but dorsum of pro- and meso-thorax with scaling entirely dark brown. Dorsal surface of forewing (Fig. 12A) entirely dark brown and heavily mottled, as to almost entirely obscure fasciae, which are distinct in male; forewing slightly narrower than in male; fringe entirely dark brown; FWL 7.0–9.5 mm (mean = 8.2; n = 8). Dorsal surface of hindwing as in male but without any yellow scaling, white instead; strigulae more contrasting; hindwing fringe concolorous with forewing fringe but with long off-white scales present along entire margin. Ventral surface of both wings as in male. ***Abdomen*.** Vestiture dark brown. Genitalia (Fig. 21A) with papillae anales broad, rectangular; apophyses posteriores approximately 0.5 × length of sternum VII, straight; apophyses anteriores approximately 0.67 × length of sternum VII, straight; sterigma moderate, ventral portion well-sclerotized; antrum narrow, lightly sclerotized; colliculum present as ring-like structure, but sclerotization absent ventrally; ductus bursae long, coiled; cestum present; ductus seminalis arising at approximately 0.1 × length of ductus bursae; corpus bursae nearly perfectly spherical; signum short to moderate, thin; capitulum absent.

##### Etymology.

This species is named in honor of KAA’s father, Deron Austin, for his unwavering support and love.

##### Distribution.

*Clepsis deroni* is known from two close localities in the southern portion of the Cordillera Central range on the border of San José de Ocoa and La Vega provinces (Fig. 26). Collection localities range from 1880 to 2200 m elevation.

##### Ecology.

Nothing is known of the biology of *Cle. deroni*. All but one of the type series were collected in October; the other was collected in September.

##### Remarks.

COI sequences for 4 barcoded specimens of *Cle. deroni* were identical.

#### Clepsis jamesstewarti sp. nov.

Taxon classificationAnimaliaLepidopteraTortricidae

9D8417BE-E1D4-5F19-94D7-A258ACB5F7A7

http://zoobank.org/40C8E4D0-F61C-4E8C-81E3-182EB5AAED4D

Figs 12C, D , 20B , 21B , 26

##### Diagnosis.

*Cle. jamesstewarti* (Fig. 12, D) is similar to *Cle. deroni* (Fig. 12A, B), but smaller and with narrower forewings. Forewing length alone should be sufficient to separate the two externally (5.5–6.5 mm in *Cle. jamesstewarti*, 7.0–9.5 mm in *Cle. deroni*), but the genitalia are distinct as well (see diagnosis under *Cle. deroni*). Both male (Fig. 20B) and female (Fig. 21B) genitalia are similar to those of *Cle. davisi* (see diagnosis under that species). Male genitalia are extremely similar to *Cle. peritana* but can be separated by the shape of the phallus: narrow and elongate with a distinctly acute apex in *Cle. jamesstewarti* (Fig. 20B), noticeably broader and with the apex less acute in *Cle. peritana* (see Austin et al. 2019: fig. 3e).

##### Type material.

***Holotype*** ♀: **Dominican Republic: Pedernales**: 5 km NE Los Arroyos, 1680 m, 18°15'N, 71°45'W, 30 ix 1991, R. Davidson, C. Young, S. Thompson, J. Rawlins; KAA diss. #0149; KAA_DNA_0072. HOLOTYPE *Clepsis jamesstewarti* Austin & Dombroskie [typed red label] (CMNH). ***Paratypes*** (8♂♂, 6♀♀): **Dominican Republic: Independencia**: 4♂♂, 3♀♀, Sierra de Bahoruco, north slope, 13.5 km SE Puerto Escondido, 1789 m, 18°12'18"N, 71°31'08"W, 24–25 xi 2004, ecotonal *Pinus* grassland, J.E. Rawlins, C. Young, C. Nunez, V. Verdecia, W.A. Zanol (1♂ CUIC, remainder CMNH). KAA diss. #0142(♂), KAA_DNA_0071 (CMNH); KAA diss. #0188(♂, CMNH); KAA diss. #0189(♂, CMNH); KAA diss. #0190(♀), KAA_DNA_0079 (CMNH). 2♂♂, 3♀♀, Sierra de Bahoruco, north slope, 2116 m, broadleaf forest with pine, 18°41'31"N, 71°35'35"W [18°17'30"N, 71°43'08"W], 8 xi 2002, W.A. Zanol, C.W. Young, C. Staresinic, J. Rawlins (1♂ CUIC, remainder CMNH); KAA diss. #0061(♂, CUIC); KAA diss. #0063(♀, CMNH); KAA diss. #0141(♂), KAA_DNA_0069 (CMNH); KAA diss. #0148(♀, CMNH); KAA diss. #0187(♀), KAA_DNA_0076 (CMNH). 1♂, Sierra de Bahoruco, Loma del Toro, 18°17'16"N, 71°42'46"W, 2310 m, 7–8 xi 2002, meadow in pine woods, W.A. Zanol, C.W. Young, C. Staresinic, J. Rawlins; KAA diss. #0065; KAA_DNA_0078 (CUIC). **Pedernales**: 1♂, same data as holotype except 18 x 1991 (CMNH). 1♂ same data as holotype except 20 x 1991, KAA diss. #0143 (CMNH). All paratypes affixed with the following typed blue label: PARATYPE ♂/♀ *Clepsis jamesstewarti* Austin & Dombroskie, 2020.

##### Additional material examined.

(16♂♂, 12♀♀) **Dominican Republic: Dajabon**: 1♀, 13 km S. Loma de Cabrera, ca. 400 m, 20–22 v 1973, Don & Mignon Davis; KAA diss. #0139; USNMENT01480226 (USNM). **Independencia**: 1♂, Sierra de Bahoruco, north slope, 13.5 km SE Puerto Escondido, 1789 m, 18°12'18"N, 71°31'08"W, 24–25 xi 2004, ecotonal *Pinus* grassland, J.E. Rawlins, C. Young, C. Nunez, V. Verdecia, W.A. Zanol; KAA diss. #0068; KAA_DNA_0068 (CMNH). 7♂♂, 3♀♀, Sierra de Neiba, just south of crest, 5 km WNW Angel Feliz, 18°41'N, 71°47'W, 1780 m, 13–15 x 1991, cloud forest, J. Rawlins, R. Davidson, C. Young, S. Thompson (1♂, 1♀ CUIC, remainder CMNH). KAA diss. #0059(♂, CMNH); KAA diss. #0062(♀), KAA_DNA_0075; KAA diss. #0136(♀, CMNH); #0145(♂), KAA_DNA_0070 (CMNH); KAA diss. #0191(♂, CMNH); KAA diss. #0192(♂, CMNH), KAA diss. #0194(♀, CMNH). 1♀, Sierra de Neiba, south slope near summit, 4.0 km N Angel Feliz, broadleaf cloud forest without pine, 1825 m, 18°40'21"N, 71°46'05"W, 1 v 2006, J. Hyland, C. Young, R. Davidson, D. Koenig, J. Fetzner, J. Rawlins. KAA diss. #0137, KAA_DNA_0077 (CMNH). 3♂♂, same as previous, but 1–2 iv 2004, J. Rawlins, C. Young, R. Davidson. KAA diss. #0144; KAA diss. #0193, KAA_DNA_0080 (CMNH). **La Vega**: 1♂, 2.5 km SW Pinar Bonito, 1430 m, 18°51'N, 70°43'W, riparian vegetation near stream in pine woodland, 26 xi 1992, J. Rawlins, R. Davidson, M. Klingler, S. Thompson; KAA diss. #0048; KAA_DNA_0073 (CMNH). 6♀♀, Convento, 12 km S of Constanza, 6–13 vi 1969, Flint, Gomez (1♀ CUIC, remainder USNM). KAA diss. #0069, USNMENT01480227 (USNM); KAA diss. #0138 (USNM); KAA diss. #0195 (USNM). **Pedernales**: 2♂♂, 1♀, 1 km S Los Arroyos, 1125 m, 18°14'N, 71°45'W, second growth forest, 18 x 1991, R. Davidson, C. Young, S. Thompson, J. Rawlins. KAA diss. #0066(♂), KAA_DNA_0067; KAA diss. #0186(♀) (CMNH). 1♂, same data as holotype (CMNH). **Haiti: Ouest**: 1♂, Kenscoff, 1310 m, 30 iv 1937, Roys. Clepsis ? developa Meyrick. Razowski. diss. #12282 [only genitalia slide examined] (CMNH).

##### Description.

**Male (n = 8). *Head*.** Scales on vertex and frons straw yellow to golden brown. Scaling on lateral surface of labial palpus straw yellow with scattered dark brown scales, second segment expanded apically. Scape concolorous with vertex. Dorsal scales of flagellum with alternating rows of warm brown basal scales and straw yellow apical scales. Sensillae approximately 0.75 × width of flagellomere, nearly porrect. ***Thorax*.** Dorsum of pro- and meso-thorax with scaling concolorous with vertex; tegulae similarly colored. Foreleg and midleg with lateral surface with brown scaling; hindlegs pale yellow to white, tarsi brown. Medial surface of legs straw yellow to white. Forewing relatively narrow; dorsal surface of forewing (Fig. 12D) with ground color straw yellow, mottled with dark brown in interfasciae; basal fascia obsolete; median fascia entire to inner margin, dark brown; subapical blotch dark brown, variously developed; fringe with short scales pale brown, longer scales straw yellow; FWL 5.5–6.5 mm (mean = 6.0; n = 8). Dorsal surface of hindwing white to pale brown, heavily marked with dense, dark brown strigulae; hindwing fringe similar to forewing fringe. Ventral surface of forewing dark brown, with straw yellow markings along costa. Ventral surface of hindwing concolorous with dorsal surface. ***Abdomen*.** Vestiture warm brown. Genitalia (Fig. 20B) with neck of uncus narrow, widening slightly to form rounded bulb; socii obsolete; arms of gnathos narrow, evenly curved throughout entire length, joined acutely at apex; tegumen moderate, unadorned; labides small to moderate, spinulate, evenly rounded; valvae triangular, weakly-sclerotized, produced slightly at apex; sacculus moderate, to 0.6 ×; juxta hexagonal, medial notch variable, but never V-shaped. Phallus (Fig. 20B) swollen dorsomedially, not distinctly angled ventrally, apex very acute; caulis small to minute. Cornuti thin, slightly undulate.

**Female (n = 7). *Head*.** As in male, but scaling almost entirely dark brown. Sensillae minute, no more than 0.25 × width of flagellomere. ***Thorax*.** As in male but dorsum of pro- and meso-thorax with scaling entirely dark brown, tegulae dark brown. Dorsal surface of forewing (Fig. 12C) with ground color brown, heavily mottled; median fascia dark brown, scarcely distinct; subapical botch dark brown, slightly darker, variously developed; fringe with short scales dark brown, longer scales straw yellow; FWL 6.0–6.5 mm (mean = 6.1; n = 7). Dorsal surface of hindwing as in male; hindwing fringe concolorous with forewing fringe. Ventral surface of both wings as in male, but darker. ***Abdomen*.** Vestiture dark brown. Genitalia (Fig. 21B) with papillae anales triangular; apophyses posteriores approximately 0.5 × length of sternum VII, straight; apophyses anteriores approximately 0.67 × length of sternum VII, slightly outcurved; sterigma bowl-shaped, thinly sclerotized, small medial-facing lateral sclerotizations on dorsal portion; antrum constricted; colliculum present as small, weakly-sclerotized ring-like structure; ductus bursae long, tightly coiled; cestum present; ductus seminalis arising near base of ductus bursae; corpus bursae spherical; signum short to moderate, straight; capitulum present as small cylindrical projection.

##### Etymology.

This species is named in loving memory of James Peter Stewart (1995–2019), Cornell University entomology graduate student and dear friend of KAA.

##### Distribution.

This is the most commonly collected species of *Clepsis* on Hispaniola, with specimens ranging from 400 to 2310 m elevation. The type locality is restricted to Sierra de Bahoruco, but additional specimens were collected in the Sierra de Neiba and Cordillera Central in the Dominican Republic and Chaîne de la Selle in Haiti (Fig. 26).

##### Ecology.

Nothing is known of the biology of *Cle. jamesstewarti*. The type series was collected from September to November. Non-type specimens range in capture date from April to November.

##### Remarks.

A genitalia slide of a male of this species was found in CMNH, but the adult specimen, from Kenscoff, Haiti, could not be located. Razowski had labeled the slide as “Clepsis ? developa Meyr.,” however, we could find no published record of this Meyrick name, and thus we treat it as unavailable.

There is a discrepancy in the label data of five paratypes from Independencia. The label data reads “Sierra de Bahoruco,” but the coordinates are for the Sierra de Neiba. After comparing coordinates from specimens collected the previous night and discussing the situation with Dr. John Rawlins (CMNH), we interpret the coordinates to be incorrect. Dr. Rawlins kindly supplied us with the correct coordinates.

We examined a large number of specimens from other localities on Hispaniola and were unable to find consistent genitalic differences among them and the type series of *Cle. jamesstewarti*. However, COI sequence divergence between populations in the Sierra de Bahoruco and the Sierra de Neiba/Cordillera Central was high (3.7–5.3%). In light of this, we restrict the type series to specimens from Sierra de Bahoruco (excluding an unusual male and female). Maximum COI sequence divergence for barcoded type specimens was 0.9%. We refrain from describing the other populations as a different species due to the absence of observed morphological differences.

#### Clepsis davisi sp. nov.

Taxon classificationAnimaliaLepidopteraTortricidae

112E85EE-1C06-5BCB-BEAD-70E233148372

http://zoobank.org/D9EFFFFD-86D8-4797-A925-3B7293F315C6

Figs 12E, F , 20C , 21D , 27

##### Diagnosis.

Within the Caribbean, *Clepsis davisi* (Fig. 12E, F) is most similar to *Cle. jamesstewarti* (Fig. 12C, D), but females can be separated by the presence of a small anterior projection on the lateral edges of the sterigma (Fig. 21D), which are absent in *Cle. jamesstewarti* (Fig. 21B). Males can be separated by the larger, more densely spinose labides (Fig. 20C) than those in *Cle. jamesstewarti* (Fig. 20B).

##### Type material.

***Holotype*** ♀: **Guadeloupe: St.-Claude**: Sentier du Matouba, 16.048, -61.691, 11 vi 2019, K.A. Austin, J.J. Dombroskie, UV LED light, 723 m, JD41526. KAA diss. #0183. HOLOTYPE *Clepsis davisi* Austin & Dombroskie [typed red label] (CUIC). **Paratypes** (26♂♂, 17♀♀): **Dominica: St. David**: 1♂, 1♀, 2.2 mi E. of Pont Cassé, 11 v 1964, O.S. Flint. Jr (USNM). 1♀, same as previous, but 2 v 1964 (CUIC). 1♀, same as previous, but 19 v 1964 (USNM). 1♀, same as previous, but 21 v 1964 (USNM). 1♂, Fond Figures, 1 v 1965, D.R. Davis (CUIC). **St. George**: 1♂, Freshwater, 5–8 xi 1966, A.B. Gurney (USNM). 1♀, Sylvania, Mt. Trois Pitons, 1800 ft., 9 ii 1964, D.F. Bray. KAA diss. #0180 (USNM). 1♂, Trafalgar, 10 vi 1965, D.R. Davis; USNMENT01480258 (USNM). **St. Joseph**: 1♂, 2.5 mi N Pont Cassé, 8 iv 1965, D.R. Davis (USNM). 2♂♂, 1♀, Clarke Hall, 20–27 iii 1965, J.F.G., T.M. Clarke (1♂ CUIC, remainder USNM). **St. Paul**: 1♂, 0.5 mi S Pont Cassé, 5 iv 1965, D.R. Davis (CUIC). 2♂♂, 1 mi E Pont Cassé, 29 i 1965, J.F.G. Clarke, Thelma M. Clarke. KAA diss. #0182 (USNM). 1♂, 1♀, 1 mi N Pont Cassé, 15 iv 1965, D.R. Davis (USNM). 1♀, 1.3 mi E of Pont Cassé, 29 iv 1964, O.S. Flint, Jr. KAA diss. #0179; USNMENT01480274 (USNM). 2♀♀, same as previous, but 10 v 1964 (CMNH, USNM). 3♂♂, 3♀♀, same as previous, but 11 vi 1964 (1♂, CMNH, 1♀ CUIC, remainder USNM). 1♂, 1.5 mi NW Pont Cassé, 3 iv 1965, D.R. Davis (USNM). 1♂, 1 mi N Pont Cassé, 15 iv 1965, D.R. Davis (USNM). 1♂, 2 mi NW Pont Cassé, 16 iv 1965, D.R. Davis (CUIC). 1♀, same as previous, but 25 v 1965 (CUIC). 3♂♂, Pont Cassé, 6 iv 1965, D.R. Davis (USNM). 1♀, same as previous, but 19 v 1965 (CUIC). 3♂♂, Springfield, 1 vi 1965, D.R. Davis. KAA diss. #0181 (USNM). **Guadeloupe: St.-Claude**: 2♂♂, 1♀, Rue des Pimentiers, 16.043, -61.688, 12 vi 2019, K.A. Austin, J.J. Dombroskie, UV LED light, 827 m (CUIC). 1♀, same as previous, but 10–11 vi 2019 (CUIC). 1♂, same as holotype, except JD4157. KAA diss. #0184 (CUIC). All paratypes affixed with the following typed blue label: PARATYPE *Clepsis davisi* ♂/♀ Austin & Dombroskie, 2020.

##### Description.

**Male (n = 26). *Head*.** Scales on vertex and frons straw yellow to warm brown. Scaling on lateral surface of labial palpus with first and second segments golden brown, third segment straw yellow; medial surface of labial palpus straw yellow. Scape bicolored: golden brown anteriorly and straw yellow posteriorly. Dorsal scales of flagellum with alternating rows of dark brown and golden yellow scales, becoming predominantly darker apically. Sensillae porrect, 0.75–1.0 × width of flagellomere. ***Thorax*.** Dorsum of pro- and meso-thorax with scaling concolorous with vertex (one aberrant specimen dark brown, nearly black); tegulae similarly colored. Foreleg with lateral surface dark brown; midleg similarly colored, but with golden yellow scales occasionally present; hindlegs pale brown to straw yellow. Medial surface of legs straw yellow. Dorsal surface of forewing (Fig. 12F) with ground color straw yellow; basal fascia usually obsolete, median fascia and subapical blotch warm brown to dark brown (nearly black in one aberrant specimen), subapical blotch occasionally continuing to near tornus as thin line; heavily mottled throughout; interfasciae occasionally suffused with gray. Fringe with short scales dark gray, nearly black; longer scales straw yellow; FWL 4.0–6.0 mm (mean = 5.2; n = 26). Dorsal surface of hindwing gray, faint strigulae present at apex; fringe predominantly dark gray, a few long off-white scales present along extreme posterior margin and apex. Ventral surface of forewing warm brown to gray, forewing markings visible along costa. Ventral surface of hindwing similar to dorsal surface but slightly paler with strigulae more distinct. ***Abdomen*.** Vestiture silver-gray, concolorous with dorsal surface of hindwing, terminal segment straw yellow. Genitalia (Fig. 20C) with uncus moderate, widening apically to rounded bulb; socii present as minute setose nubs; arms of gnathos moderate, evenly curved throughout entire length, joined acutely at apex; tegumen moderate, unadorned; labides large, globose, densely spined (large enough that they almost form a complete transtilla); valvae triangular, weakly-sclerotized; sacculus well-developed, to 0.5 × valva length; juxta hexagonal with moderate dorsal notch. Phallus (Fig. 20C) irregularly shaped, angled at 180° on ventral margin, with semicircular swelling mesally on dorsal margin; caulis minute to obsolete; two deciduous spindle-shaped cornuti observed in one specimen.

**Female (n = 18). *Head*.** Vertex, frons, and labial palpus as in male, but scaling darker throughout, never straw yellow. Flagellomeres with more extensive straw yellow scaling. Sensillae minute, no more than 0.5 × width of flagellomere, porrect. ***Thorax*.** Scaling on dorsum of pro-, meso-thorax, and tegulae concolorous with vertex. Legs as in male. Dorsal surface of forewing (Fig. 12E) narrower than in male, dark brown, sometimes so dark as to obscure median fascia and subapical blotch. When not obscured, median fascia and subapical blotch darker brown, nearly black in some specimens; median fascia bordered on inner margin by thin line of straw yellow scales (line present in males, but difficult to see because of lack of contrast); subterminal blotch occasionally continuing to near tornus as a thin line; interfasciae often strongly suffused with purple-gray scales; mottled throughout, but not as noticeably as in males; fringe as in male but short scales darker, black; FWL 6.0–7.0 mm (mean = 6.3; n = 18). Dorsal surface of hindwing gray to pale brown, strigulae as in male; hindwing fringe similar to forewing fringe but with long scales gray-brown along posterior margin. Ventral surfaces of both wings as in male. ***Abdomen*.** Vestiture entirely concolorous with dorsal surface of hindwing. Genitalia (Fig. 21D) with papillae anales triangular; apophyses posteriores 0.5 × length of sternum VII, straight; apophyses anteriores approximately 0.75 × length of sternum VII, straight; sterigma well-sclerotized, with small lateral convexity and short, anterior, unsclerotized extension; antrum narrow, lightly sclerotized; colliculum present as a ring-like structure, but sclerotization absent ventrally, tightly constricted anteriorly; ductus bursae long, coiled; cestum present, beginning at approximately 0.2 × length of ductus bursae; ductus seminalis arising at approximately 0.05 × length of ductus bursae; corpus bursae spherical; signum short, capitulum present; basal plate obsolete.

##### Etymology.

*Clepsis davisi* is named in honor of Dr. Donald R. Davis, collector of much of the type series, for his long and unparalleled career in Lepidoptera morphology and systematics.

##### Distribution.

*Clepsis davisi* is known from Guadeloupe and Dominica (Fig. 27). Despite extensive collecting efforts by the authors, none were found on Martinique. It could be present elsewhere in the Lesser Antilles.

##### Ecology.

Nothing is known of the biology of *Cle. davisi*. Specimens range in capture date from January to June, with a single specimen having been collected in November.

##### Remarks.

All of the Dominica specimens were collected as part of the Bredin-Archbold-Smithsonian Biological Survey of Dominica from 1960–1965, with the majority collected by Donald R. Davis and the late trichopterist Oliver S. Flint.

Maximum COI sequence divergence between barcoded specimens from the same island was 0%; between islands 2.1%. We were unable to find any significant differences between specimens from Dominica and Guadeloupe, so we opt to treat the populations on these two islands as a single species.

#### Clepsis peritana

Taxon classificationAnimaliaLepidopteraTortricidae

(Clemens, 1860)

2B6025C8-B84E-5A3C-95D8-0825BBC307C1

Figs 12G, H , 26 ; Austin et al. 2019: figs 3e, 4d

Smicrotes peritana Clemens, 1860: 356
Ptycholoma peritana (Clemens, 1860): Freeman 1958: 58.
Dichelia inconclusana Walker, 1863: 318
Clepsis pinaria Razowski & Becker, 2010: 22, syn. nov.

##### Diagnosis.

See Austin et al. 2019 and other *Clepsis* species diagnoses in the present paper.

##### Type material.

***Smicrotes peritana***: ***Lectotype*** ♂: “**Canada and USA**” [not examined] (ANSP). ***Dichelia inconclusana***: ***Lectotype*** ♂: “**North America**” [not examined] (BMNH). ***Clepsis pinaria***: ***Holotype*** ♀: **Cuba: Pinar [del] Río**: Sierra Rosario, 400 m, 5–15 vi [1]990, V.O. Becker, 71532 [figure examined]. Genitalia slide #413 [figured examined] (VBC, see remarks below). ***Paratypes*** (2♂♂): **Cuba**: 2♂♂, same data as holotype [photographs examined], genitalia slide #412 [figure examined] (VBC, see remarks below).

##### Additional material examined.

(16♂♂, 6♀♀) **Bahamas**: Central Abaco: 1♂, E side of S.C. Bolle Hwy., 3 mi. S of Treasure Cay Rd., 26.656294, -77.306661, 2 xi 2014, MGCL 239361 (MGCL); 1♀, same as previous except MGCL 239362 (MGCL). **Cuba: Ciego de Ávila**: 1♂, Central Baragua, H.K. Plank (USNM). 1♂, same as previous except ii 1931 (USNM). 1♂, same as previous except iii 1931. KAA diss. #0165 (USNM). 2♂♂, same as previous except iv 1931. KAA diss. #0164 (USNM). 1♀, same as previous except v 1931. KAA diss. #0167 (USNM). **Cienfuegos**: 1♀, 5 km W Topes de Collantes, 21°56.5'N, 80°2.3'W, R. Caburni, 10–11 xii 1994, D.R. Davis; KAA diss. #0168; USNMENT01480241 (USNM). **Holguín**: 1♂, Pinares de Mayari, 640 m, vii 1990, V.O. Becker (VBC). **La Habana**: 2♂♂, Havana, Baker. KAA diss. #0166 (USNM). **Pinar del Río**: 1♂, [leg.] Robert. KAA diss. #0177 (USNM). 1♂, 2♀♀, Mogote dos Hermanos, 3 km W Viñales, 7–8 ii 1981, ca. 150 m, D.R. Davis (1♂, 1♀ CUIC; remainder USNM). KAA diss. #0178(♂), USNMENT01480235 (CUIC); KAA diss. #0169(♀, USNM). 1♂, Sierra Rosario, 400 m, 4–6 x 1989, V.O. Becker (VBC). 2♂♂, same as previous except 5–15 vi 1990 (VBC). **Santiago de Cuba**: 1♂, Turquino, 470 m, 27/9 vii 1990, V.O. Becker (VBC). 1♂, 1♀, Santiago [de Cuba], vi [19]02, W. Schaus, 1905–244. KAA diss. #0170(♂) (BMNH).

##### Description.

See Austin et al. 2019.

##### Distribution.

Widespread in North America, Cuba, and two records from Central Abaco in The Bahamas (Fig. 26, Florida records omitted).

##### Ecology.

See Austin et al. 2019.

##### Remarks.

We treat *Clepsis pinaria* Razowski & Becker, 2010 as a junior synonym of *Clepsis peritana* (Clemens, 1860) because both the male and female genitalia are indistinguishable from dissected specimens of *Clepsis peritana* from both the United States and The Bahamas. Maximum COI sequence divergence of barcoded Cuban *Cle. peritana* was 2.8% (n = 2), which is high, but not unusual for *Cle. peritana*.

In their diagnosis of the female, Razowski & Becker compare *Cle. pinaria* to *Cle. naucinum* Razowski, 1990 from Costa Rica, mentioning that *Cle. pinaria* differs from *Cle. naucinum* in lacking a signum, despite the fact that females of *Cle. naucinum* are unknown (Razowski 1990). The males are very similar.

Razowski and Becker (2010) state that the specific epithet of *Clepsis pinaria* is derived from the “Pinar River,” but no such river exists. The holotype data label reads “Pinar Rio,” referring to the province of Pinar del Río. Coordinates for the type locality are not given on the data label, but the type locality lies somewhere in the Sierra del Rosario of western Cuba. The holotype of *Clepsis pinaria* is listed as a female in the original description, but the male genitalia illustrated are captioned as being the holotype. The photograph of the adult specimen in Razowski and Becker (2010) is of the female. Both the holotype and one male paratype were found in ISEZ, not in VBC as listed in Razowski and Becker (2010). The additional male paratype is likely in ISEZ as well. The female specimen found in ISEZ bears a red holotype label. For these reasons, we interpret the caption for the male genitalia be an error and the holotype of *Clepsis pinaria* to be female.

#### Clepsis peroniae sp. nov.

Taxon classificationAnimaliaLepidopteraTortricidae

0A445A34-830F-549D-9D73-65CC24A9CE80

http://zoobank.org/A7172EF4-7AE7-4C1B-B407-76EF36C004C0

Figs 12I, J , 21C , 26

##### Diagnosis.

*Clepsis peroniae* can be separated from all other Caribbean *Clepsis* by its loosely coiled ductus bursae (Fig. 20C). All other known Caribbean *Clepsis* possess a tightly coiled ductus bursae. In addition, *Cle. peroniae* is unique among female *Clepsis* on Hispaniola by possessing a strongly contrasting median fascia and subapical blotch (Fig. 12I, J). Males are unknown (but see remarks below).

##### Type material.

***Holotype*** ♀: **Dominican Republic: La Estrelleta [Elías Piña**]: 4 km SE Rio Limpio, ca. 760 m, 24–25 v 1973, Don & Mignon Davis; KAA diss. #0140; USNMENT01480234 (USNM). HOLOTYPE ♀ *Clepsis peroniae* Austin & Dombroskie, 2020 [typed red label] (USNM).

##### Description.

**Male.** Male unknown.

**Female (n = 1). *Head*.** Scales on vertex and frons uniformly warm brown. Labial palpus approximately 2 × width of compound eye. Scaling on lateral surface of labial palpus straw yellow pale scattered pale brown scales; medial surface of labial palpus straw yellow. Scape concolorous with vertex. Dorsal scales of flagellum with alternating rows of straw yellow basal scales and dark brown apical scales. Sensillae approximately 0.5 × width of flagellomere, porrect. ***Thorax*.** Dorsum of pro- and meso-thorax with scaling concolorous with vertex; tegulae similar. Foreleg with lateral surface femur and tibia golden brown, tarsus dark brown, nearly black, medial surface straw yellow; midleg similar to foreleg but lateral surfaces of femur and tibia straw yellow, tibial spurs dark brown on lateral surface, pale yellow medially; lateral surface of hindlegs not observed due to positioning, medial surface pale yellow, tarsi dark brown. Dorsal surface of forewing (Fig. 12I, J) with ground color golden brown, but heavily suffused with ashy gray scaling as to obscure much of ground color; median fascia dark brown, bordered by golden brown scales basally, narrowing considerably towards inner margin, widening slightly along inner margin; subapical blotch dark brown with two small patches of ashy gray scales present, bordered with golden brown scales; fringe with short ashy gray scales and long golden brown scales; FWL 6.0 mm. Dorsal surface of hindwing difficult to see owing to specimen not being spread, but appears to be dark brown with distinct strigulae; fringe with short scales concolorous, long scales off-white to pale yellow. Ventral surface of forewing brown without any obvious markings; ventral surface of hindwings pale brown with distinct strigulae. ***Abdomen*.** Vestiture not noted prior to dissection. Genitalia (Fig. 21C) with papillae anales triangular, broadest apically; apophyses posteriores approximately 0.5 × length of sternum VII, straight; apophyses anteriores approximately 0.67 × length of sternum VII, straight; sterigma relatively narrow, quadrate, well-sclerotized laterally, with shallow depression ventromesally near ostium; antrum moderate; colliculum not entire, unsclerotized ventrally; ductus bursa only loosely coiled (so much so that appears to be not coiled at all); cestum absent; ductus seminalis arising at base of ductus bursae; corpus bursae relatively small, oblong; signum robust, sickle-shaped; capitulum absent.

##### Etymology.

This species is named in memory of Dr. Patricia “Pat” Peroni (1956–2019), professor of biology at Davidson College, for her support, encouragement, and mentorship of KAA.

##### Distribution.

*Cle. peroniae* is known exclusively from the type locality in the western Cordillera Central of the Dominican Republic (Fig. 26). Elevation of the examined specimen was approximately 760 m.

##### Ecology.

Nothing is known of the biology of *Cle. peroniae*. The holotype was collected in May.

##### Remarks.

In the genitalia of one unusual CMNH male from the Sierra de Neiba range (KAA diss. #0060, KAA_DNA_0066), the shape of the phallus and cornuti are unlike any known *Clepsis* from the Caribbean. COI barcode sequence divergence between this male and the holotype of *Cle. peroniae* is 2.55%, much closer than any other barcoded *Clepsis* from Hispaniola, but divergent enough for us to question its conspecificity.

#### Rubropsichia

Taxon classificationAnimaliaLepidopteraTortricidae

Razowski, 2009

92AAAFC2-F036-56D5-A0AD-6D4B9ED81564

##### Type species.

*Rubropsichia brasiliana* Razowski, 2009

*Rubropsichia* Razowski, 2009: 240

##### Redescription.

As in species account below. See Razowski (2009) for the original diagnosis for *Rubropsichia*, in which more than one species was examined.

#### Rubropsichia santaremana

Taxon classificationAnimaliaLepidopteraTortricidae

Razowski, 2009

9D407710-4120-5C01-8655-7DA63872DF3F

Figs 13A , 20D , 26

Rubropsichia santaremana Razowski, 2009: 242

##### Diagnosis.

*Rubropsichia santaremana* is unique among *Rubropsichia* in possessing small, cap-like fused socii (Fig. 20D). In *R. brasiliana* Razowski and *R. fuesliniana* (Stoll), this structure is massive and “mushroom-shaped” (Razowski 2009). *Rubropsichiakartaboana* Razowski differs from *R. santaremana* in having elongate, fused socii.

**Figure 13.**
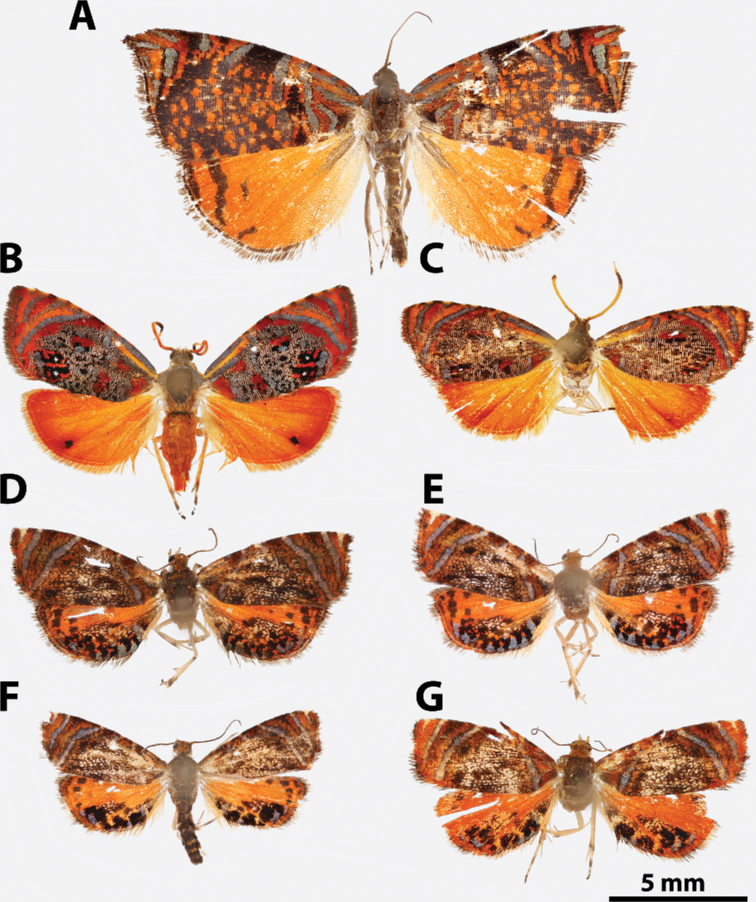
*Mictopsichia* group of genera. **A***Rubropsichia santaremana*, ♂, Grenada (BMNH) **B***Mictocommosis lesleyae* sp. nov. paratype, ♀, Dominican Republic (CUIC) **C***Mictocommosis lesleyae* sp. nov. holotype ♂, Dominican Republic (CMNH). **D***Mictopsichia cubae* ♂, Dominican Republic (CMNH) **E***Mictopsichia cubae* ♀, Dominican Republic (CMNH) **F***Mictopsichia nyhllinda* sp. nov. holotype ♂, Dominican Republic **G***Mictopsichia nyhllinda* sp. nov. paratype ♀ Dominican Republic (CMNH).

##### Type material.

***Holotype*** ♂: **Brazil: [Pará**]: Santarém, v 1919, S.M. Klages leg., Acc. 6324 [figure examined], genitalia slide #12367 [figure examined] (CMNH).

##### Additional material examined.

(1♂) **Grenada**: 1♂, 20 iv 1968, C. deWorms. KAA diss. #0133 (BMNH).

##### Redescription.

**Male (n = 1). *Head*.** Head, compound eyes small. Scales on vertex mostly missing, a few thin orange scales present; scales on frons black. Labial palpus short, approximately width of compound eye, scales on lateral and medial surface entirely black. Scape black; dorsal scales of flagellum entirely orange; sensillae 0.5 × width of flagellomere, nearly porrect. Ocellus prominent, separated from compound eye by approximately width of ocellus. Chaetosemata sparse, short, approximately 0.5 × length of orange scales on vertex. ***Thorax*.** Dorsum of pro- and mesothorax metallic silver with orange longitudinal streaks; dorsum of metathorax with pale yellow and pale orange scaling; tegulae metallic silver with lateral orange scaling. Foreleg short, with black and orange scaling on lateral surface, tarsi entirely black; midleg and hindleg, with lateral surface shining pale gray, tarsi black; medial surface of all legs pale yellow to white. Forewing (Fig. 13A) broad, acutely hooked at apex, costa evenly curved throughout entire length; FWL 9.0 mm. Dorsal surface of forewing with basal third orange with broad silver and black streaks; distal two-thirds dark gray with dense orange speckling; area near apex orange with short silver streaks. Fringe with short scales black, longer scales silver-gray. Dorsal surface of hindwing orange with gray scales present near base; dark gray to black streaks present near apex; fringe similar to forewing fringe. Ventral surface of hindwing orange with dark gray scaling present along costa and outer margin. Ventral surface of hindwing as on dorsal surface. ***Abdomen*.** Vestiture dark gray with pale orange scales present on the posterior edge of each segment. Genitalia (Fig. 20D) with uncus small, hidden behind socii; socii fused into small cap-like structure, with dense, long setae; gnathos obsolete; tegumen short, moderate; transtilla obsolete; valvae elongate, thin, curved; deciduous setae present on ventral edge near apex; cucullus thin, broadened slightly at 0.33 × length; caudal lobe of sacculus pronounced, forming a right angle, with long, thin setae present on surface; basal cavity of valvae acutely triangular; juxta broadest at sacculus, narrow at vinculum, shallow notch present where phallus rests; vinculum deep, U-shaped; phallus irregular, large, narrow, rounded at apex, small nub present near base; cornuti not observed.

##### Description.

**Female.** Female unknown.

##### Distribution.

*Rubropsichia santaremana* was previously known from a single specimen from Santarém in northern Brazil. It is now reported from Grenada (Fig. 27). The two localities are approximately 1800 km apart.

##### Ecology.

Nothing is known of the biology of *R. santaremana*. Judging by its reduced compound eyes, large ocelli, and telechromatic coloration, it is probably diurnal, like other members of the *Mictopsichia* group of genera. It may also come to lights.

##### Remarks.

This is the first record of *Rubropsichia* in the Caribbean. The other three species in the genus are known from northern South America, so this new record is not too surprising (*R. brasiliana* Razowski, 2009, TL: São Paulo de Olivença, Amazonas, Brazil; *R. fuesliniana* [Stoll, 1781], TL: Surinam; *R. kartaboana* Razowski, 2011, TL: Bartica, Guyana).

There are subtle differences in the forewing and genitalia of the Grenada specimen compared to the holotype from Brazil. Most noticeably, the terminal two rows of orange spots near the termen of the forewing are fused in the Brazilian specimen, but separate in the Grenadian specimen. In the genitalia, the basal cavity is more elongate and ventral process on the dorsal margin of the valva is more prominent in the Grenada specimen compared to the holotype. Despite these differences, we choose not to describe Grenada specimen as new, owing to the limited material available.

**Figure 14.**
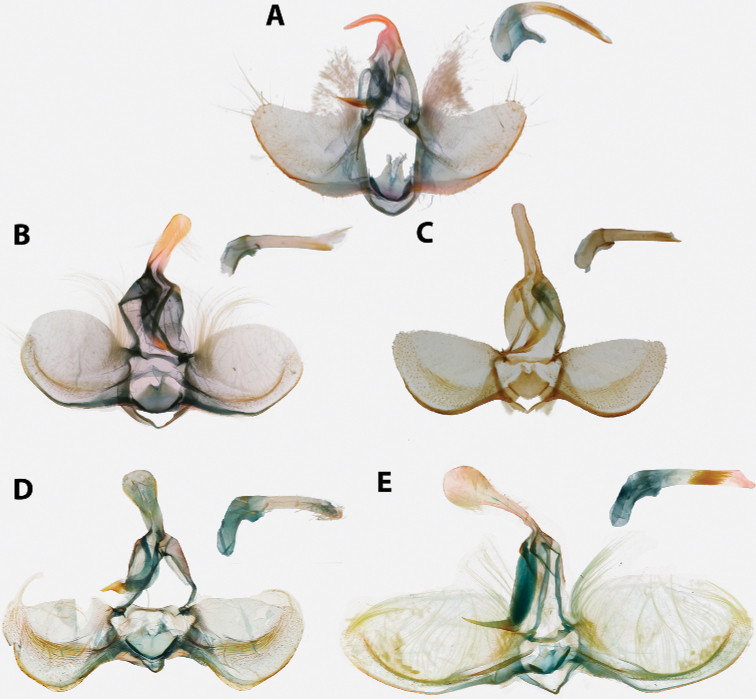
*Argyrotaenia* male genitalia. Not to scale. **A***A. ceramica*, Dominican Republic. KAA diss. #0089 (CMNH) **B***A. browni* sp. nov. holotype, Dominican Republic. KAA diss. #0097 (CMNH) **C***A. cubae*, Cuba. KAA diss. #0162 (VBC) **D***A. nuezana*, Dominican Republic. KAA diss. #0028 (CMNH) **E***A. felisana*, Dominican Republic. KAA diss. #0045 (CUIC).

**Figure 15.**
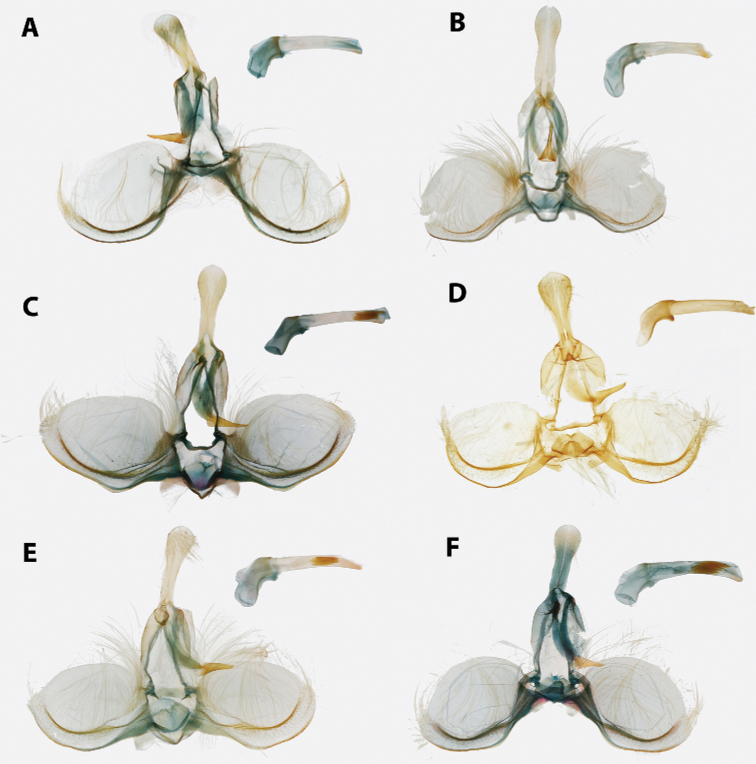
*Argyrotaenia* male genitalia. Not to scale. **A***A. bisignata* paratype, Dominican Republic. KAA diss. #0044 (CMNH) **B***A. jamaicana*, Jamaica. KAA diss. #0131 (USNM) **C***A. razowskiana* sp. nov. paratype, Dominican Republic. KAA diss. #0104 (CUIC) **D***A. cryptica praeteritana* ssp. nov. holotype, Dominican Republic. Razowski diss. #10732 (CMNH) **E***A. cryptica cryptica* ssp. nov. paratype, Dominican Republic. KAA diss. #0115 (CMNH) **F***A. paradisei* sp. nov. paratype, Dominican Republic. KAA diss. #0116 (CMNH).

#### Mictocommosis

Taxon classificationAnimaliaLepidopteraTortricidae

Diakonoff, 1977

EA74D73A-B65D-5E28-96CA-FCE90514FA9F

##### Type species.

*Simaethis nigromaculata* Issiki, 1930

*Mictocommosis* Diakonoff, 1977: 8

##### Description.

As in species description below. *Mictocommosis lesleyae* may not be conspecific with *Simaethis nigromaculata* Issiki, 1930, the type species of *Mictocommosis* (see remarks under species account below).

**Figure 16.**
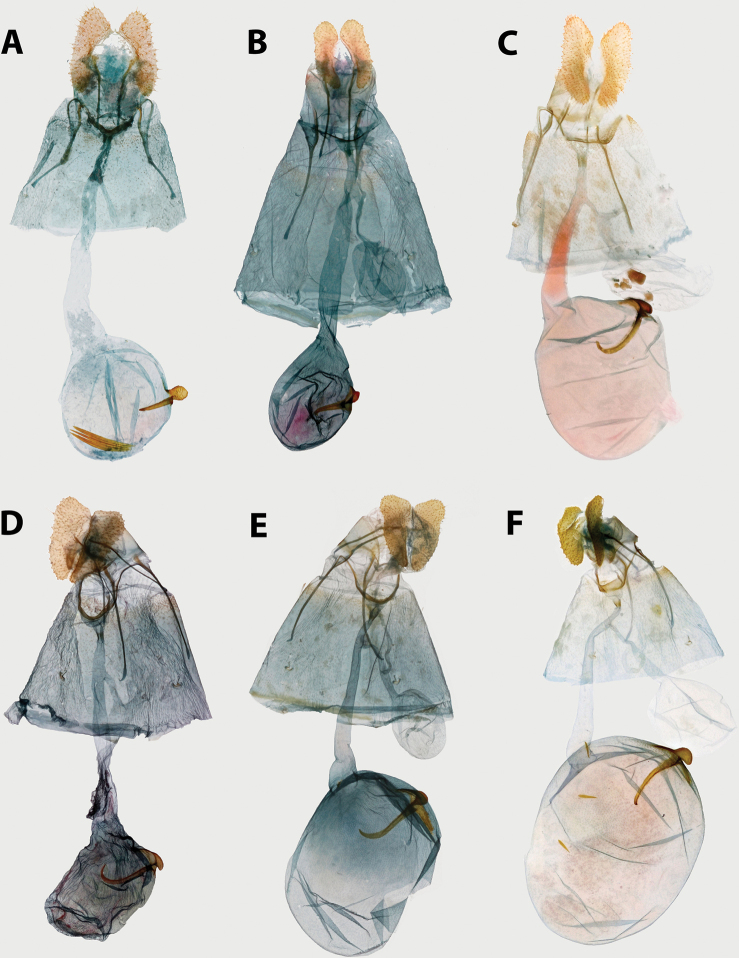
*Argyrotaenia* female genitalia. Not to scale. **A***A. ceramica*, Dominican Republic. KAA diss. #0083 (CMNH) **B***A. browni* sp. nov. paratype, Dominican Republic. KAA diss. #0099 (CUIC) **C***A. cubae*, Cuba. KAA diss. #0163 (VBC) **D***A. nuezana*, Dominican Republic. KAA diss. #0024 (CMNH) **E***A. felisana*, Dominican Republic. KAA diss. #0081 (CMNH) **F***A. bisignata*, Dominican Republic. KAA diss. #0054 (CMNH).

#### Mictocommosis lesleyae sp. nov.

Taxon classificationAnimaliaLepidopteraTortricidae

2F461106-A9C1-5A37-B270-CC1E279226D2

http://zoobank.org/63544901-504C-4F8B-9DBA-7952A18CF40E

Figs 13B, C , 20E , 21E , 27

##### Diagnosis.

Wing pattern alone is sufficient to identify *Mictocommosis lesleyae* (Fig. 13B, C). It lacks the complex scaling patterns on the dorsal surface of the hindwing present in *M. godmani* (Walsingham, 1914), the only other species of *Mictocommosis* currently recognized in the Neotropics (but see remarks below).

##### Type material.

***Holotype*** ♂: **Dominican Republic: Azua**: East side of crest, Sierra Martin Garcia, 7 km WNW Barrero. 18°21'N, 70°58'W, 860 m, 25–26 vii 1992, cloud forest adjacent to disturbed forest, C. Young, R. Davidson, S. Thompson, J. Rawlins. KAA diss. #0173. HOLOTYPE *Mictocommosis lesleyae* Austin & Dombroskie [typed red label] (CMNH). ***Paratypes*** (3♀♀): **Dominican Republic: Azua**: 1♀, same data as holotype. KAA diss. #0174 (CMNH). **Hato Mayor**: 1♀, Parque Los Haitises, 3 km W Cueva de Arena, 19°04'N, 69°29'W, 20 m, 7–9 vii 1992, mesic lowland forest, R. Davidson, J. Rawlins, S. Thompson, C. Young. KAA diss. #0175 (CMNH). 1♀, Parque Los Haitises, near Cueva de Arena, 19°04'N, 69°28'W, 10 m, 7–9 vii 1992, coastal vegetation on limestone, C. Young, R. Davidson, S. Thompson, J. Rawlins, KAA_DNA_0053 (CUIC). All paratypes affixed with the following typed blue label: PARATYPE ♂/♀ *Mictocommosis lesleyae* Austin & Dombroskie, 2020.

##### Description.

**Male (n = 1). *Head*.** Scales on vertex thin, leaden gray, pale orange-yellow laterally. Scales on frons concolorous, but absent ventrally. Labial palpus approximately 1.5 × width of compound eye, thin; scales absent laterally and medially, but white ventrally. Proboscis naked at base, fine setae present laterally. Scape leaden gray dorsally, pale yellow to orange-yellow ventrally. Antenna massively thickened, slightly compressed laterally; sensillae approximately width of flagellomere, tightly appressed. Dorsum of flagellum with one row of scales per segment; orange-yellow to 0.8 × length of antennae, then dark gray for 0.1 ×, then pale yellow for 0.1 ×, terminal segment dark gray; ventral surface of flagellum naked. Ocellus large, separated from compound eye by approximately 0.5 × width of ocellus. Chaetosemata 0.5–2 × length of scales on vertex. ***Thorax*.** Dorsum of pro- and meso-thorax ashy gray, intermixed with orange-yellow scales; dorsum of metathorax orange-yellow; tegulae leaden gray. Foreleg with lateral surface with ashy gray scaling, tarsi intermixed with white scales; midleg pale yellow, with ashy gray scales restricted to tarsi; hindlegs missing; medial surface of legs pale yellow to white. Forewing (Fig. 13C) broad, costa evenly and gently curved throughout entire length; FWL 6.0 mm. Dorsal surface of forewing beautiful, unmistakable, with ground color deep red-orange, heavily suffused with orange-yellow; two silver lines running parallel to costa from base to 0.33 × length of costa, separated by an equally-wide yellow streak; median area of forewing heavily peppered with brilliantly bicolored scales, (ashy gray basally, white terminally), patches of black scales scattered apically; three silver fasciae present towards outer margin: most basal of three extending from 0.6 × length of costa to just below termen, composed of silver scales, separated from next fascia by an orange gap; next fascia faint, composed primarily of same bicolored present in medial area of forewing, separated from terminal fascia by deep red scales; terminal fascia extending from 0.8 × length of costa to 0.5 × length of outer margin, composed of silver scales; orange scales beyond to fringe. Fringe with short scales red-orange, long scales silver. Dorsal surface of hindwing orange, becoming slightly darker apically; fringe concolorous, longer scales slightly paler. Ventral surface of forewing orange, with scattered black scales in median area. Ventral surface of hindwing concolorous with dorsal surface. ***Abdomen*.** Vestiture orange, leaden gray at base. Genitalia (Fig. 20E) with uncus broad, well-developed, expanded to broad, flattened apex with shallow notch, covered in robust spines on lateral edge of neck near apex and apex itself; socii well-developed as large pads thinly-connected to tegumen, densely covered in thick spines; tegumen and gnathos weak, gnathos without terminal plate; transtilla weak with long medial process; valvae elongate, triangular; sacculus with spine-like extension extending into basal cavity; juxta with shallow notch; phallus short, rounded basally, downturned and deeply-notched apically, with blunt, thorn-like cornutus.

**Female (n = 3). *Head*.** As in male except lateral surface of labial palpus with scaling pale yellow to white. ***Thorax*.** As in male except hindlegs with femur and tibia pale yellow to orange, tarsi leaden gray and white. Dorsal surface of forewing (Fig. 13B) with small white patches of scales in center of two of black patches in median area of forewing, which could be interpreted as false eye spots, FWL 6.0–6.5 mm (mean = 6.3; n = 3). Dorsal surface of hindwing with a few silver scales on costal edge; a small black patch of scales along Cu2 near fringe in two of three paratypes, a dark patch of deep red-orange scales in same area of third paratype; frenulum with three or four bristles. ***Abdomen*.** Vestiture as in male. Genitalia (Fig. 21E) with papillae anales triangular posteriorly, anterior lobe narrowed; apophyses both approximately 0.75 × length of sternum VII; sterigma broad, quadrate, well-sclerotized, covered in minute spines; colliculum a small sclerotized plate; ductus bursae uniform in width throughout, twice-coiled; ductus seminalis arising near base of ductus bursae; corpus bursae large, ovoid; signum a short, rounded nub; with long, paired scobinate extensions of finely-spined basal plate to bottom of corpus bursae; capitulum absent.

##### Etymology.

This beautiful species is named in honor of KAA’s mother, Lesley, for her unwavering support and love.

##### Distribution.

*Mictocommosis lesleyae* is known from two localities in the Dominican Republic (Fig. 27): at high elevation on Sierra Martin Garcia in the south and at low elevation in Parque Nacional de Los Haitises on the northern coast.

##### Biology.

Nothing is known of the biology of *Mictocommosis lesleyae*. Like other members of the *Mictopsichia* group of genera, it is presumed to be diurnal but may also come to lights. The four known specimens were collected in July.

##### Remarks.

Unfortunately, the phallus of the holotype was lost prior to slide mounting. With the description of *Mictocommosis lesleyae*, there are now two described species of *Mictocommosis* in the Neotropics. Two more, *Mictopsichia ornatissima* (Dognin, 1909) and *Mictopsichia buenavistae* Razowski, 2009 may also belong to this group. *Mictopsichia ornatissima* was not examined nor dissected by Razowski (2009) and appears to be closely related to *Mictocommosis godmani* (Walsingham, 1914), a possible relationship which was alluded to by Walsingham. *Mictopsichia buenavistae* is known only from a female. Its genitalia are similar to known females of *Mictocommosis* and its similarity to *Mictopsichia ornatissima* was noted by Razowski (2009). Whether or not these Neotropical species truly belong to *Mictocommosis* remains to be seen. We believe it is unlikely, as the type species of *Mictocommosis* (*Simaethis nigromaculata* Issiki, 1930) was described from Japan and possesses a basally scaled proboscis (Diakonoff 1977), a character not seen in *Mictocommosis lesleyae*, nor any other known tortricid genus with the exception of *Thaumatographa* Walsingham (Diakonoff 1977). One additional species, *Mictopsichia jamaicana* Razowski, 2009, may also belong to this group of Neotropical “*Mictocommosis*”, but we were unable to examine the holotype and thus choose to retain it in *Mictopsichia*.

**Figure 17.**
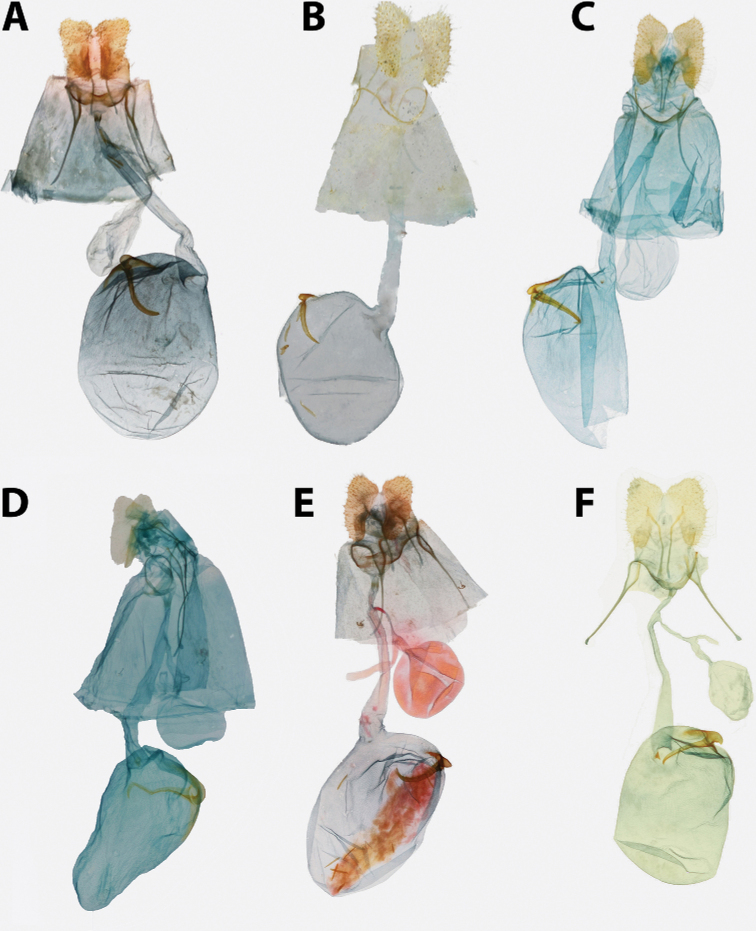
*Argyrotaenia* female genitalia. Not to scale. **A***A. jamaicana*, Jamaica. KAA diss. #0127 (USNM) **B***A. vinalesiae*, Cuba. KAA diss. #0159 (VBC) **C***A. razowskiana* sp. nov. paratype, Dominican Republic. KAA diss. #0106 (CMNH) **D***A. paradisei* sp. nov. paratype, Dominican Republic. KAA diss. #0073 (CUIC) **E***A. cryptica cryptica* ssp. nov. paratype, Dominican Republic. KAA diss. #0171 (CMNH) **F***A. cryptica praeteritana* ssp. nov. paratype, Dominican Republic. Razowski diss. #10733 (CMNH).

#### Mictopsichia

Taxon classificationAnimaliaLepidopteraTortricidae

Hübner, [1825] 1816

ADFC0523-BDFC-58B9-8F42-B2DFB47A27FC

##### Type species.

Phalaena (Tortrix) hubneriana Stoll, 1791

*Mictopsichia* [1825] 1816: 374

*Micropsichia* Agassiz, 1848 (misspelling): 674

*Micropsychia* Agassiz, 1848 (misspelling): 674

*Mictopsychia* Riley, 1889 (misspelling): 158

*Mictropsichia* Heppner, 1978 (misspelling): 53

##### Remarks.

The following description is specific to the two species of Caribbean *Mictopsichia*. Some characters mentioned may not apply to *Mictopsichia jamaicana* Razowski, 2009, which we were unable to examine. The majority of *Mictopsichia*, including the Caribbean species, may not be conspecific with Phalaena (Tortrix) hubneriana, the type species of *Mictopsichia* Hübner, [1825] 1816. See the comments below the *Mictopsichia* key and remarks under *Mictocommosis lesleyae*.

##### Redescription.

Labial palpus approximately width of compound eye; ocellus large, separated from reduced compound eye by approximately width of ocellus; chaetosemata 0.25–0.75 × length of scales on vertex; dorsal surface of metathorax with dark silver scaling; foreleg significantly shorter than midleg and hindleg; forewing and hindwing pattern (Fig. 13D–G; Razowski 2009: fig. 55) unlikely to be confused with any other Caribbean tortricid. Male genitalia (Fig. 20F, G) with uncus obsolete, socii composed of dorsally setose, acutely-pointed processes; gnathos composed of broad, laterally-rounded, quadrate mesal process; transtilla obsolete; valvae elongate and densely setose, with broad, scale-like setae present along ventral margin, thin hair-like setae scattered over entire surface, but most densely clustered along dorsal margin at 0.5 × length; submedian belt with several tooth-like dorsal projections. Female genitalia (Fig. 21F, G; Razowski 2009: fig. 39) with papillae anales slender (triangular in *Mictopsichia jamaicana*), elongate, laterally-directed anteriorly; sterigma broad posteriorly, much more constricted and very deep anteriorly (except in *Mictopsichia jamaicana*); ostium similar in width to anterior portion of sterigma, colliculum a uniformly broad, ring-like structure (absent in *Mictopsichia jamaicana*); ductus bursae of uniform width; ductus seminalis arising at base of ductus bursae; sterigma well-developed, with or without parallel distinct rows of scobinations along wall of ductus bursae; capitulum absent; elongate basal plate present in *Mictopsichia jamaicana*.

**Figure 18.**
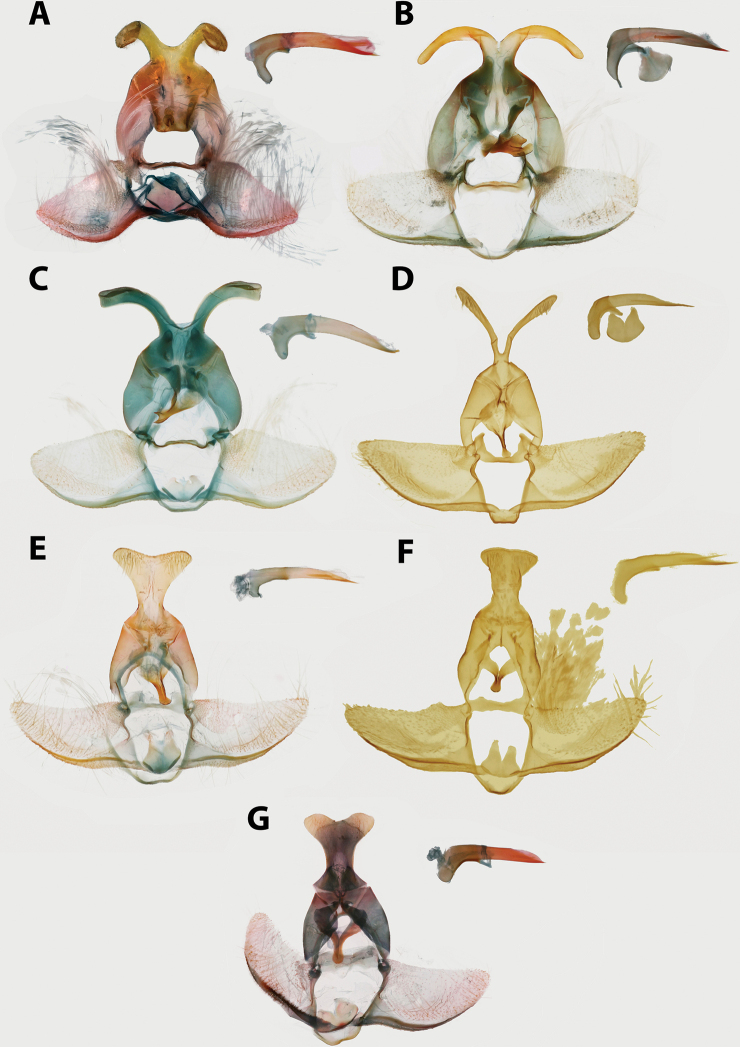
*Claduncaria* male genitalia. Not to scale. **A***Cla. maestrana*, Cuba. KAA diss. #0150 (VBC) **B***Cla. ochrochlaena*, Dominican Republic. KAA diss. #0120 (CMNH) **C***Cla. rawlinsana* sp. nov. paratype, Dominican Republic. KAA diss. #0121 (CUIC) **D***Cla. rufochlaena* holotype, Jamaica. Razowski diss. #12275 (CMNH) **E***Cla. mesosignaria*, Dominican Republic. KAA diss. #0112 (CMNH) **F***Cla. minisignaria*, Dominican Republic. Razowski diss. #10703 (CMNH) **G***Cla. taino* sp. nov. paratype, Dominican Republic. KAA diss. #0119 (CMNH).

### Key to species of Caribbean *Mictopsichia*^4^

**Table:** 

1	FW with distinct black tornal patch; hindwing entirely orange (Razowski 2009: fig. 55); Jamaica	***M. jamaicana***
–	FW without distinct black tornal patch; hindwing with distinct patches of silver-blue and black scales (Fig. 13D–G)	**2**
2	Dorsal surface of thorax with two slender transverse bands of orange scaling; dorsal surface of hindwing with more extensive silver-blue and black scaling (Fig. 13D, E); male genitalia with valva narrowing apically (Fig. 20F); female genitalia with signum robust, two distinct parallel lines of scobinations present along wall of corpus bursae (Fig. 21F); Cuba, Hispaniola, Costa Rica, Honduras	***M. cubae***
–	Dorsal surface of thorax entirely silver; dorsal surface of hindwing with less extensive silver-blue and black scaling (Fig. 13F, G); male genitalia with valva not narrowing apically (Fig. 20G); female genitalia with signum thin, slightly irregular, without distinct parallel lines of scobinations along wall of corpus bursae (Fig. 21G); Hispaniola, Cuba	***M. nyhllinda* sp. nov.**

**Comments.**Phalaena (Tortrix) hubneriana Stoll, 1791, the type species of *Mictopsichia*, is significantly different from all subsequently described species in *Mictopsichia* in both wing pattern and male genitalia. This was alluded to by Razowski (2009), but unfortunately not given adequate discussion in his papers. Hence, the vast majority of *Mictopsichia* may be require the description of a new genus. We choose to provisionally treat the following species as *Mictopsichia* because describing a new genus for several non-Caribbean species is beyond the scope of this paper. The male of *M. jamaicana* is unknown.

The only host record for the genus is from a series of four specimens from Venezuela in USNM identified as *Mictopsichia gemmisparsana* and reportedly reared from *Vitis vinifera* Linnaeus (Matthews et al. 2011). Matthews et al. (2011) suggested *Mictopsichia* may use their metallic markings as a startle or mimicry display to escape jumping spider predators. Similar markings and behavior have been observed in many other insect lineages (Rota and Wagner 2006; Hill et al. 2019).

**Figure 19.**
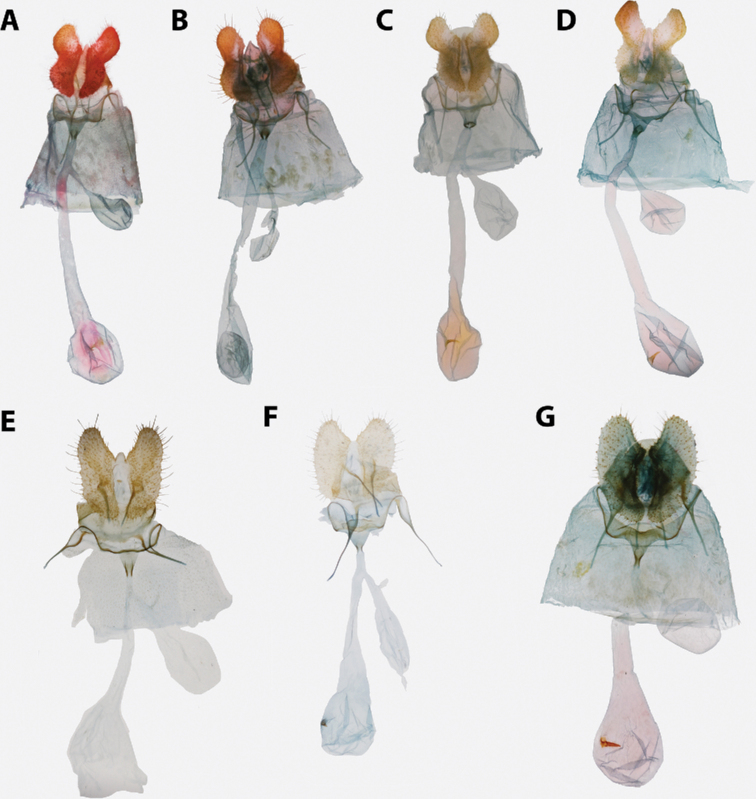
*Claduncaria* female genitalia. Not to scale. **A***Cla. maestrana*, Cuba. KAA diss. #0154 (VBC) **B***Cla. ochrochlaena*, Dominican Republic. KAA diss. #0126 (CMNH) **C***Cla. rawlinsana* sp. nov. paratype, Dominican Republic. KAA diss. #0122 (CMNH) **D***Cla. praedictana* sp. nov. holotype, Dominican Republic. KAA diss. #0123 (CMNH) **E***Cla. chalarostium* comb. nov., stat. nov. holotype (erroneously labeled as paratype of *Argyrotaenia jamaicana*), Jamaica. Razowski diss. #12273 (CMNH) **F***Cla. minisignaria* holotype, Dominican Republic. Razowski diss. #10700 (CMNH) **G***Cla. mesosignaria*, Dominican Republic. KAA diss. #0108 (CUIC).

#### Mictopsichia cubae

Taxon classificationAnimaliaLepidopteraTortricidae

Razowski, 2009

66386152-1BBD-5C61-AF6D-ADA55193888A

**Figure 20.**
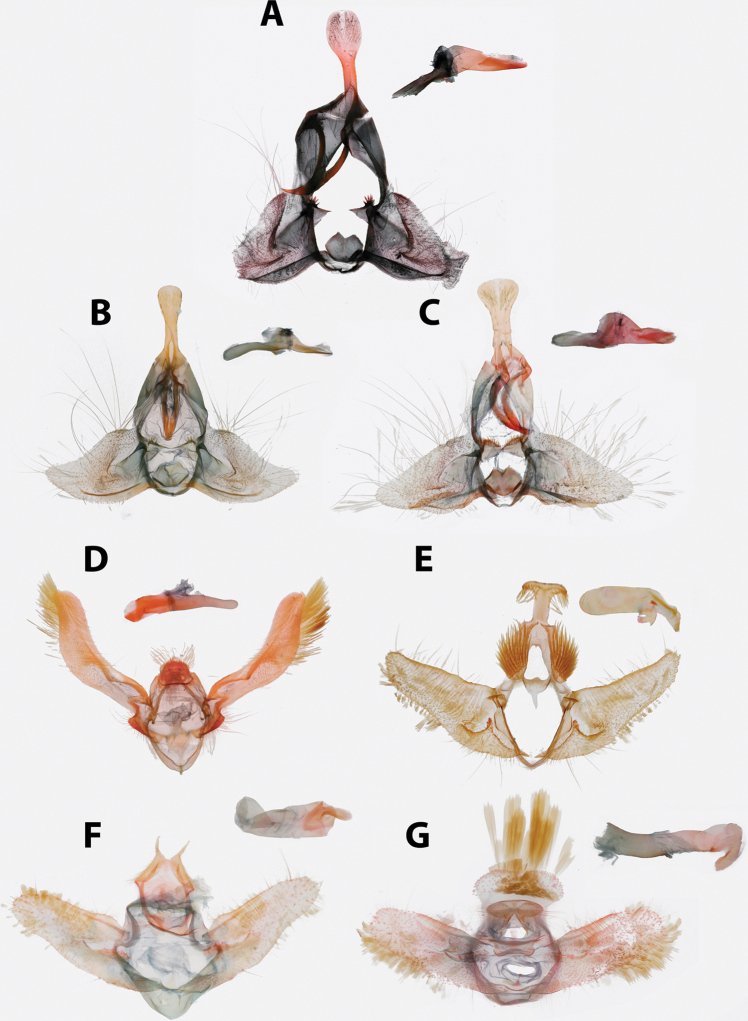
*Clepsis* and *Mictopsichia* group of genera male genitalia. Phallus inset. Not to scale. **A***Cle. deroni* sp. nov. paratype, Dominican Republic. KAA diss. #0057 (CMNH) **B***Cle. jamesstewarti* sp. nov. paratype, Dominican Republic. KAA diss. #0143 (CMNH) **C***Cle. davisi* sp. nov. paratype, Guadeloupe. KAA diss. #0184 (CUIC) **D***Rubropsichia santaremana*, Grenada. KAA diss. #0133 (BMNH) **E***Mictocommosis lesleyae* sp. nov. holotype, Dominican Republic. KAA diss. #0173 (CMNH) **F***Mictopsichia cubae*, Dominican Republic. KAA diss. #0130 (CMNH) **G***Mictopsichia nyhllinda* sp. nov. holotype, Dominican Republic. KAA diss. #0200; scaled tergite VIII left intact (CMNH).

**Figure 21.**
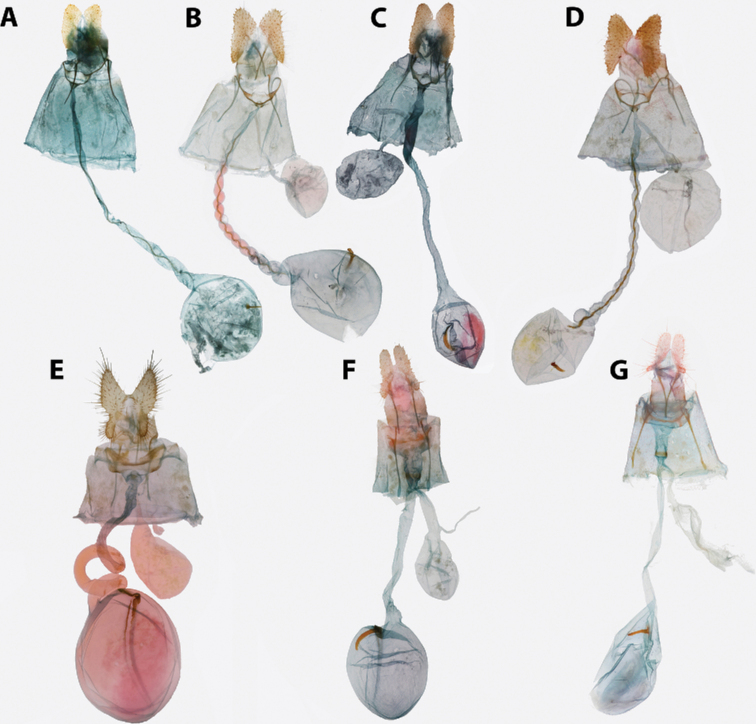
*Clepsis* and *Mictopsichia* group of genera female genitalia. Not to scale. **A***Cle. deroni* sp. nov. holotype, Dominican Republic. KAA diss. #0058 (CMNH) **B***Cle. jamesstewarti* sp. nov. holotype, Dominican Republic. KAA diss. #0149 (CMNH) **C***Cle. peroniae* sp. nov. holotype, Dominican Republic. KAA diss. #0140 (USNM) **D***Cle. davisi* sp. nov. holotype, Guadeloupe. KAA diss. #0183 (CUIC) **E***Mictocommosis lesleyae* sp. nov. paratype, Dominican Republic. KAA diss. #0175 (CMNH) **F***Mictopsichia cubae*, Dominican Republic. KAA diss. #0196 (CMNH) **G***Mictopsichia nyhllinda* sp. nov. paratype, Dominican Republic. KAA diss. #0199 (CMNH).

Figs 13D, E , 20F , 21F , 25C

Mictopsichia cubae Razowski, 2009: 227

##### Diagnosis.

In the Caribbean, *Mictopsichia cubae* (Fig. 13D, E) is most likely to be confused with *M. nyhllinda* (Fig. 13F, G). From this species it differs in possessing two slender transverse bands of orange scaling on the dorsal surface of the thorax, a feature absent in all examined specimens of *M. nyhllinda*. Male genitalia (Fig. 20F) differ in the shape of the valvae, with the base noticeably wider than the apex and the presence of a weakly-developed basal lobe on the dorsal margin. In *M. nyhllinda*, the valvae are even in width throughout their length and lack such a basal lobe (Fig. 20G). Female genitalia (Fig. 21F) is distinct from *M. nyhllinda* (Fig. 21G) by possessing a more robust signum and distinct parallel lines of scobinations along the wall of the corpus bursae.

##### Type material.

***Holotype*** ♂: **Cuba**: Santiago [de Cuba], ii [19]02, W. Schaus, 1905-244 [examined], BM genitalia slide #31697 [examined] (BMNH).

##### Additional material examined.

(2♂♂, 2♀♀) **Costa Rica: [Alajuela**]: 1♂, Área de Conservación Guanacaste, [Sector Rincon Rain Forest, Sendero Anonas, 10.9053, -85.2788, 405 m, 8 v 2013], 13-SRNP-42649, KAA diss. #0201 (USNM). 1♀, same as previous, but [31 vii 2013], 13-SRNP-41503, KAA diss. #0202 (USNM). **Dominican Republic: Hato Mayor**: 1♂, 1♀, Parque Los Haitises, 3 km W Cueva de Arena, 19°04'N, 69°29'W, 20 m, 7–9 vii 1992, mesic lowland forest, R. Davidson, J. Rawlins, S. Thompson, C. Young; KAA diss. #0130(♂), KAA_DNA_0056; KAA diss. #0196(♀), KAA_DNA_0055 (CMNH).

##### Redescription.

**Male (n = 3). *Head*** (n = 2). Scales on vertex brown and orange, long and thin. Scales on frons straw yellow and orange, appressed. Scales on lateral surface predominantly pale yellow, but intermixed with a few straw yellow and black scales. Medial surface of palpus pale yellow to white. Scape concolorous with scales on vertex. Dorsal scales of flagellum predominantly black, a few straw yellow scales interspersed. Sensillae approximately width of flagellomere, recurved. ***Thorax*.** Dorsum of pro- and mesothorax shining silver with two lateral parallel bands of orange scaling, tegulae silver with orange scaling at base. Foreleg with lateral surface with shining black scaling. Midleg and hindleg with lateral surface of femur straw yellow; lateral surface of tibia straw yellow and orange, terminal portion black; tarsi black. Medial surface of all legs pale yellow to white. Forewing (Fig. 13D) with basal half of costa straight or nearly so, distal third very gently curved; FWL 5.0–5.5 mm (mean = 5.2; n = 3). Dorsal surface of forewing dark brown, nearly black, heavily sprinkled with white in median area and suffused with orange in basal and terminal area. Two metallic blue-silver lines in basal area extending from inner margin to costa; three in terminal area, two extending from either side of tornus to costa and one very short, near apex; portion along costa white. Fringe predominantly dark gray, nearly black, with a few scattered short dark red scales; iridescent blue-purple when viewed from certain angles under light. Dorsal surface of hindwing orange, with heavy black-and-white speckling from M_2_ to A_2_ (“cubito-anal field” *sensu*Razowski 2009); black spots near apex (especially so in holotype); metallic blue-silver spots along margin. Fringe with short scales, predominantly dark gray, red-orange scales in small patches; long scales entirely gray; iridescent blue-purple when viewed from certain angles under light. Ventral surface of forewing orange with two wide dark-brown bands (weakly developed in some specimens) extending from costa to M3, bordering two longest blue-silver lines on terminal area of forewing, which are present on ventral surface as pale yellow lines. Ventral surface of hindwing orange, with large dark brown spots present along fringe. ***Abdomen*.** Vestiture with shining gray-brown scales on dorsal surface, terminal row of scales on each segment light orange; ventral surface covered in straw yellow scales. Genitalia (Fig. 20F) with uncus obsolete; socii terminally acute, with long setae projecting laterally; tegumen weak, membranous; arms of gnathos not converging, forming a broad, somewhat quadrate terminal complex, joined by a thin membrane; valvae moderate, elongate; submedian belt with four or five noticeable tooth-like projections on dorsal edge; elongate cavity present between pulvinus and submedian belt; basal cavity small, obsolete; phallus broad, rounded basally, truncate apically, with broad, spatulate extension and short non-deciduous cornutus-like thorn present at apex.

**Female (n = 2). *Head*.** As in male, except sensillae porrect, no more than 0.5 × width of flagellomere. ***Thorax*.** Dorsal surface of wings (Fig. 13E) as in male, but with fringes with more extensive short red-orange scales; FWL 5.5 mm (n = 2). Frenulum with three bristles. ***Abdomen*.** Vestiture as in male. Genitalia (Fig. 21F) with papillae anales elongate, narrow, flared anteriorly; apophyses anteriores and posteriores approximately both 1 × length of sternum VII; sterigma broad, quadrate, membranous; ostium broad, with weakly sclerotized ring-like colliculum; ductus bursae uniform in width throughout length; ductus seminalis arising at base of ductus bursae, uniform in width throughout; corpus bursae globose; signum robust, sickle-shaped, with long, scobinate extension of basal plate present as two parallel lines; capitulum absent.

##### Distribution.

*Mictopsichia cubae* is known from coastal elevations on Cuba and Hispaniola (Fig. 25C), as well at 405 m elevation from a single location in Costa Rica. Matthews et al. (2011) reported it from Honduras, but we believe only the female was correctly identified (see remarks below).

##### Ecology.

Nothing is known of the biology of *Mictopsichia cubae*. Like other members of the genus, *M. cubae* is presumed to be diurnal but may also come to lights. Examined specimens range in capture date from February to July.

##### Remarks.

The holotype is in poor condition. The head is missing, as are the legs. The right forewing is stored in a plastic capsule separate from the specimen and the wings are heavily worn and partially torn. Razowski described the head in his original description, but the holotype he figured lacks a head. Either the head was lost between his description and photography or Razowski erroneously described the head when it was lost before he examined the specimen.

One Costa Rican specimen was a 100% COI sequence matches to a pair of barcoded *M. cubae* from Hispaniola. This specimen, along with a similar one with 1.7% COI sequence divergence, represents the second report of *M. cubae* from Central America.

Matthews et al. (2011) reported *Mictopsichia cubae* from Honduras from both a male and female specimen. They obtained a 606 bp fragment of CO1 from the leg of the male specimen, but because of the age of the holotype, a comparison between the two was not conducted. This male specimen, however, is significantly different from *M. cubae* from Hispaniola and Costa Rica in both DNA barcode (10.44% sequence divergence) and genitalia morphology.

Most significantly in the male genitalia, the Honduran specimen lacks noticeable tooth-like projections on the dorsal rib of the submedian belt and a large cavity between the pulvinus and submedian belt, both of which are present in the holotype of *M. cubae* and the dissected males from Hispaniola and Costa Rica. As far as we can tell, this is not an artifact of slide-mounting. In addition, the shape of the valvae are different. In the Honduran specimen, the valvae are of almost uniform width throughout their entire length (similar to *M. nyhllinda*), whereas in the holotype, Hispaniolan, and Costa Rican specimens, the valvae are widest at the base and gradually narrow apically. Unfortunately, Matthews et a. (2011) did not figure the male beyond the genitalia and we were unable to examine the specimen for this study. This male from Honduras does not appear to be conspecific with *M. nyhllinda*, as minimum COI sequence divergence was significantly different (9.5%) from a barcoded non-type specimen from Cuba.

The female described and figured in Matthews et al. (2011), on the other hand, is a good match in both wing pattern and genitalia to *M. cubae* from Hispaniola and Costa Rica. The signum in this female is a little more robust than in the other specimens we examined but is otherwise identical. Unfortunately, it was not barcoded.

The two Honduran specimens are from two localities about 5 km apart and were collected five months apart, so it is unclear how they were associated beyond wing pattern. Many species of *Mictopsichia* are exceedingly similar in wing pattern and often occur sympatrically (KAA pers. obs.). It is upon this basis that we believe the specimens described in Matthews et al. (2011) are not conspecific and only the female represents *M. cubae*.

#### Mictopsichia nyhllinda sp. nov.

Taxon classificationAnimaliaLepidopteraTortricidae

5CCEFD2C-EAD8-5339-8D78-E5E063ECDA3F

http://zoobank.org/F1BF971D-EE5C-4CD4-88DF-43CD8B77826C

Figs 13F, G , 20G , 21G , 25C

##### Diagnosis.

*Mictopsichia nyhllinda* (Fig. 13F, G) is most likely to be confused with *M. cubae* (Fig. 13D, E). See the diagnosis for that species.

##### Type material.

***Holotype*** ♂: **Dominican Republic: Hato Mayor**: Parque Los Haitises, 3 km W Cueva de Arena, 19°04'N, 69°29'W, 20 m, 7–9 vii 1992, mesic lowland forest, R. Davidson, J. Rawlins, S. Thompson, C. Young, KAA diss. #0200 (CMNH). ***Paratype*** (♀): **Dominican Republic**: same data as holotype, KAA diss. #0199 (CMNH). Paratype affixed with the following typed blue label: PARATYPE ♀ *Mictopsichia nyhllinda* Austin & Dombroskie, 2020.

##### Additional material examined.

(5♀♀) **Cuba: Pinar del Río**: 1♀, Sierra del Rosario, 4–6 x 1989, 400 m, V. O. Becker, KAA diss. #0172, KAA_DNA_0054 (VBC). 1♀, same as previous except 5–15 vi 1990, KAA diss. #0198 (VBC). **Dominican Republic: Hato Mayor**: 2♀♀, same data as holotype (CUIC, CMNH). **Pedernales**: 1♀, Along Rio Mulito, 13 km N Pedernales, 18°09'N 71°46'W, 230 m, 17 vii 1992, riparian woodland, J. Rawlins, S. Thompson, C. Young, R. Davidson, KAA diss. #0132 (CMNH).

##### Description.

**Male (n = 1). *Head***. Scales on vertex brown, orange laterally, long and thin. Scales on frons straw yellow and orange, brown dorsally; appressed. Scales on lateral surface straw yellow, but intermixed with dark brown scales; second segment expanded ventrally. Medial surface of palpus pale yellow to white. Scape predominantly orange, a few brown scales present basally. Dorsal scales of flagellum predominantly black, a few straw yellow scales interspersed. Sensillae approximately width of flagellomere, recurved. ***Thorax*.** Dorsum of pro- and mesothorax entirely silver, tegulae silver with orange scaling at base. Foreleg with lateral surface with shining black scaling. Midleg and hindleg with lateral surface of femur straw yellow; lateral surface of tibia straw yellow and orange, terminal portion black; tarsi black. Medial surface of all legs pale yellow to white. Forewing (Fig. 13F) including fringe similar to *M. cubae*, FWL 5.0 mm. Dorsal surface of hindwing orange, with black-and-white speckling from M2 to A2 (“cubito-anal field” *sensu*Razowski 2009); black spots present near apex, but smaller and less consolidated than in *M. cubae*; metallic blue-silver spots along margin smaller and less extensive than in *M. cubae*; fringe similar to *M. cubae*. Ventral surface of forewing similar to *M. cubae*, but with more prominent dark brown bands. Ventral surface of hindwing similar to *M. cubae*. ***Abdomen*.** Vestiture similar to *M. cubae*. Genitalia (Fig. 20G) with uncus obsolete; socii joined dorsally, terminally acute, with long setae projecting from dorsal surface; tegumen weak, membranous; arms of gnathos joined, forming a broad, somewhat quadrate medial complex; transtilla obsolete; valvae moderate, parallel-sided, evenly-rounded apically, without obvious dorsal lobe on dorsal margin; submedian belt with two or three tooth-like dorsal projections; basal cavity obsolete; thin, juxta-like sclerotization present. Phallus elongate, slightly curved, with broad, spatulate extension present apically (the natural orientation of this extension may be distorted in Fig. 19G as vesica appears to have been partially everted), minute non-deciduous cornutus-like thorn present.

**Female (n = 1). *Head*.** As in male but sensillae shorter, approximately 0.5 × width of flagellomere, straight. ***Thorax*.** Thorax, legs and forewing (Fig. 13G) as in male but with even less extensive black scaling near apex of dorsal and ventral surface of hindwing. FWL 5.0 mm. Frenulum with three bristles. ***Abdomen*.** Vestiture as in male. Genitalia (Fig. 21G) with papillae anales narrow, elongate, flared anteriorly; apophyses anteriores approximately 0.75 × length of sternum VII; apophyses posteriores approximately 1 × length of sternum VII; sterigma broad, quadrate, membranous; ostium broad, with weakly sclerotized ring-like colliculum; ductus bursae uniform in width throughout length; ductus seminalis arising at base of ductus bursae, uniform in width throughout; corpus bursae globose; signum thin, slightly irregular, moderate in length, finely roughened at base, with short, almost obsolete scobinate extension of basal plate; capitulum absent.

##### Etymology.

This species is named in honor of KAA’s paternal grandparents, Nyhl and Linda Austin, for their unwavering support and love for their children and grandchildren.

##### Distribution.

*Mictopsichia nyhllinda* is known from Hispaniola and Cuba (Fig. 25C).

##### Ecology.

Nothing is known of the biology of *M. nyhllinda*. Like other species of the *Mictopsichia* group of genera, *M. nyhllinda* is presumed to be diurnal but may also come to lights. Examined specimens range in capture date from July to October.

##### Remarks.

Unfortunately, the phallus of the holotype was lost prior to slide mounting. We choose to exclude five female specimens of *M. nyhllinda* from the type series because of lack of barcoding information for Hispaniolan specimens and the absence of reliably associated males for Cuban specimens. See the remarks under *M. cubae* for comments on COI sequence divergence between these two species.

**Figure 22.**
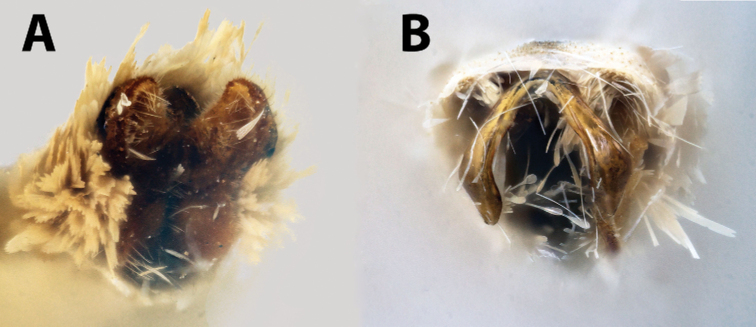
*Claduncaria maestrana* external genitalia. **A** female **B** male.

**Figure 23.**
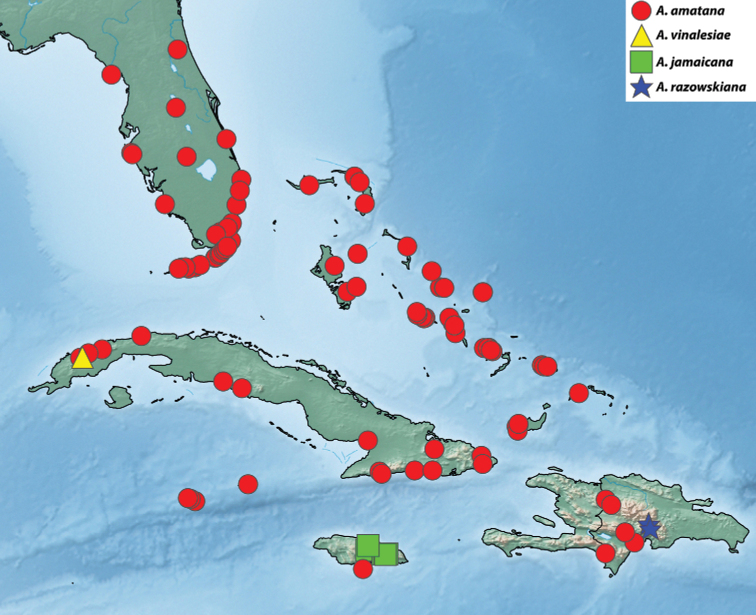
*Argyrotaenia* species distributions. Legend inset.

#### Mictopsichia jamaicana

Taxon classificationAnimaliaLepidopteraTortricidae

Razowski, 2009

E7434A14-20D4-52A7-A2CA-2A36CE30EAF2

Fig. 25C

Mictopsichia jamaicana Razowski, 2009: 238, figs 39, 55

##### Diagnosis.

*Mictopsichia jamaicana* (Razowski 2009: fig. 55) cannot be confused with any other species. The large black tornal patch on the dorsal surface of the forewing distinguishes it from all other Neotropical telechromatic tortricines.

##### Type material.

***Holotype*** ♀: **Jamaica: [St. Thomas]: Corn Puss Gap**, 19 vii 1936, Avinoff & Shoumatoff [could not locate, figure examined], genitalia slide #12363 [could not locate, figure examined] (CMNH).

##### Description.

**Male.** Male unknown.

**Female.** See Razowski (2009).

##### Distribution.

Jamaica (Fig. 25C).

##### Ecology.

Nothing is known of its biology. Like other species of the *Mictopsichia* group of genera, *Mictopsichia jamaicana* is presumed to be diurnal but may also come to lights. The holotype was collected in July.

##### Remarks.

The holotype and genitalia slide of *Mictopsichia jamaicana* could not be located in CMNH. It may still be with Razowski in ISEZ. The wing pattern is more similar to *Mictocommosis godmani* (Walsingham, 1914) and *Mictocommosis lesleyae* than to any described *Mictopsichia*. We suspect it may belong to the Neotropical group of *Mictocommosis* rather than *Mictopsichia* (see remarks under *Mictocommosis lesleyae*), but choose to retain it in *Mictopsichia* in the absence of known males and having been unable to examine the holotype. *Mictopsichia jamaicana* is known only from the holotype collected over 80 years ago. Further searching should be conducted to confirm its continued existence on Jamaica.

**Figure 24.**
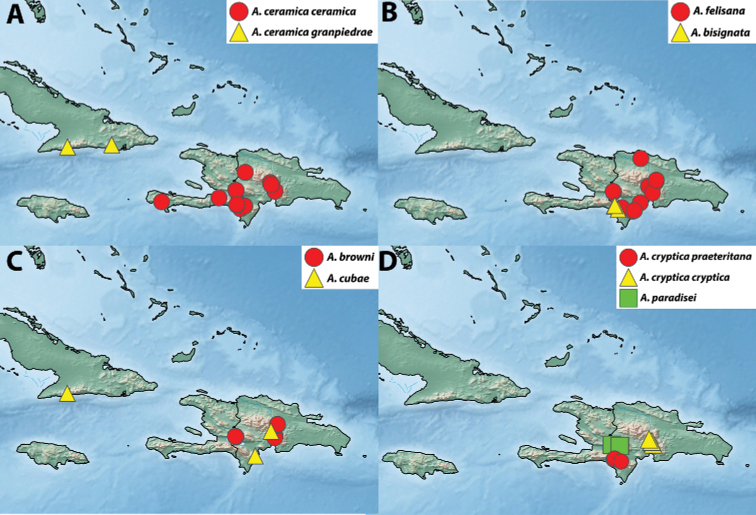
*Argyrotaenia* species distributions. Legends inset.

### Checklist of Caribbean Archipini

As part of this checklist we also include the (1) type locality as the country and state or province (if known), (2) the institutional abbreviation where primary type(s) are deposited, and (3) the sex of the primary type(s). All names considered valid in this paper are listed in boldface italicized type; synonyms, unavailable names, and subsequent misspellings are given in regular italicized type. Unavailable names are denoted by the “‡” symbol. Type species of genera are denoted by an asterisk. New taxonomic proposals are given in boldface type.

***Argyrotaenia*** Stephens, 1852: 67 (type species: *Tortrix politana* Haworth, [1811])

*Argyrothaenia*‡ in Diakonoff 1939 (misspelling): 190

*Subargyrotaenia* Obraztsov, 1961: 38 (type species: *Tortrix purata* Meyrick, 1932)

***amatana*** Dyar, 1901 (*Lophoderus*): 24 (USA: Florida, USNM)

*chioccana* Kearfott, 1907 (*Tortrix*): 72 (USA: Florida, AMNH)

*chiococcana* Meyrick, 1912, in Wagner (*Argyrotoxa*): 52; unjustified emendation of *chioccana*

*neibana* Razowski, 1999, **syn. nov.** (*Argyrotaenia*): 310 (Dominican Republic: Bahoruco, CMNH)

*ochrochroa* Razowski, 1999, **syn. nov.** (*Argyrotaenia*): 310 (Turks & Caicos: Providenciales, CMNH)

*ochrotona*‡ in Razowski and Becker 2000a: 312 (misspelling of *ochrochroa*)

***bisignata*** Razowski, 1999 (*Argyrotaenia*): 310 (Dominican Republic: Pedernales, CMNH)

***browni* sp. nov.** (*Argyrotaenia*): 41 (Dominican Republic: Independencia, CMNH)

***ceramicaceramica*** Razowski, 1999 (*Argyrotaenia*): 309 (Dominican Republic: Pedernales, CMNH)

***ceramicagranpiedrae*** Razowski & Becker, 2010, **stat. nov.** (*Argyrotaenia*): 17 (Cuba: Santiago de Cuba, VBC^5^)

***cryptica* sp. nov.** (*Argyrotaenia*): 47(Dominican Republic: La Vega, CMNH)

***crypticacryptica* ssp. nov.** (Argyrotaenia): 48 (Dominican Republic: La Vega, CMNH)

***crypticapraeteritana* ssp. nov.** (*Argyrotaenia*): 51 (Dominican Republic: Pedernales, CMNH)

*cineriptera*‡ Razowski, unavailable manuscript name

***cubae*** Razowski & Becker, 2010 (*Argyrotaenia*): 13 (Cuba: Santiago de Cuba, VBC^5^)

***felisana*** Razowski, 1999 (*Argyrotaenia*): 309 (Dominican Republic: Independencia, CMNH)

*felizana*‡ in Razowski 1999 (misspelling): 309

***flavoreticulana*** Austin & Dombroskie, 2019 (*Argyrotaenia*): 9 (The Bahamas: Great Exuma, CUIC)

***jamaicana*** Razowski & Becker, 2000b (*Argyrotaenia*): 313 (Jamaica: ? Portland, CMNH)

*partheniana*‡ unattributed, unavailable manuscript name

***kimballi*** Obraztsov, 1961 (*Argyrotaenia*): 13 (USA: Florida, AMNH)

***nuezana*** Razowski, 1999 (*Argyrotaenia*): 309 (Dominican Republic: La Vega, CMNH)

*nuesana*‡ in Razowski 1999 (misspelling): 317

***paradisei* sp. nov.** (*Argyrotaenia*): 53 (Dominican Republic: Independencia, CMNH)

***razowskiana* sp. nov.** (*Argyrotaenia*): 44 (Dominican Republic: La Vega, CMNH)

***vinalesiae*** Razowski & Becker, 2010 (*Argyrotaenia*): 13 (Cuba: Pinar del Río, VBC^5^)

***Claduncaria*** Razowski, 2000, in Razowski & Becker, 2000a: 208 (replacement name) (type species: *Cladotaenia ochrochlaena* Razowski, 1999

*Cladotaenia*‡, Razowski, 1999: 312 (preoccupied by Cohn, 1901)

*mesosignaria* group

***chalarostium*** (Razowski & Becker, 2000b), **comb. nov.**, **stat. nov.** (*Argyrotaenia*): 315 (Jamaica: ? Portland, CMNH)

***mesosignaria*** (Razowski, 1999), **comb. nov.** (*Argyrotaenia*): 311 (Dominican Republic: La Vega, CMNH)

*thamaluncus* Razowski, 1999, **syn. nov.** (*Argyrotaenia*): 311 (Dominican Republic: La Vega, CMNH)

*Clepsis mesosignaria*, error in figure of Razowski & Becker, 2010: 37

***minisignaria*** (Razowski, 1999), **comb. nov.** (*Argyrotaenia*): 311 (Dominican Republic: Pedernales, CMNH)

***rufochlaena*** Razowski & Becker, 2000a (*Claduncaria*): 208 (Jamaica: ? Portland, CMNH)

***taino* sp. nov.** (*Claduncaria*): 76 (Dominican Republic: La Vega, CMNH)

*ochrochlaena* group

***maestrana*** Razowski & Becker, 2010: 11 (Cuba: Santiago de Cuba, VBC^5^)

*labisclera* Razowski & Becker, 2010, **syn. nov.** (*Clepsis*): 20 (Cuba: Santiago de Cuba, VBC^5^)

ochrochlaena* (Razowski, 1999) (*Cladotaenia*): 312 (Dominican Republic: Pedernales, CMNH)

***praedictana* sp. nov.** (*Claduncaria*): 68 (Dominican Republic: Monseñor Nouel, CMNH)

***rawlinsana* sp. nov.** (*Claduncaria*): 66 (Dominican Republic: Pedernales, CMNH)

***Clepsis*** Guenée, 1845: 149 (type species: *Tortrix rusticana* Hübner [1796–1799] *sensu* Treitschke, 1830 [=*Tortrix senecionana* Hübner, [1818–1819])

*Smicrotes* Clemens, 1860: 355 (type species: *Smicrotes peritana* Clemens, 1860)

*Siclobola* Diakonoff, 1948: 25 (type species: *Tortrix unifasciana* Duponchel, 1842)

*Pseudamelia* Obraztsov, 1954: 196 (type species: *Tortrix unicolorana* Duponchel, 1835) [described as a subgenus of *Clepsis*]

*Clepsodes* Diakonoff, 1957: 240 (type species: *Clepsis tetraplegma* Diakonoff, 1957) [described as a subgenus of *Clepsis*]

*Mochlopyga* Diakonoff, 1964: 44 (type species: *Tortrix humana* Meyrick, 1912)

***davisi* sp. nov.** (*Clepsis*): 86 (Guadeloupe, Saint-Claude, CUIC)

***deroni* sp. nov.** (*Clepsis*): 79 (Dominican Republic: San José de Ocoa, CMNH)

***jamesstewarti* sp. nov.** (*Clepsis*): 83 (Dominican Republic: Pedernales, CMNH)

*developa*‡ Meyrick, unpublished manuscript name?

***peritana*** (Clemens, 1860) (*Smicrotes*): 356 (“Canada and USA”, ANSP)

*inconclusana* Walker, 1863 (*Dichelia*): 318. (“North America”, BMNH)

*pinaria* Razowski & Becker, 2010, **syn. nov.** (*Clepsis*): 22 (Cuba: Pinar del Río, VBC^5^)

***peroniae* sp. nov.** (*Clepsis*): 89 (Dominican Republic: Elías Piña, USNM).

***Mictocommosis*** Diakonoff, 1977: 8 (type species: *Simaethis nigromaculata* Issiki, 1930)

***lesleyae* sp. nov.** (*Mictocommosis*): 92 (Dominican Republic: Azua, CMNH)

***Mictopsichia*** Hübner, [1825] 1816: 374 (type species: Phalaena (Tortrix) hubneriana Stoll, 1791)

*Micropsichia*‡ in Agassiz, 1848: 674 (misspelling)

*Micropsychia*‡ in Agassiz, 1848: 674 (misspelling)

*Mictopsychia*‡ in Riley, 1889: 158 (misspelling)

*Mictropsichia*‡ in Heppner, 1978: 53 (misspelling)

***cubae*** Razowski, 2009 (*Mictopsichia*): 227 (Cuba: Santiago de Cuba, BMNH)

***jamaicana*** Razowski, 2009 (*Mictopsichia*): 238 (Jamaica: St. Thomas, CMNH)

***nyhllinda* sp. nov.** (*Mictopsichia*): 99 (Dominican Republic: Hato Mayor, CMNH)

***Rubropsichia*** Razowski, 2009: 240 (type species: *Rubropsichia brasiliana* Razowski, 2009)

***santaremana*** Razowski, 2009 (*Rubropsichia*): 242 (Brazil: Pará, CMNH)

### Geographic Checklist of Caribbean Archipini by Island or Archipelago

* = endemic

**Cayman Islands**

*Argyrotaenia amatana*

**Cuba**

*Argyrotaenia amatana*

A. ceramica granpiedrae*, **stat. nov.**

*A. cubae*

A. vinalesiae*

Claduncaria maestrana*

*Clepsis peritana*

*Mictopsichia cubae*

*Mictop. nyhllinda*, **sp. nov.**

**Dominica**

*Clepsis davisi*, **sp. nov.**

**Grenada**

*Rubropsichia santaremana*

**Guadeloupe**

*Clepsis davisi*, **sp. nov.**

**Hispaniola**

*Argyrotaenia amatana*

A. bisignata*

A. browni*, **sp. nov.**

A. ceramica ceramica*

A. cryptica cryptica*, **ssp. nov.**

A. cryptica praeteritana*, **ssp. nov.**

*A. cubae*

A. felisana*

A. nuezana*

A. paradisei*, **sp. nov.**

A. razowskiana*, **sp. nov.**

Claduncaria mesosignaria*, **comb. nov.**

Cla. minisignaria*, **comb. nov.**

Cla. ochrochlaena*

Cla. praedictana*, **sp. nov.**

Cla. rawlinsana*, **sp. nov.**

Cla. taino*, **sp. nov.**

Clepsis deroni*, **sp. nov.**

Cle. jamesstewarti*, **sp. nov.**

Cle. peroniae*, **sp. nov.**

Mictocommosis lesleyae*, **sp. nov.**

*Mictopsichia cubae*

*Mictop. nyhllinda*, **sp. nov.**

**Jamaica**

*Argyrotaenia amatana*

A. jamaicana*

Claduncaria chalarostium*, **comb. nov.**, **stat. nov.**

Cla. rufochlaena*

Mictopsichia jamaicana*

**Lucayan Archipelago**

**(The Bahamas, Turks & Caicos)**

*Argyrotaenia amatana*

A. flavoreticulana*

*A. kimballi*

*Clepsis peritana*

## Discussion

COI sequence data strongly suggest the presence of at least four groups of *Argyrotaenia* in the Caribbean (Figs 3, 4). Further sampling, especially of Central American species, would be required to determine group monophyly and establish their relationships to mainland taxa.

The first group, consisting of *A. amatana*, *A. vinalesiae*, and *A. jamaicana*, is primarily coastal (except *A. jamaicana*, which is a mid- to high elevation species), externally distinct from one another, but possess very similar genitalia. Representatives of this group are found in The Bahamas, the Cayman Islands, Cuba, Hispaniola, Jamaica, and the Turks & Caicos islands. Based on similarities in genitalia and its low-elevation distribution, *A. flavoreticulana* may also belong to this group, but COI barcodes were not available for it. Our Maximum Likelihood analysis suggests that this group is distinct from the rest of the Caribbean *Argyrotaenia* + *Claduncaria*. The second group, consisting of *A. ceramica*, is very distinct, both externally and in genitalia, from the rest of the Caribbean *Argyrotaenia*. It appears to be closely allied to the *A. ponera* group (Brown and Cramer 1999) of Mexico and the southwestern United States. It is found on both Cuba and Hispaniola. The third group, consisting of *A. cubae* and *A. browni*, are remarkably similar externally, but distinct in both male and female genitalia. Representatives of this group are found on both Cuba and Hispaniola. The fourth group may represent an exclusively Hispaniolan radiation of *Argyrotaenia*, but further sampling would be necessary to confirm its monophyly. It consists of six species: *A. nuezana*, *A. razowskiana*, *A. bisignata*, *A. felisana*, *A. paradisei*, and *A. cryptica*. All these species are relatively distinct from one another in forewing pattern and male genitalia, but less so in female genitalia.

Our Maximum Likelihood analysis (Fig. 4) recovers *Claduncaria*, as we redefine it, to be monophyletic with moderate support, with two subclades with similar compositions to our *mesosignaria* and *ochrochlaena* groups. The two exceptions to this are *Cla. chalarostium* and *Cla. rufochlaena*, which based on COI, are placed in the *ochrochlaena* group. Further studies are warranted to elucidate relationships within *Claduncaria*. Our analysis suggests that *Claduncaria* may be a highly derived Caribbean lineage of *Argyrotaenia*. This putative relationship and its taxonomic status also warrant further investigation.

The relationships among Caribbean *Clepsis* are much less clear. Our Maximum Likelihood analysis (Fig. 4) gives little resolution, as the genus is shown to be a grade rather than monophyletic. The genus as a whole is in need of revision, so any proposed relationships to mainland taxa are purely speculative at this point.

Nothing can be said of the *Mictopsichia* group of genera as they were used as outgroups in our analyses. The relationships of these genera to Archipini, to each other, and to Tortricidae as a whole deserve careful future examination in the future.

### Hypothesized coupling mechanism in *Claduncaria*

The functional morphology of Lepidoptera genitalia is poorly understood (Cordero and Baixeras 2015). Part of the reason for this is the extreme variation in the size, shape, development, and presence/absence of different genitalic structures across the order and even within genera and species (Gilligan and Wenzel 2008). Genitalic structures that serve an important copulatory function in one group of Lepidoptera may be reduced or lost entirely in a different group. Even if structures do serve the same function, they may act on a different structure in the opposite sex to accomplish it. For this reason, a generalized functional morphology for Lepidoptera genitalia is not possible beyond perhaps a few highly conserved structures. Published instances of precise structural interactions during copulation are few, scattered and often restricted to large, showy, or economically important species. For a detailed account of modern understanding as well as historical overview of the functional morphology of Lepidoptera genitalia, see Cordero and Baixeras (2015).

Precise coupling mechanisms in Tortricidae are even more poorly known, having only been investigated or hypothesized in a few instances (Ferro and Akre 1975; Pérez Santa-Rita and Baixeras 2017), despite recent interest in more generalized functional morphology (Lincago et al. 2013; Anzaldo et al. 2014; Zlatkov 2016) and intraspecific variation (Mutaten et al. 2007; Gilligan and Wenzel 2008; Rentel et al. 2017).

A putative autapomorphy for the family Tortricidae is the presence of flattened papillae anales, a feature modified in some plesiomorphic groups, presumably to facilitate oviposition (Horak 1999). To our knowledge, the hypothesized coupling method presented here for *Claduncaria* Razowski is only the third proposed sexual coupling mechanism for Tortricidae (Ferro and Akre 1975; Pérez Santa-Rita and Baixeras 2017), the second where interactions take place externally (Pérez Santa-Rita and Baixeras 2017), and the first which describes interspecific differences.

In situ, the ventroposterior portion of the papillae anales of the *ochrochlaena* group form a cup-like structure (Fig. 22A). Males have a divergently bifurcate uncus (Fig. 22B) which can be experimentally inserted between the papillae anales under the microscope to “couple” the two. The size/width of the cup of the papillae anales (referred to as a groove when flattened under glass for photography) seem to correspond well to the shape/width of the arms of the uncus of the male for each species in this group. For example, the grooves in the papillae anales and arms of the uncus in *Cla. ochrochlaena* are narrow (Figs 18B, 19B), whereas those of *Cla. maestrana* (Figs 18A, 19A) are much wider.

In the *ochrochlaena* group, we hypothesize that the uncus is inserted between the papillae anales and acts to push the papillae anales into the abdominal cavity, thereby exposing the ostium to allow for insertion of the phallus and eversion of the vesica, similar to the generalized strategy outlined in Cordero and Baixeras (2015). Simultaneously, the terminal plate of the gnathos may fit into a corresponding structure (e.g., a “pocket”) between the anterior portion of the papillae anales. Such a deep pocket has been observed in *Cla. ochrochlaena* and likely occurs in other females of the *ochrochlaena* group. We suspect careful histological work in females may be necessary to investigate this further. The gnathos is distinctly articulated in the *ochrochlaena* group, suggesting that musculature may be more strongly developed in these species and may serve additional functions, perhaps even acting independently of the uncus to “pry open” the sterigma for copulation. With such odd and divergent shapes in the terminal plate of the gnathos in this group, it is difficult to imagine that it does not serve some sort of copulatory function.

Females of the *mesosignaria* group, in contrast, have massively swollen papillae anales (Fig. 19E–G) and males have a correspondingly large apex of the uncus (Fig. 18E–G). *Cla. rufochlaena*, for which the female is unknown (but see remarks under its species account) is the exception to this. Even though it is undoubtedly a member of the *mesosignaria* group based on the shared valvae and gnathos structure, it possesses a divergently bifid uncus. Excluding *Cla. rufochlaena*, we hypothesize the uncus in the *mesosignaria* group serves a similar function as in the *ochrochlaena* group; that is, to push the papillae anales into the abdominal cavity and thus expose the ostium.

Whether these structures arose as part of a sexual arms race between the sexes or as an adaptation to a novel ovipositional strategy is yet to be seen (Cordero and Baixeras 2015). The host preference(s) for this genus is not yet known, but we hypothesize it must be very unusual in members of the *ochrochlaena* group if ovipositional strategy is the mechanism driving its evolution. That males seem to have adapted alongside females in both of these groups is certainly worthy of further study, both from a morphological and evolutionary perspective.

### Biogeographical note on Puerto Rico

Interestingly, no species of Archipini are known from Puerto Rico. We did dissect a pair of tortricids that superficially resembled *Clepsis*, but they turned out to be *Coelostathma parallelana* Walsingham, 1897 (Sparganothini), a widespread Caribbean species. We do not think the absence of Archipini on Puerto Rico is an artifact of a lack of collecting, as the island is among the most well-collected for insects in the Caribbean, including microlepidoptera (e.g., Forbes 1931). In fact, Puerto Rico is listed as the type locality of no fewer than 23 species of tortricids (Brown 2005). Why then, is Puerto Rico so depauperate when it comes to Archipini?

**Figure 25.**
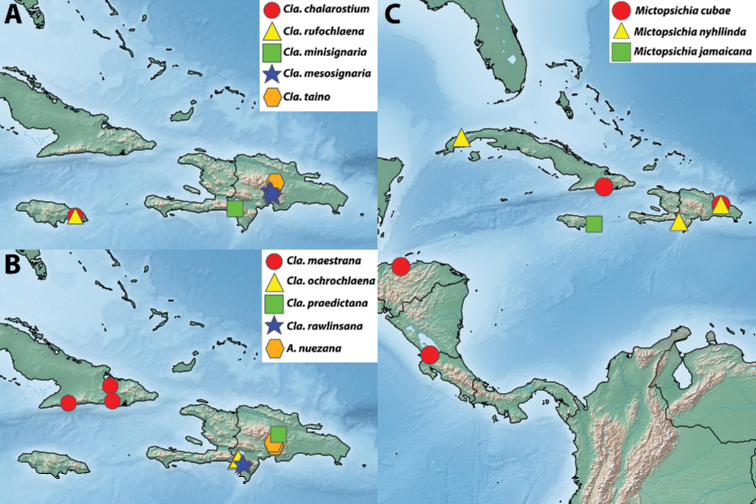
*Argyrotaenia*, *Claduncaria* and *Mictopsichia* species distributions. Legends inset.

**Figure 26.**
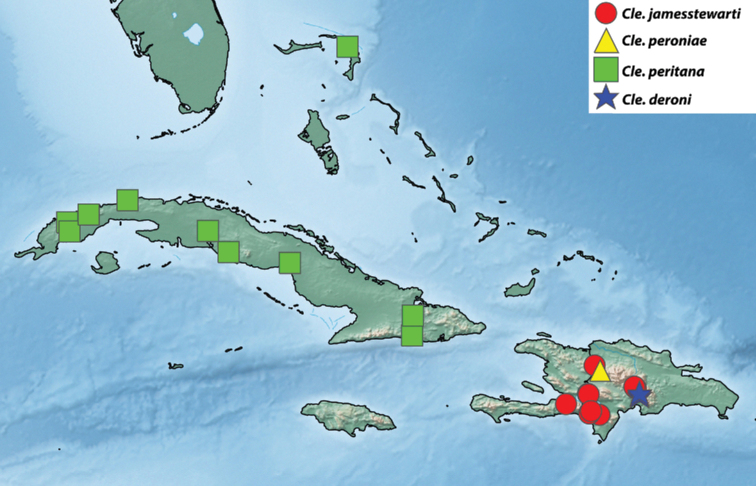
*Clepsis* species distributions. Legend inset. Florida records of *Cle. peritana* omitted.

For most insect taxa, particularly montane species, overall species diversity decreases in the Greater Antilles from west to east and has been well-documented by entomologists (Liebherr 1988 and references therein). Possible explanations for this were first laid out by Martorell (1945). First, Puerto Rico’s positioning in the Caribbean (farthest east of the Greater Antilles and farthest north of the Lesser Antilles) act as a barrier to colonization. Second, trade winds coming from the east makes overall dispersal, especially by weak flyers, difficult. Lastly, deforestation may have contributed to the local extinction of some species, although we have no evidence Archipini occurred in Puerto Rico in the past. Suitable habitat certainly exists on Puerto Rico, but with no archipines present on the low-elevation east coast of Hispaniola (see Figs 23–26) nor in the northern half of the Lesser Antilles, distance may have been enough of a barrier to have prevented colonization.

Puerto Rico and northern Hispaniola were connected until the formation of the Mona Passage (~30 to 20 mya; Van Gestel et al. 1999; MacPhee et al. 2003), suggesting that colonization of the Caribbean by Archipini may not have occurred until after the two islands became separated.

**Figure 27.**
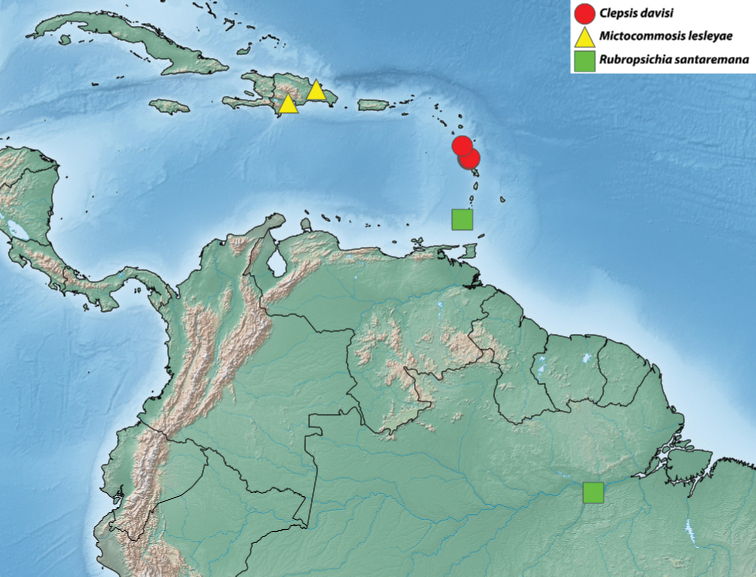
*Clepsis*, *Mictocommosis* and *Rubropsichia* species distributions. Legend inset.

## Supplementary Material

XML Treatment for Argyrotaenia

XML Treatment for Argyrotaenia ceramica

XML Treatment for Argyrotaenia ceramica ceramica

XML Treatment for Argyrotaenia ceramica granpiedrae

XML Treatment for Argyrotaenia vinalesiae

XML Treatment for Argyrotaenia jamaicana

XML Treatment for Argyrotaenia amatana

XML Treatment for Argyrotaenia flavoreticulana

XML Treatment for Argyrotaenia kimballi

XML Treatment for Argyrotaenia bisignata

XML Treatment for Argyrotaenia felisana

XML Treatment for Argyrotaenia nuezana

XML Treatment for Argyrotaenia cubae

XML Treatment for Argyrotaenia browni

XML Treatment for Argyrotaenia razowskiana

XML Treatment for Argyrotaenia cryptica

XML Treatment for Argyrotaenia cryptica cryptica

XML Treatment for Argyrotaenia cryptica praeteritana

XML Treatment for Argyrotaenia paradisei

XML Treatment for Claduncaria

XML Treatment for Claduncaria maestrana

XML Treatment for Claduncaria ochrochlaena

XML Treatment for Claduncaria rawlinsana

XML Treatment for Claduncaria praedictana

XML Treatment for Claduncaria mesosignaria

XML Treatment for Claduncaria minisignaria

XML Treatment for Claduncaria chalarostium

XML Treatment for Claduncaria rufochlaena

XML Treatment for Claduncaria taino

XML Treatment for Clepsis

XML Treatment for Clepsis deroni

XML Treatment for Clepsis jamesstewarti

XML Treatment for Clepsis davisi

XML Treatment for Clepsis peritana

XML Treatment for Clepsis peroniae

XML Treatment for Rubropsichia

XML Treatment for Rubropsichia santaremana

XML Treatment for Mictocommosis

XML Treatment for Mictocommosis lesleyae

XML Treatment for Mictopsichia

XML Treatment for Mictopsichia cubae

XML Treatment for Mictopsichia nyhllinda

XML Treatment for Mictopsichia jamaicana

## Footnotes

male of Mictopsichia jamaicana and females of Rubropsichia spp. are unknown.

Males of Cla. chalarostium and Cla. praedictana unknown; females of Cla. rufochlaena and Cla. taino unknown.

Male of Cle. peroniae sp. nov. unknown (but see remarks under that species account).

The male of Mictopsichia jamaicana is unknown. This species likely belongs to Mictocommosis (see remarks under Mictocommosis lesleyae).

Types were found in ISEZ, not VBC as listed in Razowski and Becker (2010).

## Supplementary materials

Supplementary material 1 Specimens examined and COI pairwise distance matrix Data type occurences, pairwise distance matrix Explanation note Tab 1 is a list of all specimens examined and COI barcode voucher data, tab 2 is a pairwise data matrix for all barcoded specimens. https://binary.pensoft.net/file/470528This dataset is made available under the Open Database License (http://opendatacommons.org/licenses/odbl/1.0/). The Open Database License (ODbL) is a license agreement intended to allow users to freely share, modify, and use this Dataset while maintaining this same freedom for others, provided that the original source and author(s) are credited.Kyhl A. Austin, Jason J. Dombroskie
